# In Situ NMR and Kinetics Reveal Origins of Regioselectivity Differences for Epichlorohydrin Ring‐Opening in Lewis and Brønsted Acid Zeolites

**DOI:** 10.1002/anie.202511944

**Published:** 2025-10-27

**Authors:** David S. Potts, Huston Locht, Sungmin Kim, Johannes A. Lercher, Jian Zhi Hu, David W. Flaherty

**Affiliations:** ^1^ Department of Chemical and Biomolecular Engineering University of Illinois Urbana‐Champaign Urbana Illinois 61801 USA; ^2^ School of Chemical and Biomolecular Engineering Georgia Institute of Technology Atlanta Georgia 30332 USA; ^3^ Institute for Integrated Catalysis Pacific Northwest National Laboratory Richland Washington 99352 USA; ^4^ Department of Chemistry and Catalysis Research Center TU München Lichtenbergstrasse 4 85748 Garching Germany; ^5^ The Gene & Linda Voiland School of Chemical Engineering and Bioengineering Washington State University Pullman WA 99164 USA

**Keywords:** Catalytic mechanisms, *Operando* ^13^C NMR, Ring opening, Solid–liquid interfaces, Zeolites

## Abstract

Altering the quantities and organization of reactive species at active sites enables control of turnover rates and regioselectivities (rate ratios) for ring‐opening of epichlorohydrin (C_3_H_5_ClO) across two orders of magnitude. Kinetic analysis suggests that parallel monomolecular (S_N_1) and bimolecular (S_N_2) substitution mechanisms contribute to observed rates of C_3_H_5_ClO reactions with methanol (CH_3_OH) over both Lewis (Sn‐BEA) and Brønsted acid (Al‐BEA) zeolites in liquid solvents. In situ solid‐state ^13^C‐nuclear magnetic resonance spectroscopy (SS‐NMR) measurements give direct evidence for the proposed ring‐opened carbocations and activated CH_3_OH intermediates over these catalysts. Interpretation of time‐resolved *operando* ^13^C‐SS‐NMR spectra shows that C_3_H_5_ClO‐derived carbocations and CH_3_OH‐derived surface species convert to ring‐opening products through S_N_1 and S_N_2 reaction mechanisms and subsequently form distinct product regioisomers. These NMR spectra also reveal a concomitant shift from S_N_1 to S_N_2 reactions with increases in the coverage of CH_3_OH‐derived reactive intermediates achieved by control of the local concentrations of CH_3_OH, C_3_H_5_ClO, and diluting CH_3_CN. This knowledge provides new insight into the role of coverage on regioselectivity and rates of catalytic reactions of organic species at solid–liquid interfaces.

## Graphical abstract

Regioselectivities for epichlorohydrin ring‐opening over Brønsted and Lewis acid zeolites change by an order of magnitude upon replacing fractions of methanol with acetonitrile co‐solvent. Combining transient operando ^13^C‐NMR with traditional kinetic measurements reveals that these differences originate from competition between an S_N_1 pathway through ring‐opened carbocation intermediates formed from epichlorohydrin and an S_N_2 pathway through a methanol‐derived intermediate. This work provides spectroscopic evidence for epoxide ring‐opening intermediates and mechanisms not previously established over solid catalysts.

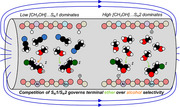

## Introduction

Epoxides serve as useful intermediates for a variety of important materials.^[^ ^1^ ^]^ The desired product from epoxide ring‐opening reactions often depends on the application; as one example, the terminal ethers and terminal alcohols produced from epoxide ring‐opening with alcohol nucleophiles serve as useful building blocks for antifreeze and polyurethanes, respectively.^[^ ^2^, ^3^ ^]^ While industrial processes have established effective strategies for developing certain desired products,^[^ ^4^, ^5^, ^6^, ^7^, ^8^ ^]^ an understanding of the relationship between product distribution and the structure of reactants, catalysts, and solvents remains unestablished.

Organic chemistry conventions suggest that the structure and functional groups of epoxides and nucleophiles limit the range of achievable regioselectivities for epoxide ring‐opening reactions.^[^ ^9^, ^10^ ^]^ Unfunctionalized terminal aliphatic epoxides (e.g., 1,2‐epoxybutane in Scheme S1) give selectivities between 50%–60% towards terminal ethers during reactions with alcohol nucleophiles.^[^ ^11^, ^12^, ^13^, ^14^ ^]^ In contrast, the ring‐opening of styrene oxide predominantly provides selectivities over 90% to terminal alcohols because the phenyl group stabilizes the positive charge on the primary epoxide carbon during nucleophilic attack,^[^ ^15^, ^16^, ^17^, ^18^ ^]^ while epichlorohydrin (C_3_H_5_ClO) ring‐opens with more than 90% selectivity towards terminal ethers because the chlorine atom destabilizes positive charge.^[^ ^13^, ^19^, ^20^, ^21^ ^]^ Brønsted acid catalysts generally provide lower regioselectivities towards terminal ethers than Lewis acids,^[^ ^13^, ^14^, ^15^, ^22^, ^23^ ^]^ but the regioselectivity differences between these acid types (< 10%) fall well below the differences observed across epoxides. Pharmaceutical processes may functionalize epoxides with directing groups that promote desired products^[^ ^24^, ^25^ ^]^ but increase process waste. Therefore, alternative methods to control regioselectivity can aid in the design of less environmentally impactful processes.

Recent work reveals that manipulations of catalyst structure and solvation environment extend the selectivity range for terminal aliphatic epoxides. The introduction of hydrogen‐bonding acceptor species (e.g., diols) increases terminal alcohol selectivities to 60%–80% for 1,2‐epoxyoctane ring‐opening over homogeneous aryl borane catalysts.^[^ ^26^, ^27^ ^]^ Work from our group reported selectivities of 40%–80% to the terminal ether for 1,2‐epoxybutane ring‐opening over *BEA zeolites through changes to the active metal, solvent composition, and zeolite silanol density.^[^ ^28^, ^29^ ^]^ While these studies provide guidelines for controlling ring‐opening regioselectivities of terminal aliphatic epoxides, similar strategies for epoxides containing directing groups have not been established. Furthermore, the physical origins of regioselectivity trends and spectroscopic evidence of the reactive intermediates during epoxide ring‐opening evade current understanding. Developing universal design principles for controlling regioselectivities requires knowledge of the ring‐opening reaction mechanism for functionalized epoxides.

Here, we demonstrate that rates and regioselectivities for C_3_H_5_ClO ring‐opening with CH_3_OH depend strongly on zeolite acid type and the fraction of acetonitrile co‐solvent. Both Brønsted (Al‐BEA) and Lewis acid (Sn‐BEA) zeolites show significant decreases in selectivity towards the terminal ether as [CH_3_OH] decreases from 24.7 M (>95%) to 0.01 M (67% for Sn‐BEA, 54% for Al‐BEA), which suggests the products may form by a different mechanism. In situ ^13^C‐nuclear magnetic resonance (NMR) spectra show that C_3_H_5_ClO forms ring‐opened carbocations over both Al‐BEA and Sn‐BEA, while CH_3_OH also forms activated surface species (i.e., protonated CH_3_OH, methoxy species). These observations provide strong evidence that C_3_H_5_ClO ring‐opening proceeds through a combination of both S_N_1 and S_N_2 reaction pathways, and the relative contributions of each pathway depend strongly on the composition of the fluid within zeolite pores. At low [CH_3_OH], the reaction proceeds by an S_N_1 pathway with ring‐opened carbocations that promote similar formation rates for both terminal ether and alcohol products. In contrast, an S_N_2 pathway dominates at high [CH_3_OH] and primarily forms the terminal ether. These findings reveal the previously unestablished mechanistic origins for regioselective ring‐opening reactions of epoxides over Lewis and Brønsted acid zeolites, demonstrating the significant selectivity implications of altering the dominant mechanism through changes in co‐solvent and reactant ratio. This work highlights the broader potential of how altering reaction microenvironments can promote desired products for nucleophilic substitutions over solid catalysts.

## Results and Discussion

### Mechanistic Interpretations for Epichlorohydrin Ring‐Opening Rates and Regioselectivities

**Figure 1:**
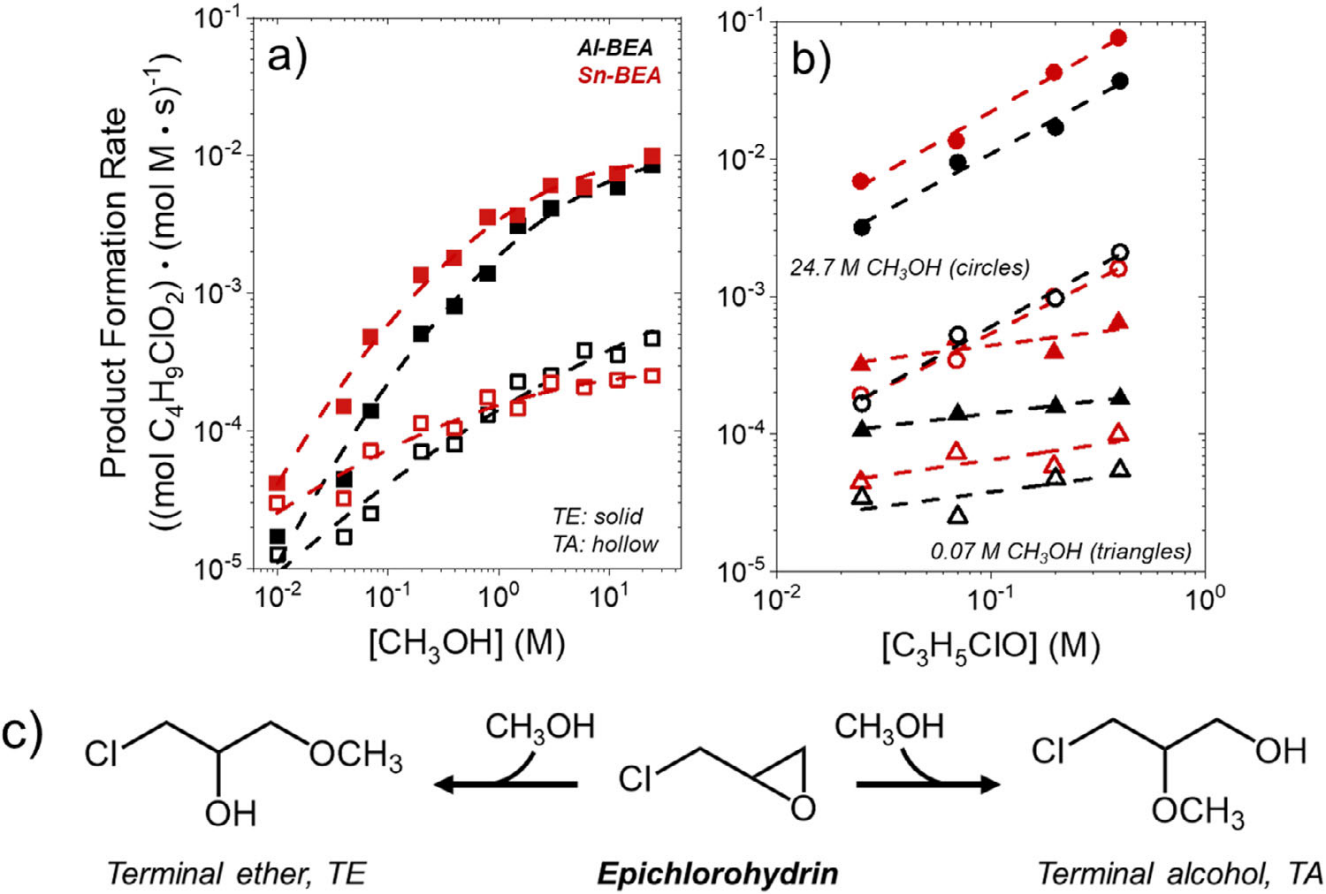
Formation rates of the terminal ether (solid points) and terminal alcohol (hollow) products for C_3_H_5_ClO ring‐opening with CH_3_OH as a function of a) CH_3_OH concentration (0.07 M C_3_H_5_ClO, CH_3_CN, 308 K) and b) C_3_H_5_ClO concentration (triangles–0.07 M CH_3_OH, circles–24.7 M CH_3_OH, CH_3_CN, 308 K) over Al‐ (black) and Sn‐BEA (red). Rates were measured from GC‐analyzed batch kinetics measurements. c) shows the reaction scheme for C_3_H_5_ClO ring‐opening with CH_3_OH.

Figure 1 demonstrates that the formation rates of the terminal ether (TE) and terminal alcohol (TA) products depend on the reactant concentrations and active metal within *BEA during C_3_H_5_ClO ring‐opening. Rates over Sn‐BEA exceed those over Al‐BEA at nearly all conditions and show a maximum difference of 5‐fold in the limit of low [CH_3_OH]. Al‐BEA and Sn‐BEA show similar product formation rate dependencies on reactant concentrations, and these changes in rates suggest both materials possess kinetic regimes in which either C_3_H_5_ClO‐ or CH_3_OH‐derived intermediates saturate active sites. Table S5 reveals that the rates to form TE show greater sensitivity to the values of [CH_3_OH] than the rates to form TA, with the largest differences appearing at the lowest ratios of [CH_3_OH] to [C_3_H_5_ClO] (e.g., when [CH_3_OH]:[C_3_H_5_ClO] < 15). In contrast, TE and TA formation rates show a nearly identical dependence on [C_3_H_5_ClO]. The differences in the TE and TA formation rates on [CH_3_OH] suggest that the two products may form by distinct mechanisms or via uniquely structured reactive intermediates whose stabilities sense the local solvation environment, causing regioselectivity to depend on [CH_3_OH] (vide infra).

**Scheme 1:**
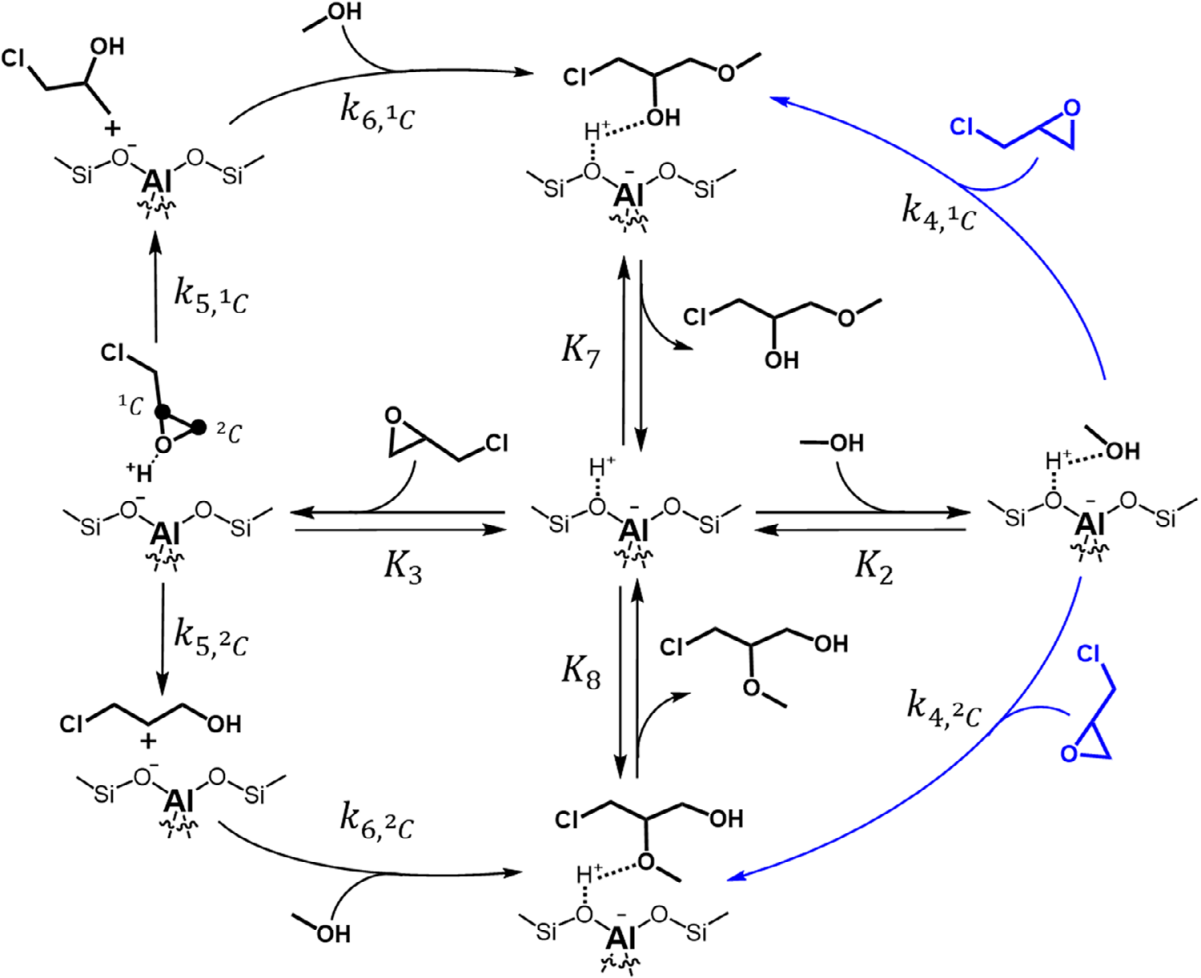
Plausible product‐forming steps for ring‐opening of C_3_H_5_ClO with CH_3_OH over Al‐BEA. Reaction may proceed through a concerted S_N_2 pathway with an activated CH_3_OH intermediate (blue) or a stepwise S_N_1 pathway in which C_3_H_5_ClO ring‐opens to form carbocations, followed by adsorption and activation of CH_3_OH (black). For brevity, we do not show the reversible adsorption of CH_3_CN molecules (step 1) in this cycle.

Organic chemistry literature suggests epoxide ring‐opening reactions proceed through a concerted bimolecular substitution (S_N_2) pathway in basic or neutral conditions, in which the nucleophile attacks the intact epoxide. Alternatively, a stepwise monomolecular (S_N_1) pathway occurs under acidic conditions and involves protonation of the epoxide to either polarize or fully open the epoxide ring before nucleophilic attack.^[^ ^9^, ^23^, ^30^ ^]^ However, the literature has not established concrete proposals for the behavior of solid catalysts. Scheme 1 presents a plausible sequence of steps for the ring‐opening of C_3_H_5_ClO with CH_3_OH over Al‐BEA, consistent with the apparent rate measurements (Figure 1) and inspired by past proposals. Products form when fluid phase C_3_H_5_ClO encounters adsorbed CH_3_OH (step 4) and reacts by an S_N_2 pathway. Alternatively, an S_N_1 pathway proceeds when C_3_H_5_ClO protonates and irreversibly ring‐opens in a monomolecular step to form primary and secondary carbocations (step 5), which undergo nucleophilic attack from CH_3_OH to form the TA and TE products, respectively (step 6). The TA and TE products desorb to complete the cycle (steps 7 and 8). Scheme S2 shows analogous proposals for S_N_1 and S_N_2 pathways over Sn‐BEA that capture past proposals based on DFT calculations^[^ ^13^ ^]^ and the recognition that Sn‐BEA contains open sites (Sn‐OH) with Brønsted acid character.^[^ ^31^ ^]^ The 3‐coordinated Sn‐OH sites likely provide greater rates than the 4‐coordinated, closed Sn sites for both S_N_1 and S_N_2 pathways, but both populations of sites likely participate in ring‐opening catalysis^[^ ^13^, ^32^ ^]^ (see end of Section S8 for full discussion of these sites).

Assumptions based on adsorption thermodynamics and concentration ratios lead to rate expressions that agree with experimental observations (Figure 1) and describe rates for S_N_1 pathways at low ratios of [CH_3_OH] to [C_3_H_5_ClO] (Equation 1) and S_N_2 pathways at high ratios (Equation 2):

(1)

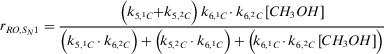

(2) $$r_{RO,S_{N}2}=k_{4}\;\left[C_{3}H_{5}ClO\right]$$

These equations predict TE and TA formation rate orders that align with the trends observed in Figure 1. See Section S8 for a full discussion of the rate law derivations.

Overall, the turnover rates (Figure 1) give evidence that M‐BEA catalysts facilitate C_3_H_5_ClO through a combination of parallel reaction pathways (Scheme 1), with the relative importance of each pathway changing with the composition of the solution. Scheme 1 and the derived rate expressions indicate that both rates and regioselectivity of epichlorohydrin conversion depend on the identity and structure of the most abundant reactive intermediates, which dictate the contributions of S_N_1 and S_N_2 pathways and the associated rates to form TA or TE products. In situ and *operando* NMR spectroscopy experiments (vide infra) give new and direct evidence that tests these proposals.

### Revealing Structure of Adsorbed CH_3_OH and C_3_H_5_ClO with ^13^C NMR Spectroscopy

**Figure 2:**
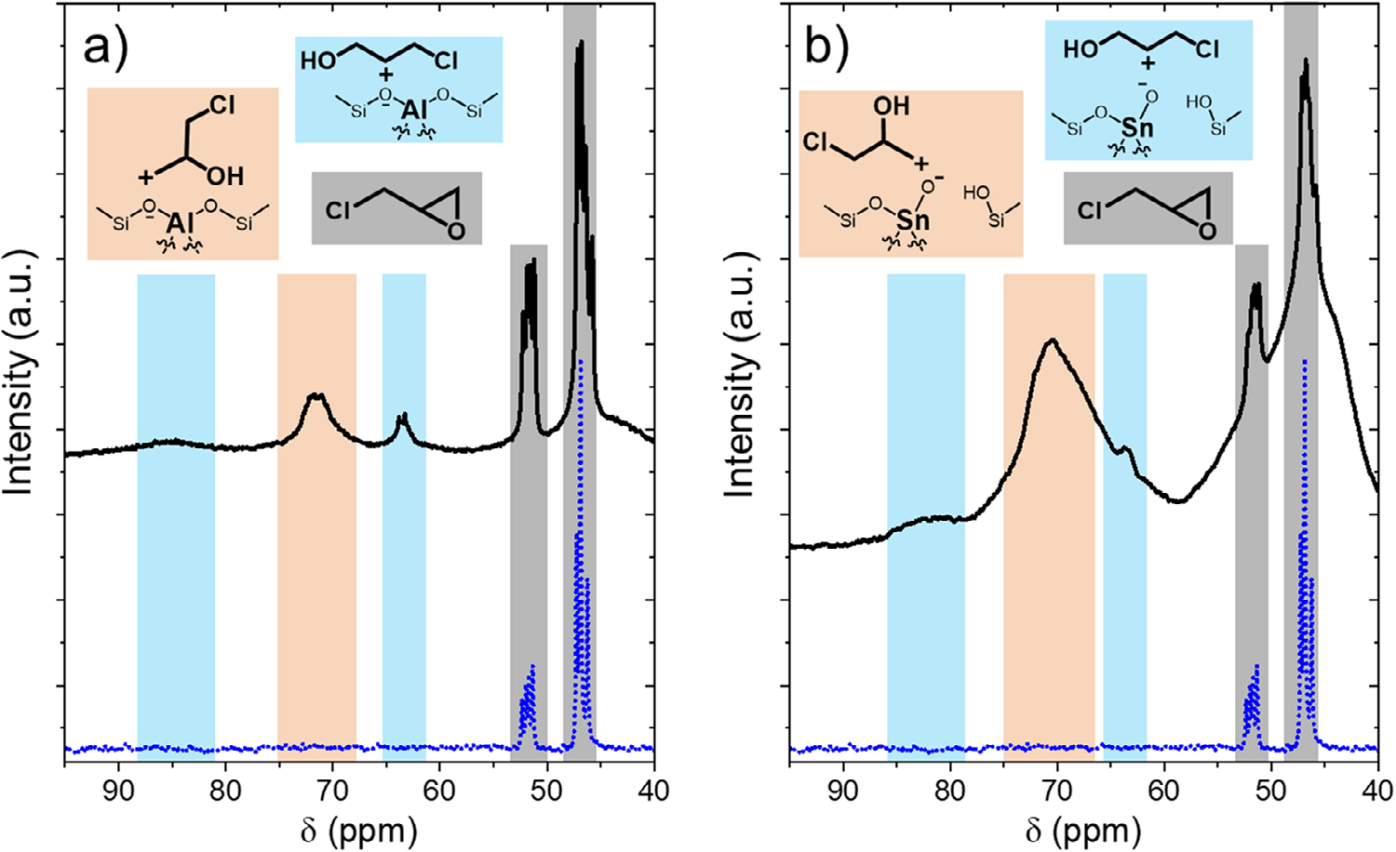
^13^C NMR cross‐polarization spectra (black lines) of ^13^C_3_H_5_ClO on a) Al‐BEA and b) Sn‐BEA (0.2 M ^13^C_3_H_5_ClO, CH_3_CN, 298 K, 12 h after initial contact between the liquid solution and the zeolite). Blue dotted lines show the single‐pulse spectra for ^13^C_3_H_5_ClO in the absence of a catalyst (average of 268 scans). CP spectra are an average of 3558 scans (Al‐BEA) and 4096 scans (Sn‐BEA). Contact time for the CP spectra was 2 ms.

Figure 2 presents in situ solid‐state cross‐polarization (CP) ^1^H‐^13^C MAS NMR spectra that reveal the adsorption structure of C_3_H_5_ClO on the surface of Al‐BEA and Sn‐BEA in CH_3_CN. Figure 2a reveals that ^13^C_3_H_5_ClO forms ring‐opened carbocations upon adsorption to the Brønsted acid sites of Al‐BEA. DFT‐predicted chemical shifts (Table S9, entries 1 and 4) agree with observed peak locations for carbons within ring‐opened primary (69–75 ppm in experiment, 71 and 75 ppm by DFT) and secondary (63, 85 ppm in experiment, 63, 89 ppm by DFT) carbocations bound at Brønsted acid sites in the absence of CH_3_OH, which shows C_3_H_5_ClO activates in a monomolecular step consistent with the proposed S_N_1 mechanism. While the CP spectra predominantly reveal chemically bound surface species, the appearance of sharp features (e.g., 45–48 and 51–52 ppm) suggests the presence of liquid‐phase C_3_H_5_ClO near the surface. The same features (45–48 and 51–52 ppm) also appear within single‐pulse (SP) ^13^C‐NMR spectra for liquid‐phase C_3_H_5_ClO (Figure 2a, dotted blue line), matching with predicted shifts for liquid‐phase C_3_H_5_ClO (Table S8). The random molecular motion in the bulk liquid phase would not yield a CP signal, meaning these features on the CP spectra must originate from C_3_H_5_ClO within the pore but not chemically bound. Upon contacting the solution mixture with Al‐BEA, the features representing liquid‐phase C_3_H_5_ClO broaden slightly in both the CP (Figures 2 and S24) and SP spectra (Figure S23), which show liquid‐phase and surface species with equal molar sensitivity. The negligible broadening and shift in peaks of C_3_H_5_ClO (45–48 and 51–52 ppm) in the presence of Al‐BEA indicate that intact C_3_H_5_ClO resides in the bulk fluid and to some extent within pores but does not remain intact upon adsorption to Brønsted acid sites,^[^ ^33^ ^]^ where C_3_H_5_ClO reacts to form ring‐opened carbocations instead.

Figure 2b supports that ^13^C_3_H_5_ClO forms similar primary (∼68–75 ppm) and secondary (63, 83 ppm) surface carbocations over Sn‐BEA. Interestingly, these peaks do not coincide with the DFT‐predicted shifts in Table S9 (entries 5–6) (67, 73 ppm for primary, 65, 76 ppm for secondary) as closely as shown for Al‐BEA. The DFT calculations predict a shift for protonated C_3_H_5_ClO at 83 ppm in the liquid phase (Table S8), but the same species coordinated at Sn sites are predicted to only show peaks between 50–54 ppm (Table S9, entry 20). Given these comparisons, we conclude that observed peaks from 60–85 ppm in Figure 2b most plausibly originate from ring‐opened carbocations that form over Sn‐BEA. These ring‐opened species appear in different relative proportions than in Al‐BEA: the ratio of primary to secondary carbocations on Sn‐BEA (2.4) is much greater than for Al‐BEA (1.4). The difference between the ratios of carbocations for each material appears consistent with the greater regioselectivity to the terminal ether on Sn‐BEA when the S_N_1 pathway dominates at low ratios of [CH_3_OH] to [C_3_H_5_ClO] (Figure 1 and vide infra). These species likely form at protic functions generated by hydrolysis of the framework Sn to create open sites (Sn‐OH, inset Figure 2b).^[^ ^31^ ^]^ Interestingly, features for C_3_H_5_ClO (45–48 and 51–52 ppm) broaden significantly upon contact with Sn‐BEA, which suggests a greater fraction of C_3_H_5_ClO adsorbs intact over Sn‐BEA. Additionally, SP spectra acquired as a function of time demonstrate that the ring‐opened carbocations form at slower rates over Sn‐BEA than Al‐BEA (Figure S23), aligning with this interpretation and suggesting that greater quantities of adsorbed C_3_H_5_ClO form ring‐opened carbocations over Al‐BEA. The lesser prevalence of carbocations over Sn‐BEA may occur because the material contains a mixture of open (Sn‐OH, 3‐coordinated) and closed (4‐coordinated) sites under reaction conditions. We hypothesize that closed Sn sites likely cannot ring‐open C_3_H_5_ClO without the participation of a nucleophile (e.g., CH_3_OH) due to the absence of a hydroxyl moiety intrinsic to the catalytic site, which prohibits the formation of ring‐opened carbocations. Overall, the capability of both Al‐BEA and Sn‐BEA to form ring‐opened carbocations gives evidence that both catalysts catalyze C_3_H_5_ClO ring‐opening through an S_N_1 reaction pathway that dominates in the limit of low [CH_3_OH].

**Figure 3:**
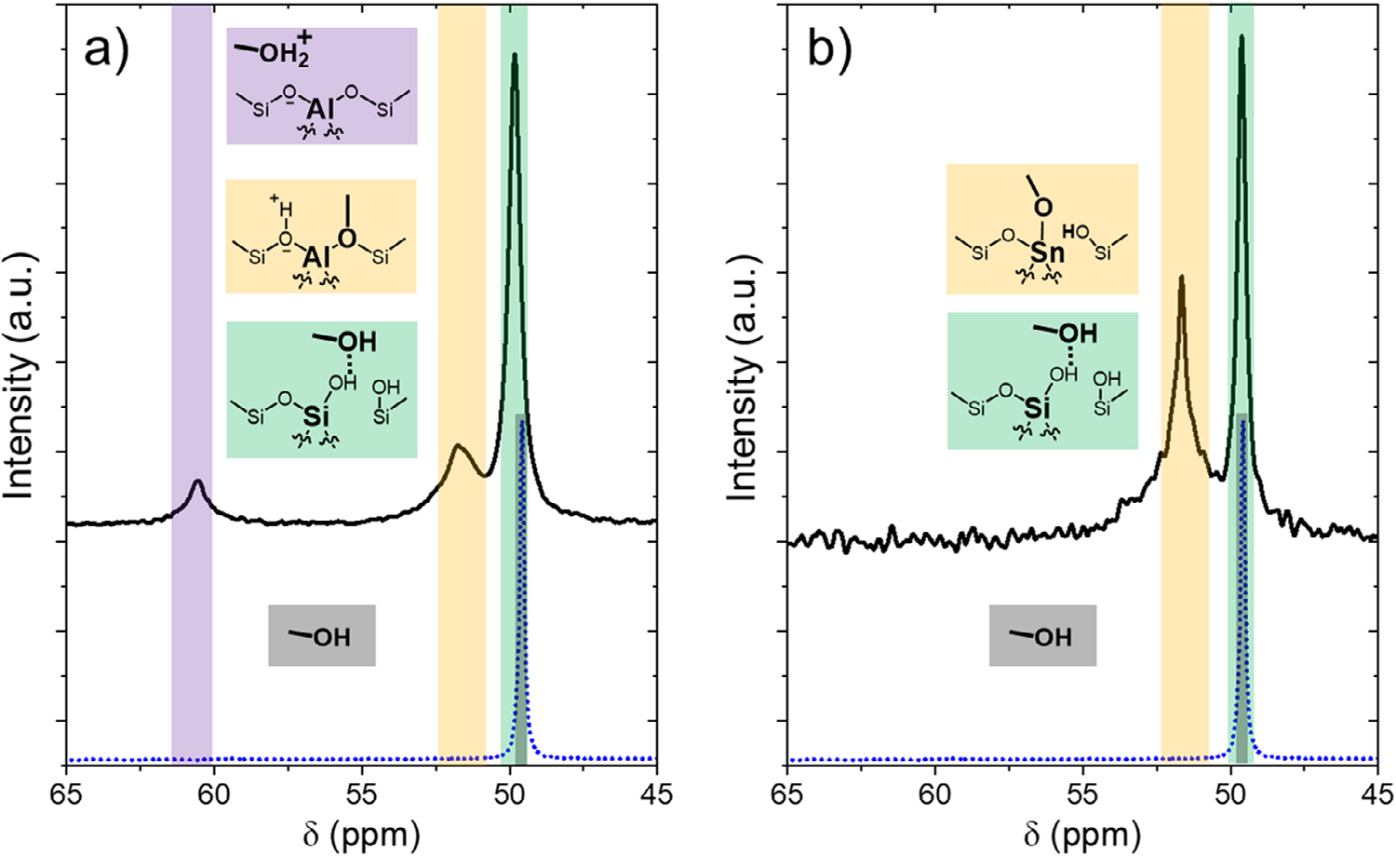
^13^C NMR cross‐polarization spectra (black lines) of ^13^CH_3_OH on a) Al‐BEA and b) Sn‐BEA (0.2 M ^13^CH_3_OH, CH_3_CN, 298 K, 19.5 h (Al‐BEA) or 14 h (Sn‐BEA) after initial contact between the liquid solution and the zeolite). Blue dotted lines show the single‐pulse spectra for ^13^CH_3_OH in the absence of a catalyst (average of 68 scans). CP spectra are an average of 4368 scans (Al‐BEA) and 2440 scans (Sn‐BEA). Contact time for the CP spectra was 2 ms.

**Figure 4:**
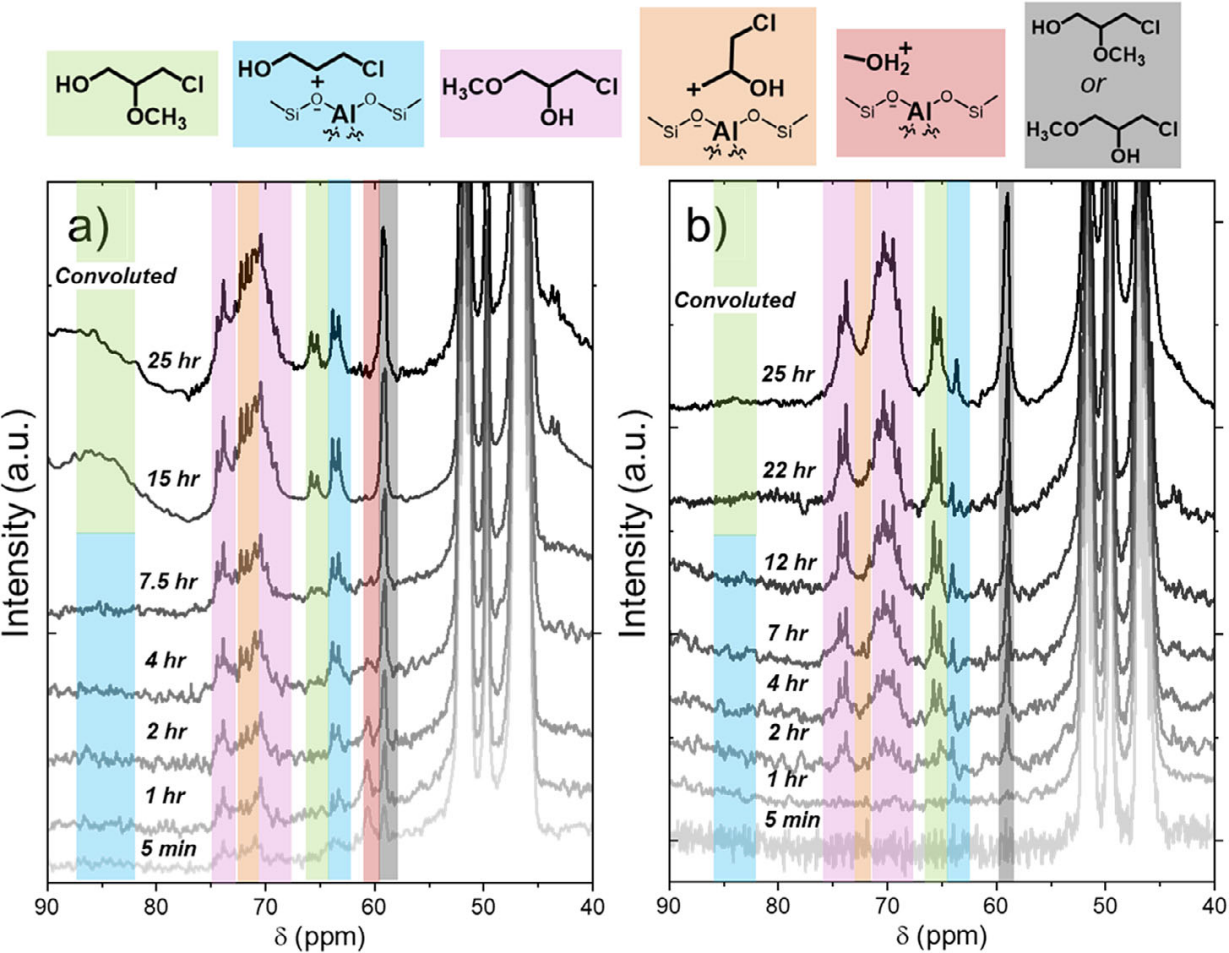
^13^C NMR single‐pulse spectra during ring‐opening of C_3_H_5_ClO with CH_3_OH over a) Al‐BEA and b) Sn‐BEA, where reaction was initiated by adding ^13^C_3_H_5_ClO to the NMR rotor after pre‐mixing all other components (0.2 M ^13^C_3_H_5_ClO, 0.2 M ^13^CH_3_OH, CH_3_CN, 298 K). Spectra are an average of scans, ranging from 16 scans (5 min time points) to 10,000 scans (last time points). See Section S12.1 for discussion of peak assignments for ring‐opening products and the carbocation intermediate species.

Figure 3 presents ^13^C NMR cross‐polarization spectra of ^13^CH_3_OH in contact with Al‐BEA and Sn‐BEA that show speciation among different forms of adsorbed methanol (0.2 M ^13^CH_3_OH, CH_3_CN, 298 K, 12 h). Figure 3a shows that ^13^CH_3_OH adsorbed to Al‐BEA gives peaks at 49.9, 51.7, and 60.6 ppm, while Sn‐BEA shows features at 49.7 and 51.6 ppm. The peaks at 49.7–49.9 ppm (green) align with previous proposals for CH_3_OH physisorbed to siloxane (Si─O─Si) surfaces^[^ ^34^ ^]^ and bound at Brønsted acid sites.^[^ ^34^, ^35^ ^]^ DFT calculations show that CH_3_OH coordination to Si─O─Si is highly unfavorable (bond length = 4.478 Å), and CH_3_OH significantly prefers to coordinate to Si─OH features (bond length = 1.714 Å) with a predicted peak shift of 49.7 ppm (Table S9, entry 15). The DFT‐predicted peak shifts (Table S9, entries 7 and 13) for intact CH_3_OH bound at Brønsted acid sites (49.6 ppm) and Sn─OH open sites (47.9 ppm) also agree well with this peak shift, suggesting that the 49.7–49.9 ppm feature may originate from a combination of CH_3_OH bound at acid sites and Si─OH features in Al‐ and Sn‐BEA. Several previous studies attribute peaks from 51–59 ppm for surface‐bound methoxy (─OCH_3_) groups in Brønsted acid zeolites formed from CH_3_OH,^[^ ^34^, ^35^, ^36^, ^37^ ^]^ while a study identifies a peak at 51 ppm as strongly bound, intact CH_3_OH at Brønsted acid sites.^[^ ^35^ ^]^ DFT calculations (Table S9, entries 9–10) predict respective peak shifts of 54.6 and 55.2 ppm for ─OCH_3_ groups bound at Brønsted acid sites and Sn─OH open sites. However, introducing a CH_3_OH adsorbed at an Si─OH function adjacent to the acid sites (Table S9, entries 11–12) shifts the predicted peaks for ─OCH_3_ at the acid site upfield (53.8 for Sn‐BEA, 52.0 for Al‐BEA). The peak for the adjacent CH_3_OH also shifts downfield significantly to a value of 52.3 ppm. These predicted shifts fall closer to the observed features at 51.6–51.7 ppm, indicating that these peaks may originate from a combination of ─OCH_3_ at acid sites accompanied by CH_3_OH coordinated at nearby surface sites. We tentatively assign the features at 51.6–51.7 ppm (yellow) as a mixture of ─OCH_3_ groups bound at acid sites in Al‐ and Sn‐BEA and surface‐bound CH_3_OH. Lastly, the peak at 60.6 ppm in Al‐BEA aligns closely with a measured shift for protonated CH_3_OH within a superacid (SO_2_–SbF_5_) solvent (61 ppm)^[^ ^38^ ^]^ but also resides near reported values for dimethyl ether (60–66 ppm)^[^ ^35^, ^39^ ^]^ that can form from bimolecular CH_3_OH dehydration. Dimethyl ether formation typically proceeds at temperatures over 423 K in Brønsted acid zeolites and thus seems improbable at ambient conditions as used for these spectra. Furthermore, DFT‐NMR calculations predict a peak shift of 57.5 ppm for DME solvated in CH_3_OH (Table S8), shifts of 51.4 and 52.4 ppm for DME adsorbed at Brønsted acid sites, and shifts of 57.0 and 57.1 ppm for DME adsorbed at Si─OH within Al‐BEA (Table S9, entry 24). These peak shifts all appear upfield of the observed peak at 60.6 ppm, further supporting that DME likely does not form over Al‐BEA. Lastly, the peak at 60.6 ppm disappears during the ring‐opening reaction (see Figure 4a below), indicating that this feature likely originates from an intermediate rather than a product molecule like DME. DFT predicts a peak shift of 68 ppm for protonated, liquid‐phase CH_3_OH (Table S8) and 50 ppm for protonated CH_3_OH at Brønsted acid sites (Table S9, entry 18). While these shifts do not align with the observed peak, we exclude DME based on the above evidence and tentatively assign the feature at 60.6 ppm (purple) to protonated CH_3_OH at Brønsted acid sites in Al‐BEA.

Rates of C_3_H_5_ClO ring opening normalized by catalyst mass upon purely siliceous *BEA materials fall 15 times lower than for Al‐BEA and Sn‐BEA catalysts, which suggests that CH_3_OH associated with the siloxane regions does not participate in catalysis.^[^ ^29^ ^]^ Protonated CH_3_OH acts as a weaker nucleophile than uncharged CH_3_OH, making protonated CH_3_OH an unlikely reactive nucleophile over Al‐BEA as well. Consequently, CH_3_OH bound to Brønsted acid and Sn‐BEA sites likely represents the reactive surface intermediate for the ring‐opening of C_3_H_5_ClO at a high ratio of [CH_3_OH] to [C_3_H_5_ClO] (i.e., surfaces saturated with CH_3_OH‐derived residues) over Al‐BEA and Sn‐BEA. The active bound CH_3_OH species may exist as intact CH_3_OH or dissociated ─OCH_3_ groups. The presence of the CH_3_OH‐derived features at 51.6–51.7 ppm, together with rates that increase in proportion to [C_3_H_5_ClO] and depend weakly on [CH_3_OH] (Table S5), suggests that both catalysts may ring‐open C_3_H_5_ClO by an S_N_2 reaction pathway at these conditions.

Lastly, the CH_3_CN co‐solvent does not appear to compete for active sites despite its strong polarity. The cross‐polarization spectra for the experiments in Figures 2 and 3 do not show a peak at ∼118 ppm corresponding to the C bound to N in CH_3_CN, nor do the spectra show broadening of the peak at ∼1 ppm from the methyl C in CH_3_CN (Figure S27). Taking these observations, together with the DFT prediction for the methyl carbon to shift 10–11 ppm upfield from ∼1 ppm when bound to the acid sites of Al‐ and Sn‐BEA (entries 26 and 27 of Table S9), indicates that CH_3_CN molecules do not coordinate to the catalyst surface.

The next section presents in situ NMR spectra obtained as a function of time during the ring‐opening reaction, which provide quantitative connections between instantaneous formation rates of each product and changes in the coverage of relevant intermediates. Examination of these spectra provides direct evidence for the involvement of surface species in specific reaction pathways.

### Analyzing Growth of 13C NMR Intermediate and Product Features for C3H5ClO Ring‐Opening

**Figure 5:**
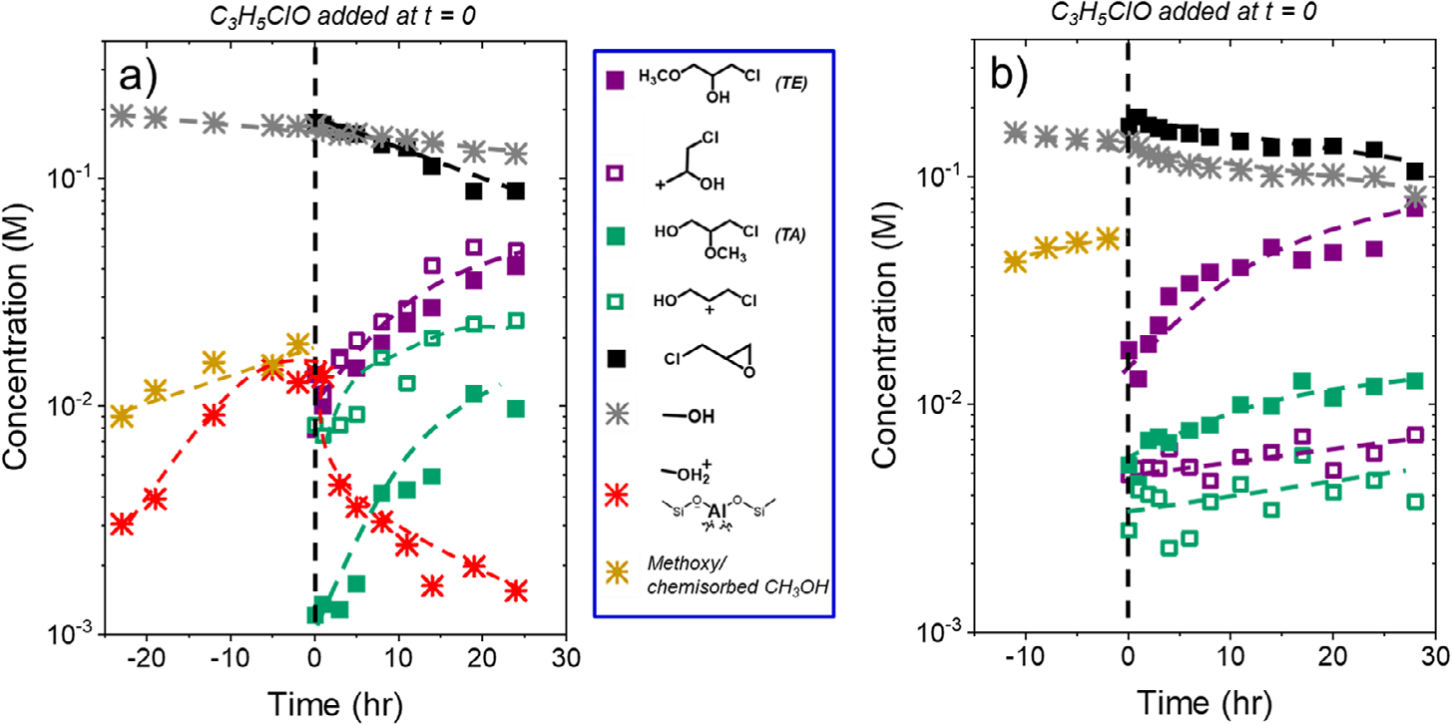
Concentration‐time profiles derived from ^13^C NMR single‐pulse spectra (Figure 4) during ring‐opening of C_3_H_5_ClO with CH_3_OH over a) Al‐BEA and b) Sn‐BEA, where reaction was initiated by adding ^13^C_3_H_5_ClO to the NMR rotor after pre‐mixing all other components (0.2 M ^13^C_3_H_5_ClO, 0.2 M ^13^CH_3_OH, CH_3_CN, 298 K).

**Figure 6:**
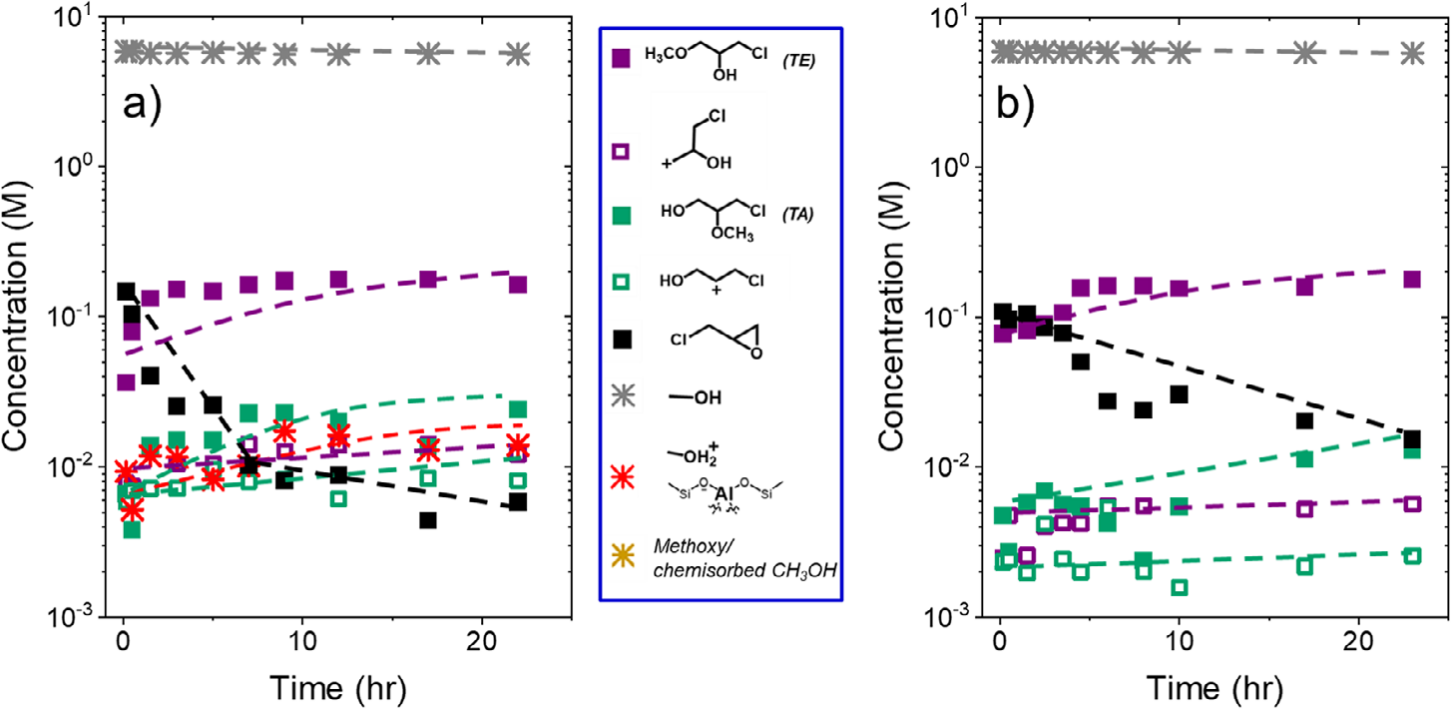
Concentration‐time profiles derived from ^13^C NMR single‐pulse spectra (Figure S28) during ring‐opening of C_3_H_5_ClO with CH_3_OH over a) Al‐BEA and b) Sn‐BEA, where reaction was initiated by adding all components to the rotor at once (0.2 M ^13^C_3_H_5_ClO, 6 M CH_3_OH, CH_3_CN, 298 K).

Figure 4 presents ^13^C NMR SP spectra collected as a function of time during the reactions among ^13^C_3_H_5_ClO and ^13^CH_3_OH at low [CH_3_OH] (0.2 M CH_3_OH) over Al‐BEA (Figure 4a) and Sn‐BEA (Figure 4b). Comparison of experimentally observed and DFT‐calculated peak shifts for liquid‐phase and surface species (see Section S12.1) supports the assignment of peak features as shown with the highlighted regions on the plots. The reactions were initiated by adding ^13^C_3_H_5_ClO to a rotor that contained a slurry of ^13^CH_3_OH, CH_3_CN, and the catalyst. These sequences directly show the evolution of peaks that reflect both primary and secondary C_3_H_5_ClO‐derived carbocations and concomitant formation of the terminal ether and terminal alcohol products with time, despite the convolution of a subset of the spectral features (Section S12.1). In particular, the increasing intensity of the feature for the methoxy groups within the products (∼ 59 ppm) demonstrates the accumulation of ring‐opening products within the NMR rotor. While Figure 4 shows that both products form readily at low [CH_3_OH] over Al‐BEA and Sn‐BEA, the different ratios of carbocations and products on the surface shown by both the SP spectra and CP spectra (see Figure S30) provide evidence that different reaction mechanisms may dominate over the catalysts at CH_3_CN‐rich conditions. The formation rates for the terminal ether become much greater than for the terminal alcohol over both Al‐BEA and Sn‐BEA upon increasing the value of [CH_3_OH] from 0.2 M CH_3_OH (Figure 4) to 6 M CH_3_OH (Figure S28). The secondary carbocation and terminal alcohol features appear at low intensities over time over Al‐BEA at 6 M CH_3_OH (Figure S28a), while neither species appears in noticeable quantities over Sn‐BEA (Figure S28b). The CP spectra confirm the absence of significant quantities of surface‐bound carbocations during the reaction (Figure S29). The similar patterns of peak growth over Al‐BEA and Sn‐BEA in Figure S28 give evidence that the materials may catalyze ring‐opening through similar reaction pathways in CH_3_OH‐rich conditions.

Figure 5 presents changes in concentrations of reactants, products, and surface intermediates derived from the ^13^C NMR SP spectra in Figure 4 (0.2 M CH_3_OH), which provides greater mechanistic insight. The spectra reveal the growth of activated CH_3_OH species on the surface before adding C_3_H_5_ClO, with protonated CH_3_OH forming over Al‐BEA (Figure 5a) and chemisorbed CH_3_OH appearing over both Al‐BEA and Sn‐BEA (Figure 5b). Measured rates from Figure 5 (and Figure 6 below) fall 10–25 times below those measured from batch kinetics (Figure 1), likely due to mass transfer limitations or product inhibition effects within the NMR rotor caused by the rotor spinning mechanism and a much higher ratio of catalyst to liquid as compared to the batch kinetic experiments (250 g_catalyst_ L_liquid_^−1^ versus 1 g L^−1^) (see Section 12.6). Nevertheless, the product distribution matches well between the batch reaction and NMR measurements, with differences in product formation rate ratio (*β*) of less than 25% at all comparable conditions (Section S12.6), which implies that mass transfer limitations or product inhibition may decrease product formation rates but does not affect the intrinsic reaction mechanism nor the impact of local ratios of reactant concentrations on the relative rates of S_N_1 and S_N_2 pathways. After adding C_3_H_5_ClO to initiate the reaction, the concentration of C_3_H_5_ClO steadily decreases while the concentrations of the ring‐opened carbocations and products increase. While the epoxide features prevent tracking the concentration of the chemisorbed CH_3_OH species (gold points) after adding the epoxide, the concentration of protonated CH_3_OH decreases over time in Al‐BEA. The decrease in CH_3_OH‐derived surface intermediate concentration with time indicates these species react with C_3_H_5_ClO to form ring‐opening products or desorb to allow C_3_H_5_ClO to coordinate to the surface. C_3_H_5_ClO adsorbs much more strongly to the surface than CH_3_OH (Table S7), suggesting that CH_3_OH readily desorbs to accommodate C_3_H_5_ClO adsorption. Nevertheless, these CH_3_OH‐derived species also likely react with liquid‐phase C_3_H_5_ClO to form ring‐opened products, with the chemisorbed CH_3_OH species (intact CH_3_OH or dissociated ─OCH_3_ groups) acting as the primary reactive intermediate derived from CH_3_OH during ring‐opening (vide supra). Interestingly, the concentrations of TE and the primary ring‐opened carbocation increase simultaneously over Al‐BEA (Figure 5b), while TA concentrations do not increase significantly until the secondary ring‐opened carbocation begins to accumulate on the surface. This distinction may simply indicate that the more stable secondary carbocation reacts less readily with CH_3_OH‐derived species than primary carbocations in an S_N_1 pathway. Alternatively, the more rapid initial accumulation of TE may suggest that TE formation does not require the formation of the primary carbocation as a prerequisite (S_N_2 pathway). In contrast, TA and TE appear at greater concentrations than the carbocations at all conditions in Sn‐BEA (Figure 5b), which may suggest that either product can form without the formation of a carbocation intermediate. The carbocations may also react more readily to form the ring‐opened products over Sn‐BEA, preventing significant accumulation of the carbocations on the surface.

**Figure 7:**
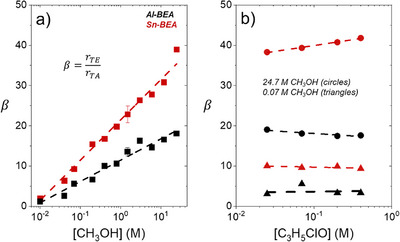
Values of *β* for C_3_H_5_ClO ring‐opening with CH_3_OH as a function of a) CH_3_OH concentration (0.07 M C_3_H_5_ClO, CH_3_CN, 308 K) and b) C_3_H_5_ClO concentration (triangles–0.07 M CH_3_OH, circles–24.7 M CH_3_OH, CH_3_CN, 308 K) over Al‐ (black) and Sn‐BEA (red).

Figure 6 demonstrates that TE forms rapidly at early times at 6 M CH_3_OH, then increases in concentration gradually over the remainder of the experiment after initiating the reaction by adding all components to the rotor simultaneously. TA shows concentrations one order of magnitude less than TE in both reactions, with a greater preference for TE in Sn‐BEA (consistent with Figure 1 (vide supra) and Figure 7 (vide infra)). While the secondary carbocation appears at more similar concentrations to TA, the concentrations of both products exceed the concentrations of their respective carbocations throughout the reaction. The formation of both products may primarily proceed through an S_N_2 pathway through CH_3_OH‐derived intermediate species over both catalysts at these conditions, where ring‐opened carbocations do not form before product formation. CH_3_OH‐derived intermediates should dominate the surface at 6 M CH_3_OH, consistent with the rate orders from Figure 1.

Overall, the growth patterns of reactants, intermediates, and products presented in these NMR spectra provide strong evidence for the mechanistic origins of regioselectivity differences for C_3_H_5_ClO ring‐opening over both Al‐BEA and Sn‐BEA. The following section describes how the changes in prevailing surface intermediates and the competition between S_N_1 and S_N_2 pathways lead to significant changes in reaction regioselectivity values as [CH_3_OH] changes.

### Rationalizing Regioselectivity Trends and Mechanistic Proposals for C_3_H_5_ClO Ring‐Opening

The ratio of the formation rates of the terminal ether to terminal alcohol (*β*) from Figure 1 depends on the concentration of liquid‐phase reactive species, adsorbed intermediate, and rate constants for the product‐forming steps:

(3)

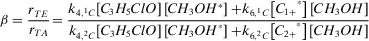




Figure 7 reveals that *β* values for C_3_H_5_ClO ring‐opening increase significantly with rising [CH_3_OH], yet these values remain largely constant with changes in [C_3_H_5_ClO]. In the absence of CH_3_CN co‐solvent (24.7 M CH_3_OH), Sn‐BEA and Al‐BEA show *β* values of 40 and 18, respectively. These values align with previously achieved regioselectivities for C_3_H_5_ClO ring‐opening with CH_3_OH without a co‐solvent over Al‐ and Sn‐BEA zeolites.^[^ ^13^ ^]^ Values of *β* decrease steadily with decreasing [CH_3_OH] (i.e., a larger fraction of CH_3_CN co‐solvent) and give very similar *β* values for Sn‐BEA (2.1) and Al‐BEA (1.2) at the lowest [CH_3_OH] studied (0.01 M CH_3_OH). These trends may indicate that the regioselectivity of C_3_H_5_ClO ring‐opening responds to selective solvation of transition states for the distinct regioisomers or that the regioisomers form through distinct reaction mechanisms.

The values of *β* from batch kinetics align closely with the magnitude and trend of *β* with [CH_3_OH] calculated from the NMR experiments (see Tables S9 and S10). The NMR spectra described in the previous sections give evidence that the prevailing reaction mechanism changes with reactant concentrations, which likely drives regioselectivity differences. The value of *β* from the NMR measurements increases with [CH_3_OH], providing evidence that the S_N_2 reaction through a CH_3_OH‐derived intermediate (CH_3_OH*) (step 4 in Scheme 1) significantly favors TE formation. Thus, the rate constant to form TA through the S_N_2 path ($k_{4{,}^{2}C}$) falls well below the values of the other rate constants in Equation 3, and more specifically, the rate of step 6_2C_ ($r_{6{,}^{2}C}$) surpasses that of step 4_2C_ ($r_{4{,}^{2}C}$) so the form simplifies under most conditions:

(4)

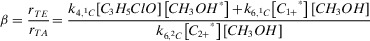




We reason that values for $k_{4{,}^{1}C}$ far exceed those for $k_{4{,}^{2}C}$ (i.e., $k_{4{,}^{1}C}$>> $k_{4{,}^{2}C}$) due to the steric hindrance of nucleophilic attack at the secondary epoxide carbon and the decreased electrophilicity of that second carbon due to the electron‐withdrawing effect of the Cl atom. Values for $k_{4{,}^{2}C}$ also likely fall well below those for $k_{6{,}^{1}C}$ and $k_{6{,}^{2}C}$ because the ring‐opened carbocations formed (step 6) possess greater electrophilicity than the intact epoxide (step 4), which decreases the barrier for nucleophilic attack by CH_3_OH.

Substituting in terms for [*CH*_3_ OH*], [*C*_1 +_ *], and [*C*_2 +_ *] then restating terms (see Section S8 for full derivation) yields:

(5)

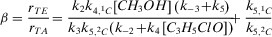




Equation 5 predicts a linear dependence of *β* on [*CH*_3_ *OH*] and agrees with trends evident in Figure 7a. However, this relationship also indicates that values of β may decrease with a sublinear dependence on [*C*_3_ *H*_5_ *ClO*]. Comparison between Figure 7b and Equation 5 indicates that CH_3_OH adsorption must approach quasi‐equilibrium in comparison to pathways that consume adsorbed CH_3_OH species (i.e., *k*_−2_ >> *k*_4_[*C*_3_ *H*_5_ *ClO*]) to explain the weak dependence of β on [*C*_3_ *H*_5_ *ClO*].

Common proposals for S_N_1 and S_N_2 pathways from organic chemistry suggest that the reaction regioselectivity for these pathways depends on different factors. First, the regioselectivity of S_N_1 reaction pathways depends primarily on the ring‐opened carbocation that forms.^[^ ^13^, ^23^, ^40^ ^]^ The S_N_1 pathway for C_3_H_5_ClO ring‐opening (Scheme 1) likely dominates at low [CH_3_OH]:[C_3_H_5_ClO] ratios, in which C_3_H_5_ClO can saturate the Brønsted acid sites in Al‐BEA and Lewis acid sites in Sn‐BEA. Figure 4 indicates that the carbocations responsible for TE and TA formation through the S_N_1 pathway exist at similar coverages, which aligns with β values near unity at the lowest [CH_3_OH] studied (Figure 7). More specifically, β values at the lowest [CH_3_OH] (2.1 for Sn‐BEA, 1.2 for Al‐BEA) closely resemble the ratios of primary to secondary ring‐opened carbocations in CH_3_CN (2.4 for Sn‐BEA, 1.4 for Al‐BEA). The rate expression reflects a constant β value in the limit where [CH_3_OH]:[C_3_H_5_ClO] approaches zero in Equation 5:

(6)

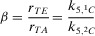




These trends further support that the S_N_1 pathway dominates when C_3_H_5_ClO‐derived species saturate active sites, where the carbocation ratio drives differences in regioselectivity through changes in $k_{5{,}^{1}C}$ and $k_{5{,}^{2}C}$.

In contrast to S_N_1 pathways, the regioselectivity of S_N_2 pathways depends on the steric hindrance during the rate‐determining nucleophilic attack step.^[^ ^13^, ^23^, ^40^ ^]^ An S_N_2 reaction should, therefore, prefer to form TE because of the greater steric hindrance associated with the nucleophilic attack at the interior epoxide carbon to form TA. The S_N_2 pathway through CH_3_OH‐derived intermediates in Scheme 1 likely prevails at greater [CH_3_OH]:[C_3_H_5_ClO] ratios. The increasing β values as [CH_3_OH] increases (Figure 7) may thus originate from an increased concentration of activated CH_3_OH species on the active sites. The data from the previous sections support that adding greater fractions of CH_3_OH increases the quantity of CH_3_OH adsorbed and near the catalyst surface, which promotes the S_N_2 pathway that favors TE formation. In the limit when [CH_3_OH]:[C_3_H_5_ClO] approaches infinity, we assume that the $k_{4{,}^{2}C}$ term in Equation 3 become relevant. The *k*_4 _terms dominate and the rate expression again reveals a constant value of β:
(7)

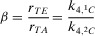




Equations 6 and 7 demonstrate that β values depend only on the rate constants for ring‐opened carbocation or product formation in purely S_N_1 or S_N_2 pathways, respectively. The changes in β with [CH_3_OH] therefore reflect a shifting preference between these two competing pathways.

To summarize, the findings discussed here demonstrate the value of in situ, solid‐state ^13^C NMR spectroscopy in unveiling reaction mechanisms over heterogeneous catalysts. Interestingly, regioselectivities for C_3_H_5_ClO ring‐opening depend much more strongly on the reaction environment than for 1,2‐epoxybutane ring‐opening, as demonstrated in our previous work.^[^ ^28^ ^]^ The electron‐donating groups in epoxides like 1,2‐epoxybutane and styrene oxide stabilize carbocations and likely make the S_N_1 pathway dominant over a wider range of reaction conditions than with C_3_H_5_ClO, which may reduce the effect of the epoxide to nucleophile ratio on regioselectivities. Therefore, the reaction environment likely plays a more significant role in S_N_1/S_N_2 switching and regioselectivities with epoxides containing electron‐withdrawing groups like C_3_H_5_ClO (see Section S12.7 for full discussion).

More broadly, the consequences of the reaction environment have been demonstrated for homogeneous glycosylation reactions, where the choice of alcohol acceptor and solvent influences product distribution by altering the prevalence of S_N_1 and S_N_2 pathways.^[^ ^41^ ^]^ Previous computational work demonstrated that these effects apply across hundreds of nucleophilic substitution reactions and that the inclusion of charged functional groups or external fields shifts homogeneous reactions between S_N_1 and S_N_2 pathways to control product selectivity.^[^ ^42^ ^]^ The mechanism carries clear implications for reaction regioselectivity in confined environments, as zeolites stabilize certain carbocations and charged (or neutral) transition states through shape selectivity, which alters the product distribution for reactions including alkane isomerization and cracking,^[^ ^43^ ^]^ biomass transformations,^[^ ^44^ ^]^ and alkylation of aromatics.^[^ ^45^, ^46^ ^]^ A greater understanding of the role of the reaction environment on catalytic mechanisms enables the design of processes with improved rates and selectivities towards desired products.

## Conclusion

The combination of in situ, solid‐state ^13^C NMR spectra and rate measurements provides molecular insight into the form of the surface intermediates, the corresponding reaction pathways for liquid‐phase C_3_H_5_ClO ring‐opening in zeolites, and explains why product regioselectivities depend sensitively on reactant concentrations. Reaction regioselectivity shifts from nearly an even product distribution between the terminal ether and terminal alcohol products at low [CH_3_OH] to nearly singular formation of the terminal ether product (>95%) in the absence of the CH_3_CN co‐solvent. In situ ^13^C‐NMR spectroscopy experiments demonstrate that these regioselectivity differences originate from changes in the prevailing reaction mechanism and dominant surface intermediates as [CH_3_OH] changes. C_3_H_5_ClO activates to form ring‐opened carbocations on the surface of both Al‐BEA and Sn‐BEA, which provides evidence that C_3_H_5_ClO ring‐opening proceeds by an S_N_1 pathway in the limit of low nucleophile concentrations. CH_3_OH forms activated methoxy species over both Al‐BEA and Sn‐BEA, supporting that each zeolite catalyzes an S_N_2 reaction pathway at higher nucleophile concentrations. Time‐resolved NMR measurements reveal the concomitant evolution of surface intermediates and liquid‐phase products, which provide direct evidence for the identity of the intermediates responsible for the S_N_1 and S_N_2 reaction pathways. Together, kinetics and spectroscopy indicate that C_3_H_5_ClO ring‐opening proceeds through an S_N_1 pathway with a ring‐opened surface intermediate derived from C_3_H_5_ClO at low [CH_3_OH], and an S_N_2 pathway mediated by an activated CH_3_OH intermediate at higher [CH_3_OH]. Regioselectivity for the S_N_1 pathway depends primarily on the preference to form the primary or secondary carbocation of the ring‐opened complex, and the similarities among rate constants lead to nearly equimolar quantities among terminal ether and alcohol products at low [CH_3_OH]. In contrast, significantly destabilizing intermolecular forces arise during the attack of an activated CH_3_OH by C_3_H_5_ClO at higher [CH_3_OH] (S_N_2 pathway), leading to a predominant formation of the terminal ether. In situ ^13^C‐NMR spectra reveal that Bronsted acid sites of Al‐BEA favor the formation of the ring‐opened carbocations from C_3_H_5_ClO more readily than Sn‐BEA (a material with both Lewis and Bronsted acid character), which leads to greater selectivities to the terminal alcohol over Al‐BEA due to the prevalence of the S_N_1 mechanism.

These observations demonstrate that liquid‐phase C_3_H_5_ClO ring‐opening with CH_3_OH may proceed through S_N_1 or S_N_2 pathways over Lewis and Brønsted acid zeolites, where the catalyst identity and composition of reactants and solvents dictate the dominant pathway. The competition between these mechanisms drives regioselectivity differences and allows for much lower selectivities to the terminal ether (i.e., greater selectivities to the terminal alcohol) than predicted from organic chemistry intuition. The active site environment determines if carbocation stability or steric hindrance governs the regioselectivity of nucleophilic substitution reactions, thereby providing opportunities to manipulate product distribution towards desired species. The use of in situ solid‐state ^13^C NMR spectroscopy provides crucial knowledge of the formation, structure, and reactivity of organic surface species that govern rates and selectivities for catalysis within micropores of zeolites. Here, we provide spectroscopic evidence for epoxide ring‐opening mechanisms not previously established over zeolites, including the observation of stable ring‐opened carbocations and the likely presence of both S_N_1 and S_N_2 mechanisms over Lewis and Bronsted acid zeolites. While demonstrated here for epoxide ring‐opening, manipulation of the reaction environment significantly influences product distributions at solid–liquid interfaces in thermocatalysis, electrocatalysis, photocatalysis, and more, thus yielding broader insight needed for the design of more productive catalytic processes.

## Supporting Information

Supporting information includes the materials and methods section, X‐ray diffraction, diffuse‐reflectance UV–vis, ex situ Raman spectroscopy, transmission total‐reflectance FT‐infrared spectroscopy, ex situ solid‐state ^27^Al NMR spectroscopy, GC sensitivity factors, hot filtrations, Madon–Boudart tests, 1,2‐diphenyl‐1,2‐ethylenediamine site titrations, supplemental epoxide ring‐opening kinetics, rate expression derivation, excess enthalpies and free energies, activation barriers and free energy diagrams, isothermal titration calorimetry and adsorption enthalpies, experimental and predicted ^13^C NMR peak shifts, supplemental ^13^C NMR spectra, batch and ^13^C NMR kinetics comparisons, regioselectivity trend comparisons between C_3_H_5_ClO and C_4_H_8_O, uncertainties and limitations of ^13^C NMR experiments.

## Conflict of Interests

The authors declare no conflict of interest.

## Supporting information



Supporting Information

## Data Availability

The data that support the findings of this study are available in the supplementary material of this article.

## References

[1] S. T. Oyama , in Mechanisms in Homogeneous and Heterogeneous Epoxidation Catalysis, Elsevier, Amsterdam, the Netherlands, 2008, pp. 3–99.

[2] Vol. WO 2020/176277 A1, 2020;

[3] Vol. US 2020/0231738 A1, 2020.

[4] Vol. US *9*,822,087 B2, *Dow Global Technologies LLC* 2017.

[5] X. Li , L. M. Candela , M. A. Leipa , D. W. Leyshon , Vol. EP3362427B1, 2021.

[6] J. H. Teles , 2005.

[7] 2021.

[8] 2021.

[9] J. McMurry , Organic Chemistry, Cengage Learning, Boston, MA, USA, 2016.

[10] M. B. Smith , March's Advanced Organic Chemistry: Reactions, Mechanisms, and Structure, John Wiley & Sons, Hoboken, NJ, USA, 2020.

[11] Y. M. Terblans , M. Huyser , D. A. Young , M. J. Green , Can. J. Chem. 2006, 84, 859–866. 10.1139/v06-086.

[12] D. B. G. Williams , M. Lawton , Org. Biomol. Chem. 2005, 3, 3269. 10.1039/b508924g. 16132088

[13] N. Deshpande , A. Parulkar , R. Joshi , B. Diep , A. Kulkarni , N. A. Brunelli , J. Catal. 2019, 370, 46–54. 10.1016/j.jcat.2018.11.038.

[14] H. Ogawa , Y. Miyamoto , T. Fujigaki , T. Chihara , Catal. Lett. 1996, 40, 253–255. 10.1007/BF00815291.

[15] P. Manjunathan , V. Prasanna , G. V. Shanbhag , Sci. Rep. 2021, 11, 15718. 34344963 10.1038/s41598-021-95089-1 PMC8333069

[16] T. Weil , M. Kotke , C. M. Kleiner , P. R. Schreiner , Org. Lett. 2008, 10, 1513–1516. 10.1021/ol800149y. 18366220

[17] L. H. Wee , F. Bonino , C. Lamberti , S. Bordiga , J. A. Martens , Green Chem. 2014, 16, 1351–1357. 10.1039/C3GC41988F.

[18] M. Marelli , F. Zaccheria , N. Ravasio , E. Pitzalis , Y. Didi , A. Galarneau , N. Scotti , C. Evangelisti , Catalysts 2023, 13, 341. 10.3390/catal13020341.

[19] R.‐X. Zhou , Y.‐J. Wang , X.‐M. Zheng , Catal. Lett. 2005, 100, 201–203.

[20] Q. Yue , V. Kasneryk , M. Mazur , S. Abdi , Y. Zhou , P. S. Wheatley , R. E. Morris , J. Čejka , M. Shamzhy , M. Opanasenko , J. Mater. Chem. A 2024, 12, 802–812. 10.1039/D3TA06161B. PMC10763919 38178865

[21] Y. X. Zhou , Y. Z. Chen , Y. Hu , G. Huang , S. H. Yu , H. L. Jiang , Chemistry–A European Journal 2014, 20, 14976–14980. 10.1002/chem.201404104. 25291973

[22] H. Ogawa , M. Mori , Reaction Kinetics and Catalysis Letters 1995, 56, 377–382. 10.1007/BF02076043.

[23] R.‐E. Parker , N. Isaacs , Chem. Rev. 1959, 59, 737–799. 10.1021/cr50028a006.

[24] F. Moschona , I. Savvopoulou , M. Tsitopoulou , D. Tataraki , G. Rassias , Catalysts 2020, 10, 1117. 10.3390/catal10101117.

[25] S. V. Narina , A. Sudalai , Tetrahedron 2007, 63, 3026–3030. 10.1016/j.tet.2007.01.057.

[26] M. N. Bhagat , C. K. Bennett , G.‐F. Chang , Y. Zhu , A. Raghuraman , M. E. Belowich , S. T. Nguyen , L. J. Broadbelt , J. M. Notestein , ACS Catal. 2019, 9, 9663–9670. 10.1021/acscatal.9b03089.

[27] C. K. Bennett , M. N. Bhagat , Y. Zhu , Y. Yu , A. Raghuraman , M. E. Belowich , S. T. Nguyen , J. M. Notestein , L. J. Broadbelt , ACS Catal. 2019, 9, 11589–11602. 10.1021/acscatal.9b02607.

[28] D. S. Potts , J. K. Komar , H. Locht , D. W. Flaherty , ACS Catal. 2023, 13, 14928–14944. 10.1021/acscatal.3c04103.

[29] D. S. Potts , J. K. Komar , M. A. Jacobson , H. Locht , D. W. Flaherty , JACS Au, 2024, 4, 3501–3518. 39328744 10.1021/jacsau.4c00398 PMC11423312

[30] F. A. Carey , Organic Chemistry, McGraw‐Hill, New York City, NY, USA, 1996.

[31] B. D. Montejo‐Valencia , J. L. Salcedo‐Pérez , M. C. Curet‐Arana , J. Phys. Chem. C 2016, 120, 2176–2186. 10.1021/acs.jpcc.5b09815.

[32] L. Ford , R. Burrows , N. Raffaele , N. A. Brunelli , J. Phys. Chem. C 2025, 129, 7787–7794. 10.1021/acs.jpcc.5c00968.

[33] M. J. Duer , Solid State NMR Spectroscopy: Principles and Applications, John Wiley & Sons, Oxford, UK, 2008.

[34] I. I. Ivanova , Y. G. Kolyagin , Chem. Soc. Rev. 2010, 39, 5018. 10.1039/c0cs00011f. 21038049

[35] W. Wang , M. Seiler , M. Hunger , J. Phys. Chem. B 2001, 105, 12553–12558. 10.1021/jp0129784.

[36] Y. Jiang , M. Hunger , W. Wang , J. Am. Chem. Soc. 2006, 128, 11679–11692. 10.1021/ja061018y. 16939294

[37] M. Anderson , Journal of the Chemical Society Faraday Transactions 1998, 94, 2851–2856.

[38] G. A. Olah , A. M. White , J. Am. Chem. Soc. 1969, 91, 5801–5810. 10.1021/ja01049a017.

[39] M. Zhang , S. Xu , J. Li , Y. Wei , Y. Gong , Y. Chu , A. Zheng , J. Wang , W. Zhang , X. Wu , J. Catal. 2016, 335, 47–57. 10.1016/j.jcat.2015.12.007.

[40] T. Hansen , P. Vermeeren , A. Haim , M. J. van Dorp , J. D. Codée , F. M. Bickelhaupt , T. A. Hamlin , Eur. J. Org. Chem. 2020, 2020, 3822–3828. 10.1002/ejoc.202000590.

[41] Y. Fu , L. Bernasconi , P. Liu , J. Am. Chem. Soc. 2021, 143, 1577–1589. 10.1021/jacs.0c12096. 33439656 PMC8162065

[42] L.‐J. Yu , M. L. Coote , The Journal of Physical Chemistry A 2019, 123, 582–589. 10.1021/acs.jpca.8b11579. 30566349

[43] G. Noh , Z. Shi , S. I. Zones , E. Iglesia , J. Catal. 2018, 368, 389–410. 10.1016/j.jcat.2018.03.033.

[44] J. Jae , G. A. Tompsett , A. J. Foster , K. D. Hammond , S. M. Auerbach , R. F. Lobo , G. W. Huber , J. Catal. 2011, 279, 257–268. 10.1016/j.jcat.2011.01.019.

[45] C. Li , P. Ferri , C. Paris , M. Moliner , M. Boronat , A. Corma , J. Am. Chem. Soc. 2021, 143, 10718–10726. 10.1021/jacs.1c04818. 34240857 PMC8529870

[46] S. Ezenwa , H. Montalvo‐Castro , A. J. Hoffman , H. Locht , J. Attebery , D.‐Y. Jan , M. Schmithorst , B. Chmelka , D. Hibbitts , R. Gounder , J.G Am. Chem. Soc. 2024, 146, 10666–10678. 10.1021/jacs.4c00373. 38573868

