# Supplementary material for: In Situ NMR and Kinetics Reveal Origins of Regioselectivity Differences for Epichlorohydrin Ring‐Opening in Lewis and Brønsted Acid Zeolites
Source: Angew Chem Int Ed Engl. 2025 Oct 27;64(50):e202511944. doi: 10.1002/anie.202511944 (PMC12684356; doi:10.1002/anie.202511944)
Supplement: Supplementary file 1 — Supporting Information [file ANIE-64-e202511944-s001.docx]

*In Situ* NMR and Kinetics Reveal Origins of Regioselectivity Differences for Epichlorohydrin Ring-Opening in Lewis and Brønsted Acid Zeolites

David S. Potts,^1^ Huston Locht,^1,2^ Sungmin Kim,^3^ Johannes Lercher,^3,4^ Jian Zhi Hu,^3,5*^

David W. Flaherty^1,2*^

^1^Department of Chemical and Biomolecular Engineering

University of Illinois Urbana-Champaign, Urbana, Illinois 61801 (USA)

^2^School of Chemical and Biomolecular Engineering

Georgia Institute of Technology, Atlanta, Georgia 30332 (USA)

^3^Institute for Integrated Catalysis

Pacific Northwest National Laboratory, Richland, Washington 99352 (USA)

^4^Department of Chemistry and Catalysis Research Center, TU München, Lichtenbergstrasse 4, 85748 Garching, Germany

^5^The Gene & Linda Voiland School of Chemical Engineering and Bioengineering, Washington State University, Pullman, WA 99164, United States

Corresponding author e-mails: [jianzhi.hu@pnnl.gov](mailto:jianzhi.hu@pnnl.gov), [dflaherty3@gatech.edu](mailto:dflaherty3@gatech.edu)

**S1. Methods**

**S1.1 Catalyst Synthesis**

The M-BEA materials (M = Al, Sn) were prepared by post-synthetic modification of a commercial Al-BEA material (TOSOH, lot no. 94HA6X02Y; Si/Al = 20). For Al-BEA, the parent Al-BEA material was treated in dilute HNO_3_ (1 M) for ~3 h to partially remove Al atoms from the framework by forming soluble (AlNO_3_)_3_ complexes. After this, the resulting Al-BEA material was loaded into a quartz boat and placed in a three-zone furnace (Applied Test Systems, 3210), which was heated to 823 K (5 K min^-1^) in flowing air (100 cm^3^ min^-1^; Airgas, Ultra Zero grade) and held at 823 K for 6 h to remove organic residues. The heat treatment resulted in a white powder Al-BEA sample.

For Sn-BEA, the commercial Al-BEA was first treated in concentrated, refluxing HNO_3_ (Macron Chemicals, 68 – 70 wt%, 20 cm^3^ g^-1^) at 433 K for ~24 h to fully remove Al atoms from the *BEA framework. The HNO_3_ treatment was repeated three times to give a thoroughly dealuminated material denoted as Si-BEA (Si/Al > 3500 from ICP-OES, *vide infra*). Between acid treatments, the catalyst sample was washed with deionized H_2_O (18.2 MΩ cm, Elga Purelab Flex 2, 50 cm^3^ g_zeolite_^−1^) and recovered with vacuum filtration. After this, the Si-BEA material was subjected to the same heat treatment described above. Sn-BEA was then prepared from the recovered Si-BEA material through a dry impregnation and calcination treatment, adapted from previously published procedures.^1-4^ Si-BEA was first dried at 393 K overnight to remove residual H_2_O molecules. Then, Si-BEA (6 g) was ground with dimethyl tin dichloride (400 mg; (CH_3_)_2_SnCl_2_, Alfa Aesar, ≥98.5%) for 600 s in the fume hood using a mortar and pestle. *Note: Take precaution when using (CH_3_)_2_SnCl_2_, as it is highly toxic if inhaled or swallowed.* The solid mixture was transferred to a quartz boat, loaded into the three-zone furnace, and heated to 823 K (2 K min^-1^) in flowing Ar (100 cm^3^ min^-1^; Airgas, Ultra High Purity) and held at 823 K for 4 h. After this, the gas flow was switched from Ar to air, and the solid was held at 823 K for another 4 h. The pre-treatment in an inert environment (i.e., Ar) has previously been shown to reduce the formation of tin oxide clusters within zeolite materials.^3-4^ This solid-state incorporation treatment yielded a white powder Sn-BEA sample.

**S1.2 Catalyst Characterization**

The metal contents of M-BEA were calculated with inductively coupled plasma optical emission spectroscopy (ICP-OES, PerkinElmer, Optima 8300) measurements carried out by staff scientists at the Microanalysis Laboratory at the University of Illinois. Sn-BEA contains negligible quantities of Al (Si/Al ~ 4000), while Al-BEA shows Si/Al ~ 75. Reported turnover rates are normalized with the metal loadings determined by ICP-OES. Metal loadings and Si/Al ratios for each zeolite are shown in Table S1.

The dispersity of the M atoms in M-BEA was examined with diffuse reflectance UV-visible (DRUV-vis) spectroscopy. Magnesium oxide (MgO, Sigma-Aldrich, 99.9995%) was used as a background for measurements. M-BEA samples were mixed and finely ground with MgO at a MgO:M-BEA mass ratio of 10:1. The reflectance spectra were obtained with a UV-vis spectrophotometer (Varian, Cary 5G) under ambient conditions. The band gap energies (Table S1) were determined by extrapolating the linear portion of the Tauc plots ([$F(R)\cdot h{v]}^{1/2}$) to the horizontal axis to determine the minimum energy of photons absorbed (eV) (Figure S1). Sn-BEA shows a greater band gap than SnO_2_, supporting that the Sn atoms reside at tetrahedral positions within the framework at dispersed locations. Al-BEA and Al_2_O_3_ show negligible reflectance features, likely because the band gap of each material exceeds the scan range of the spectrophotometer. Nevertheless, the unfavorable nature of Al-O-Al pairs (Loewenstein's rule^5^) and low Si/Al ratio of Al-BEA (~75) supports that the Al atoms remain dispersed throughout the *BEA framework.

The binding structure of Al atoms in Al-BEA was probed with *ex-situ* ^27^Al NMR spectroscopy (Bruker AVIII 400, 10 kHz spin speed). Figure S2 presents the ^27^Al NMR spectra for Al-BEA with and without C_5_H_5_N present. These spectra provide insight into the structure of Al atoms with and without adventitious H_2_O present, supporting that ~99% of the Al atoms exist in tetrahedral coordination within Al-BEA.

The coordination of metal atoms within M-BEA was further studied using Raman spectroscopy. *Ex situ* Raman spectra were acquired at ambient conditions on pressed catalyst pellets with a Raman spectrometer (Renishaw, InVia) equipped with a 532 nm laser. The accumulation time was 20 s per scan, and each Raman spectra was calculated by averaging 10 scans. The power density at the sample was approximately 2 mW μm^-2^ (Gentec-EO, PRONTO-SI). The Raman spectra for M-BEA (Figure S3) do not show features in the regions where Al_2_O_3_ (640, 751 cm^-1^)^6-8^ and SnO_2_ (632, 773 cm^-1^)^9-11^ show strong features, which indicates the absence of M-O-M bonds. Together with the high band gap energies (Table S1), these spectra support that the M atoms predominantly reside in tetrahedral positions within *BEA.

The crystallinities of M-BEA were probed with an X-ray diffractometer (Bruker, D8 Advance) with Cu Kα radiation under ambient conditions. The M-BEA powders were loaded into a polypropylene sample holder for the measurements. The diffractograms of M-BEA (Figure S4a) match previously reported diffraction patterns for *BEA.^12^ Furthermore, the patterns for M-BEA do not possess strong features in the characteristic peak locations for the respective metal oxides (Figure S4b), which further indicates negligible quantities of the Sn and Al exist in their native oxide form. Metal oxide samples were purchased commercially and used as received: γ-Al_2_O_3_ (US Research Nanomaterials, Inc., 20 nm, 99%) and SnO_2_ (Sigma-Aldrich, EMPLURA®, ≥99.0%).

The relative density of (SiOH)*_x_* groups within M-BEA was determined with infrared spectroscopy. A background spectrum was first taken with an empty transmission cell configured with CaF_2_ windows. The cell was loaded into a Fourier-transform infrared spectrometer (Bruker, Vertex 70) equipped with a liquid N_2_-cooled HgCdTe detector, as described previously.^13^ The cell was connected to a temperature controller and gas manifold, then heated to 573 K (~5 K min^-1^) and held for 2 h in flowing Ar (50 cm^3^ min^-1^; Airgas, Ultra High Purity) to desorb volatile compounds and H_2_O. The background spectrum was then collected at 573 K (128 scans, 4 cm^-1^). After taking the background spectra, M-BEA was pressed into a pellet and loaded into the transmission cell, where the spectra (Figure S5) were obtained in the same manner as the background measurement. The vibrational features at 1800-2100 cm^-1^ and 3300-3750 cm^-1^ represent the Si-O-Si overtones^14^ of the *BEA framework and *v*(O-H) of (SiOH)*_x_* groups,^15-16^ respectively. The broad features from 3300-3700 cm^-1^ arise from SiOH defects containing multiple hydroxyl moieties within (SiOH)*_x_* groups. The sharper feature at ~3750 cm^-1^ represents isolated SiOH defects that do not interact with other SiOH.^15-16^ The ratio of the areas for *v*(O-H) and *v*(Si-O-Si) at 1865 and 2000 cm^-1^ gives a relative measure of SiOH density defined as $\Phi_{IR}$ (Equation S1) in each M-BEA. The isolated SiOH feature is excluded from $A_{\nu\left( O-H \right)}$ in Equation S1. The *v*(O-H) region was deconvoluted into multiple peaks (see Figure S6), and the isolated SiOH peak area was subtracted from the combined area of the *v*(O-H) region. Al-BEA shows an additional feature at ~3600 cm^-1^ that represents Brønsted acidic SiOH sites associated with the presence of framework Al atoms.^17-18^ We do not exclude this feature from *v*(O-H), so the $A_{\nu\left( O-H \right)}$ term encompasses both Brønsted acid SiOH and (SiOH)*_4_* formed by Al removal.

$\Phi_{IR}=\frac{A_{\nu\left( O-H \right)}}{A_{\nu\left( Si-O-Si \right)}}$ (S1)

The values of $\Phi_{IR}$ (Table S1) and calculated estimates of (SiOH)_x_ (unit cell)^-1^ (Section S3.6) indicate that each material contains a similar density of (SiOH)_x_. For Al-BEA, the SiOH features from Brønsted acid sites constitute ~9% of the total (SiOH)_x_ within the material.

The acid site character of M-BEA was characterized by infrared spectra of adsorbed pyridine (C_5_H_5_N, Sigma-Aldrich, >99%) and deuterated acetonitrile (CD_3_CN, Cambridge Isotope Laboratories, 99.8% D atom). The catalysts were pelletized, loaded into the spectrometer, and pre-treated at 573 K, as described above. The cell was then cooled to either 393 K (C_5_H_5_N) or 303 K (CD_3_CN). A background spectrum was taken before flowing the adsorbates. C_5_H_5_N or CD_3_CN were introduced with a syringe pump (KD Scientific, Legato) and vaporized into a stream of flowing Ar (20 cm^3^ min^-1^) within the heated gas-transfer lines. Reported spectra were collected after flowing at a certain vapor pressure for ~1 h, where the absorbance features had reached a steady state. The spectra confirm the presence of Brønsted acid sites in Al-BEA and Lewis acid sites in Sn-BEA. A detailed analysis of the spectra is provided in Section S3.7.

Collectively, the characterization of M-BEA supports that each material possesses a crystalline *BEA framework containing dispersed, tetrahedrally incorporated metal atoms and similar densities of (SiOH)*_x_*.

**S1.3 Epoxide Ring-Opening Turnover Rate Measurements**

Turnover rates for epichlorohydrin (C_3_H_5_ClO, Sigma-Aldrich, ≥99.0%) ring-opening with methanol (CH_3_OH, Sigma-Aldrich, ≥99.9%) were measured in three-necked round-bottom flasks with magnetic stirring (700 rpm). The reactants were combined with acetonitrile (CH_3_CN, Fisher Chemical, HPLC Grade) as co-solvent and benzene (Sigma-Aldrich, thiophene-free, >99%) as an internal standard. The flasks were connected to reflux condensers to prevent evaporative losses and were submerged in a temperature-controlled bath containing either H_2_O (298 – 318 K) or silicone oil (Sigma-Aldrich, viscosity 100 cSt) (323 – 328 K) on a hot plate (Corning, PC-420D). The reaction mixture was stirred at reaction temperature for 0.5 h before taking an aliquot (~0.5 cm^3^) to determine the initial composition of the mixture, including the concentrations of C_3_H_5_ClO and ring-opening products. M-BEA (~25-70 mg) was then added to initiate the reaction, and aliquots were taken as a function of time with a syringe equipped with a polypropylene filter (Tisch Scientific, 0.05 μm) to separate the sample solution from the catalyst. The ring-opening reaction yields two possible products: a terminal ether and terminal alcohol. The concentrations of C_3_H_5_ClO, benzene, and the ring-opening products were quantified with a gas chromatograph (GC) (Agilent, 6850) equipped with a liquid autosampler, flame ionization detector, and polysiloxane column (HP-1, Agilent, 19091Z-115E). Elution times and sensitivity factors were determined using commercially purchased samples of epichlorohydrin, CH_3_OH, benzene, and the terminal ether ring-opening product (3-chloro-1-methoxy-2-propanol, Sigma-Aldrich, ≥97.0%) (Section S4). The terminal alcohol ring-opening product was not available for purchase, so this compound was assumed to have an identical sensitivity factor as the terminal ether, based on the similar sensitivity factors for the products from 1,2-epoxybutane ring-opening.^19^

Turnover rates were measured with CH_3_CN as a co-solvent to allow for independent control of the concentrations of both C_3_H_5_ClO and CH_3_OH (i.e., [C_3_H_5_ClO], [CH_3_OH], where [*x*] denotes the concentration of species *x*). Additional measurements were obtained in neat CH_3_OH (24.7 M CH_3_OH). Initial turnover rate values were calculated by fitting turnover numbers (moles of product formed per moles of active metal) as a function of time to a second-order polynomial with the turnover number constrained to equal zero at time equal to zero, and subsequently, turnover rates were determined from the derivative at time equal to zero. Data points used in these analyses were acquired at differential conversion of the limiting reagent (<10%) and with an uncertainty of ~10%, as calculated from replicated experiments. The carbon balance closes within 90 – 110% for most measurements, although the error increases at the lowest conversions (<0.5%) where the carbon balance closes between 70 – 125%. Furthermore, the carbon selectivity to the desired ring-opening products exceeds 95% across all measurements. Two unknown peaks appear at GC retention times later than the ring-opening products (17.7 min, 18.2 min) (Figure S10). These peaks appear to be secondary products (e.g., oligomers from ring-opening products reacting with a second equivalent of epichlorohydrin). However, standards were not available to confirm these species, and these species account for < 5% of all products formed.

Functional experimental tests were conducted to assess the impact of mass transfer processes on rate measurements and to exclude the possibility that transport artifacts prevent the measurement of intrinsic kinetic behavior and corrupt comparisons between catalysts.^20-21^ The conditions used for rate measurements avoid internal and external mass transfer constraints because turnover rates do not depend on the densities of metal atoms within the *BEA crystallites across all compositions studied (Figure S11 in Section S5). These measurements demonstrate that all M-BEA materials satisfy the Madon-Boudart criterion^20^ and mass transfer processes do not influence apparent rates. In addition, the total rate of product formation exhibits combinations of first- and zero-order dependencies on the concentrations of C_3_H_5_ClO and CH_3_OH at limiting conditions.^22^ A mass-transfer limited material would show a sub-linear dependence on reactant concentrations because the mean concentration of the reactants throughout the pores would not depend linearly on the fluid phase reactant concentrations for a zero-order reactant.

The stability of the M-BEA materials was tested through XRD analysis before and after exposing the catalyst to the ring-opening reaction mixture for 24 hours. Figure S12 shows that the fresh and spent M-BEA catalysts show nearly identical diffraction patterns, supporting that the catalyst does not deactivate or experience structural changes during C_3_H_5_ClO ring-opening with CH_3_OH. Hot filtration experiments utilizing 1,2-epoxybutane (C_4_H_8_O) ring-opening^19^ also provide evidence that the M-BEA materials are stable under ring-opening reaction conditions. Figure S13 shows that the product concentrations change negligibly after filtering out the solid catalyst but continue to increase in the presence of the catalyst. This supports that the catalytic active sites do not leach from the *BEA framework during ring-opening. Furthermore, active site titration experiments using 1,2-diphenyl-1,2-ethylenediamine (DPED) demonstrate that greater than 90% of metal atoms are active for C_4_H_8_O ring-opening (Figure S14).^19, 23^ These experiments demonstrate the stability of the specific catalysts used in the investigation during the ring-opening of epoxides.

**S1.4 Liquid-Phase Adsorption Enthalpies for Reactants**

The heats released upon adsorption of C_3_H_5_ClO or CH_3_OH to catalytic active sites were measured with an isothermal titration calorimeter (ITC) (TA Instruments, NanoITC) equipped with sample and reference cells (0.5 cm^3^). A brief cleaning procedure was performed to prepare the sample and reference cells for each experiment. First, 500 cm^3^ of a cleaning solution (10 volume % detergent in DI H_2_O, 18.2 MΩ cm, Elga Purelab Flex 2) was flushed through the cell at room temperature. The sample cell was then rinsed with DI H_2_O (1000 cm^3^). The sample cell and reference cell were then filled with DI H_2_O (0.35 cm^3^), an ITC syringe was filled with DI H_2_O (0.05 cm^3^), and then the syringe was loaded on the calorimeter. A subsequent electrical calibration used sequential pulses to determine the heat released from a calibrated Pt resistor. Finally, DI H_2_O was injected into DI H_2_O to ensure the sample cell was clean enough for experiments. The cell was assumed to be clean when the H_2_O-H_2_O injection released negligible amounts of heat (± 3 μJ for each injection of 1 μL). The cleaning procedure was repeated until H_2_O-H_2_O injections satisfied this criterion.

In a typical experiment, a slurry of M-BEA (15-45 mg) in CH_3_CN was titrated by C_3_H_5_ClO or CH_3_OH (0.005 M) diluted in CH_3_CN. The mass of M-BEA in the sample cell was calculated by taking the difference between the mass added to the slurry and the mass remaining upon evaporation of the residual slurry after loading the cell. The titrations were carried out at 308 K with a stirring rate of 250 rpm. The sample cell was filled with the M-BEA slurry (0.35 cm^3^), and the reference cell was filled with CH_3_CN (0.35 cm^3^). The molar enthalpies of adsorption for C_3_H_5_ClO were calculated by averaging the integrated heats released upon adsorption of the titrant molecules to M-BEA sites from a 1 μL injection of titrant at low coverages (< 0.25 mol titrant (mol M)^-1^). The heat released per injection in this low coverage regime remains approximately constant for C_3_H_5_ClO, which suggests that the calculated molar enthalpies represent the isosteric adsorption enthalpies on each material.^24^ During titrations with CH_3_OH, the heat released per injection is much lower and continues to vary slightly with increasing titrant quantities. This indicates that the adsorption enthalpy for CH_3_OH varies with coverage, possibly due to solvation or clustering within pores. Section S11 presents ITC thermograms and related analysis.

**S1.5 *Operando* Solid-State ^13^C NMR Spectroscopy**

*Operando* ^13^C magic angle spinning (MAS) NMR experiments were carried out in a wide-bore 300 MHz NMR spectrometer (Varian, Inova) equipped with a 7.5 mm Vespel MAS NMR probe and a heating stack for variable temperature measurements. The corresponding Larmor frequencies for ^1^H and ^13^C measurements were 299.97 and 75.43 MHz, respectively. For both single pulse (SP) ^13^C and ^1^H-^13^C cross-polarization (CP) MAS NMR experiments, sample spinning rates ranging from 4400 to 5100 Hz were used. The SP experiments were acquired with a 45-degree angle pulse, an acquisition time of 100 ms, and a recycle delay of 10 s. For CP experiments, contact times ranging from 0.1 to 2 ms were used with a recycle delay time of 4 s. The reported spectra are an average of multiple scans, ranging from 20 scans to 3,000 scans for SP spectra and from 500 to 10,000 scans for CP spectra. The ^13^C NMR spectra were referenced to tetramethylsilane (TMS) set at 0 ppm, with adamantane as a second reference (downfield ^13^C peak at 38.48 ppm).

For the NMR experiments, M-BEA (70-80 mg) was loaded into an all-zirconia, MAS NMR rotor equipped with an O-ring and screw cap for sealing that was developed at Pacific Northwest National Lab (0.3 cm^3^ volume, 7.5 mm outside diameter).^25-26^ After loading the catalyst, a desired quantity of liquid (CH_3_OH, CH_3_CN, or a mixture of both) was injected into the rotor using a micro syringe. The rotor was sealed and transported to the NMR spectrometer, where the sealed rotor cap was opened and a desired amount of C_3_H_5_ClO was injected into the catalyst slurry (if necessary for the experiment). The probe tuning was then calibrated and the spinning rate was set (range of 4400 to 5100 Hz). The first NMR spectra can be acquired as short as a few minutes after loading the rotor into the spectrometer. However, the entire catalyst slurry was loaded in a separate laboratory from where the spectrometer was located for several initial experiments (Figures S28-29 and S33-S34). In those cases, the first spectrum was not collected until 0.3 – 0.5 hr after contacting the reactant solution and the M-BEA catalyst.

Chemical shifts for liquid-phase species were obtained by averaging values and errors obtained from ChemDraw (version 23.0.1) and the NMR Predictor software from ACD/Labs (v2024), which were cross-referenced with the reported shifts available in the literature. Chemical shifts for surface-bound species were predicted with the Amsterdam Density Functional package (ADF 2024).^27^ The ^13^C NMR calculations were performed based on the geometry-optimized structures at the same level of theory and with the same basis set to evaluate the chemical shielding for each atom. The calculations utilized the all-electron triple-ζ, 2-polarization function basis set with the Slater-type orbitals implemented in the ADF program.^28^ The geometries were optimized using the generalized gradient approximation with Grimme's third-generation dispersion correction applied to the Becke–Lee–Yang–Parr functional employed for geometry optimization.^29-31^ The DFT-calculated peak shifts for both liquid-phase and surface-bound species utilized the COSMO solvation model,^32^ with a single liquid-phase or adsorbed molecule embedded with a continuum solvent phase. As with the experimentally collected spectra, the calculated ^13^C NMR spectra were referenced to TMS at 0 ppm. Section S12.1 reports the chemical shifts for all liquid-phase and surface-bound species.

**S2. Inductive Effects in Epoxide Ring-Opening**


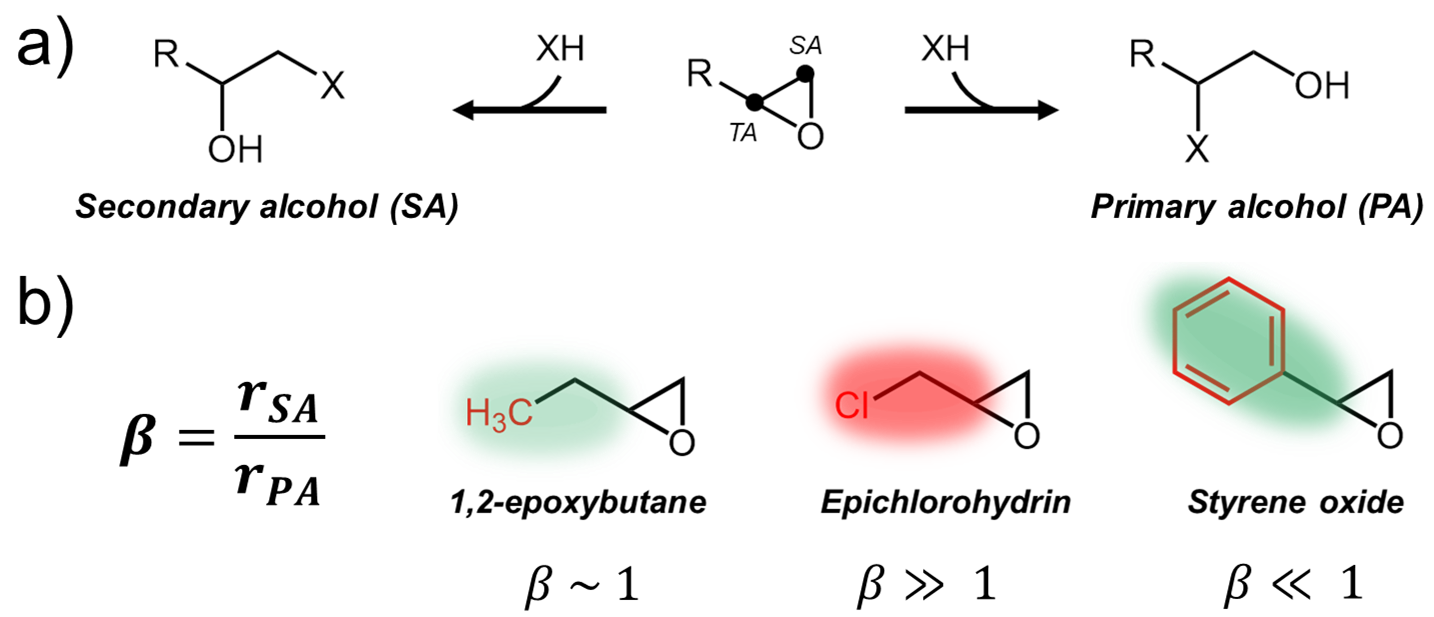


**Scheme S1**. a) Stoichiometric reaction for epoxide ring-opening with a nucleophile (XH), where either a secondary or primary alcohol product may form. b) Structures for 1,2-epoxybutane (a representative aliphatic epoxide with terminal methyl group), epichlorohydrin, and styrene oxide which illustrate an electron withdrawing effect (red shading) and electron donating effect (green shading), where a more intense shading indicates a stronger effect. These inductive effects significantly influence ratios of rates to form each isomer as quantified by values of  *β*.

Note that the terminal ether (TE) formed during epoxide ring-opening with alcohols is a secondary alcohol, while the terminal alcohol (TA) product is a primary alcohol.

**S3. Catalyst Characterization**

| Catalyst | M wt%^a^ | Si:M^a^ | Si:Al^a^ | Band Gap (eV)^b^ | $\Phi_{IR}$^c^ | Active Metal (%)^d^ |  |  |  |  |  |  |  |
| --- | --- | --- | --- | --- | --- | --- | --- | --- | --- | --- | --- | --- | --- |
| Al-BEA | 0.59 | 75 | 75 | -- | 1.56 | 97 ± 4 |  |  |  |  |  |  |  |
| Sn-BEA | 1.35 | 143 | 3950 | 5.2 | 1.51 | 93 ± 6 |  |  |  |  |  |  |  |
| **Table S1.** Characterization of the Chemical, Physical, and Electronic Properties of M-BEA.  ^a^Measured with ICP-OES. ^b^Extracted from leading edge of Tauc plot from DRUV-vis. ^c^Calculated from infrared transmission spectra  of dehydrated M-BEA samples. ^d^Measured from *in situ* 1,2-diphenylethylenediamine site titrations. | | | | | | | |  |  |  |  |  | 104 ± 7 |
|  | | | | | | | |  |  |  |  |  | 96 ± 2 |

c

Table S1 summarizes the key material properties of the M-BEA materials, the calculation methods of which are described in the characterization subsections below.

**S3.1 Diffuse Reflectance UV-Vis Spectra to Examine Dispersity of Active Sites**


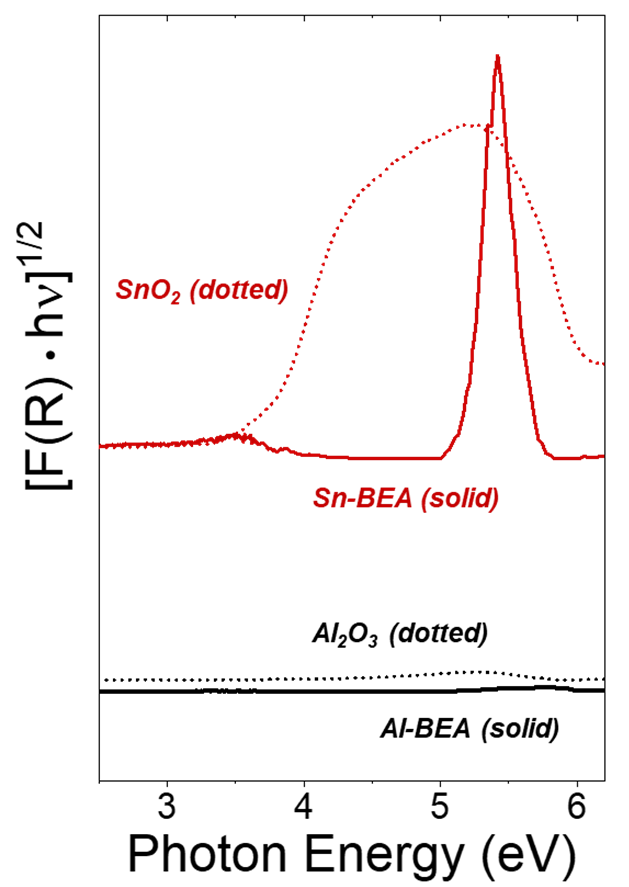


**Figure S1**. Tauc plots obtained with a UV-Vis spectrophotometer at ambient conditions for M-BEA (solid lines; Al ($-$), Zr ($-$)) and the respective metal oxides (dotted lines; γ-Al_2_O_3_ (**--**), SnO_2_ (**--**)). UV-Vis spectra were obtained from 200 to 800 nm. Tauc plots were normalized to the most intense features and are vertically offset for clarity.

The band gaps for each M-BEA and the respective metal oxides were obtained by extrapolating the linear absorbance region of the Tauc plot in Figure S1 to the baseline. The intersection of the baseline and linear region equals the band gap photon energy. Al-BEA and γ-Al_2_O_3_ show negligible features within the region of photon energies examined, consistent with reported band gaps greater than 7 eV for γ-Al_2_O_3_.^33-34^ Table S2 reveals that the band gaps for Sn-BEA is greater than the band gaps for the metal oxides, providing evidence that the metal atoms are well dispersed in the *BEA framework.

The photon energy *E* in electron volts (eV) was obtained from the wavelength as follows:

$$E=\frac{3*{10}^{8}\frac{m}{s}}{\lambda\left( nm \right)*\frac{1 m}{{10}^{9} nm}}*h*\left( 6.242*{10}^{18}\frac{eV}{J} \right)$$

where $\lambda$is the wavelength of the photon in nanometers, and *h* equals the Planck constant. The ordinate from Figure S1 was obtained from the ordinate of the raw spectra, the percent reflectance (% R). The Kubelka-Munk function (*F(R))* was calculated from % R:

$$F\left( R \right)=\frac{\left( 1-\frac{\%R}{100} \right)^{2}}{2*\left( \frac{\%R}{100} \right)}$$

F(R) was then multiplied by the photon energy, and taking the square root of the resulting quantity yielded the ordinate for Figure S1.

| Catalyst | M-BEA  Band Gap (eV) | Metal Oxide Band Gap (eV) |
| --- | --- | --- |
| Al-BEA | -- | -- |
| Sn-BEA | 5.2 | 3.9 |

**Table S2**. Calculated band gaps for M-BEA and metal oxide materials. The band gaps for Al-BEA and Al_2_O_3_ likely exceed the regime of wavelengths possible to measure on the UV-Vis spectrophotometer.


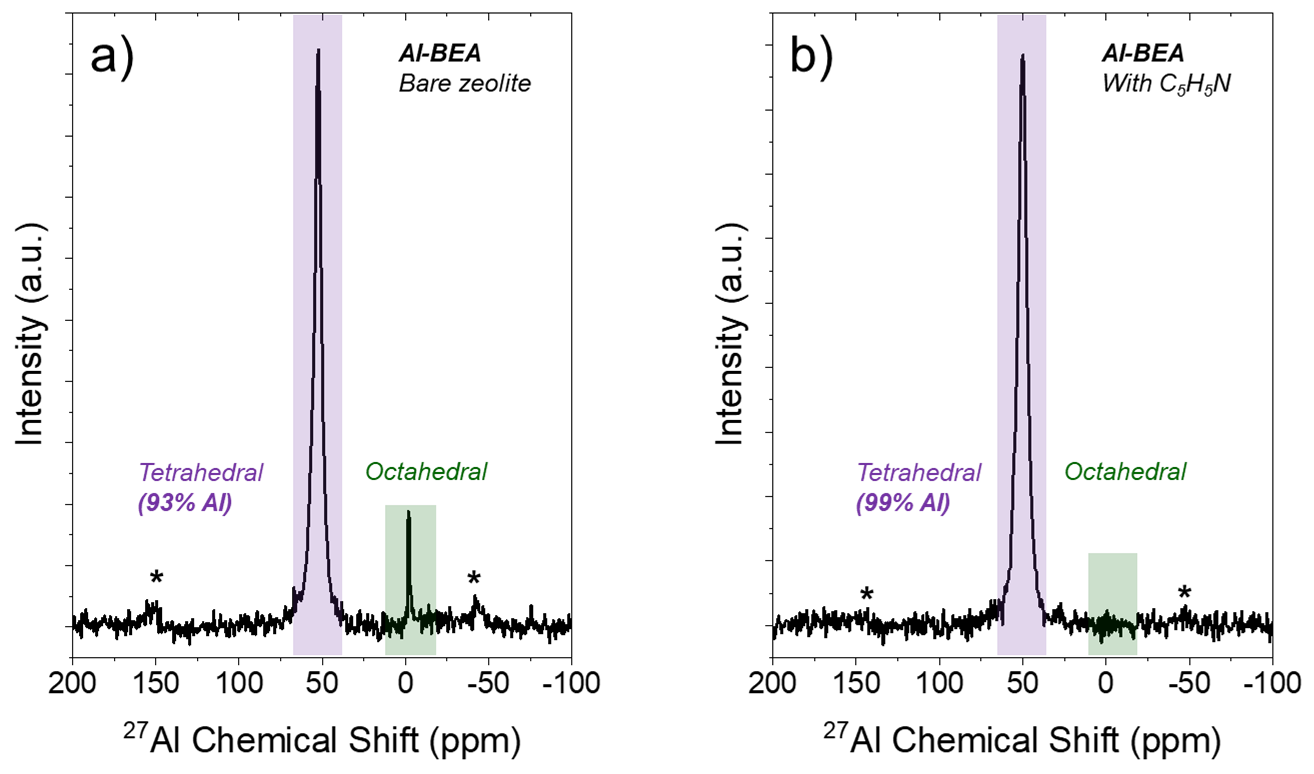
**S3.2 ^27^Al NMR Spectra to Elucidate Al Coordination in Al-BEA**

**Figure S2.** ^27^Al NMR spectra for a) bare Al-BEA and b) Al-BEA impregnated with C_5_H_5_N (298 K). The peaks representing tetrahedrally and octahedrally coordinated Al species are highlighted in purple and green, respectively. The calculated percentage of tetrahedral Al is shown in bold. Asterisks represent spinning side-band features.

Figure S2 presents the ^27^Al NMR spectra for Al-BEA in the presence and absence of liquid pyridine (C_5_H_5_N). The percentage of tetrahedral Al shown in the figure was calculated from the ratio of the tetrahedral to octahedral peak area features. Notably, the bare zeolite shows a greater percentage of octahedral Al than the materials impregnated with C_5_H_5_N. Liquid or vapor water (H_2_O) binds to tetrahedral Al atoms and gives rise to octahedral ^27^Al NMR features, which reflects the additional coordination of two H_2_O molecules.^35-37^ H_2_O adsorbed from the atmosphere likely contributes to the larger quantity of octahedral Al observed in the bare zeolite. Previous works found that loading hydrated Brønsted acid zeolites with ammonia (NH_3_) converts octahedral Al species back to tetrahedral Al sites,^37-39^ making the adsorption of a strong Brønsted base a reliable strategy to report octahedral Al content without interference from H_2_O

Motivated by the role of NH_3_ shown in previous studies, we impregnated the Al-BEA materials with liquid C_5_H_5_N to revert hydrated Al species to tetrahedral Al atoms. Figure S2 shows that the C_5_H_5_N-incorporated Al-BEA material shows lower quantities of octahedral Al (1%) than the bare materials (7%). The value for the C_5_H_5_N-incorporated Al-BEA aligns closely with the value of 3% obtained from active site titrations (Section S7, *vide infra*). Overall, the spectra in Figure S2 support that Al-BEA contains minor quantities of octahedral Al that do not convolute the kinetics reported in the main text**.**


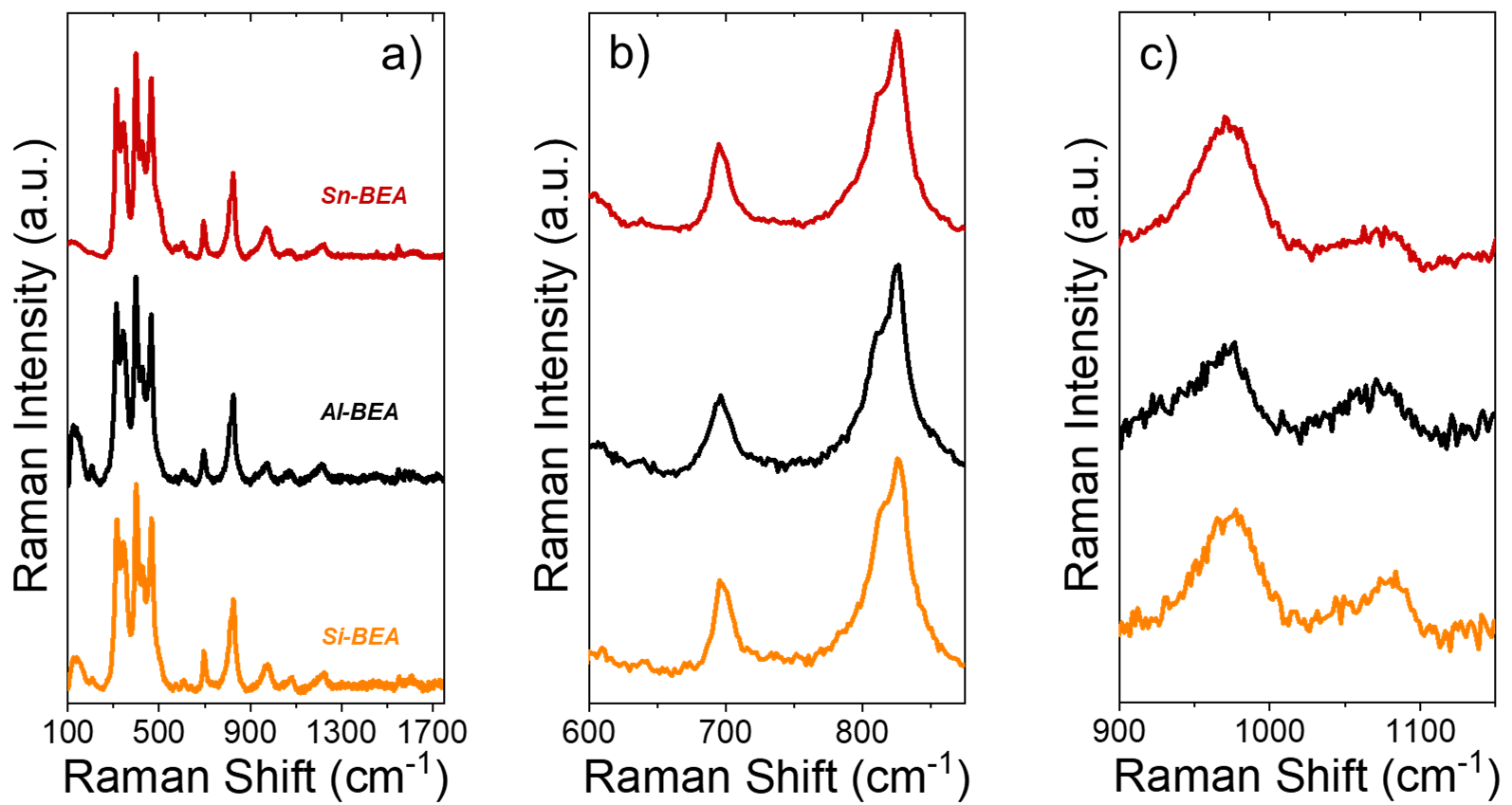
**S3.3 *Ex situ* Raman Spectra to Determine Metal Coordination in M-BEA**

**Figure S3.** *Ex situ* Raman spectra of M-BEA (Sn (red), Al (black), Si (orange)) at (a) the full range of Raman shifts examined, (b) magnified view of the range of 600-875 cm^-1^, and (c) magnified view of the 900-1150 cm^-1^ region. The spectra were taken at ambient conditions with a Raman spectrometer (Renishaw, InVia) equipped with a 532 nm laser that delivered a power density of ~2 mW μm^-2^. The spectra are an average of 10 scans with an exposure time of 20 s. Spectra are normalized by the maximum feature at ~400 cm^-1^ and are vertically offset for clarity.

Figure S3a reveals that each M-BEA and Si-BEA possess similar Raman spectra. The intense peaks between 250 and 550 cm^-1^ represent features of the *BEA framework: the peaks at 315 and 345 cm^-1^ represent six-membered rings, the peaks at 400 and 425 cm^-1^ represent five-membered rings, and the peak at 465 cm^-1^ represents four-membered rings characteristic of the *BEA framework.^40-41^ The peak at ~695 cm^-1^ has previously been reported on spectra of *BEA zeolites and may originate from the *BEA framework.^42-43^ The peak at ~820 cm^-1^ represents a symmetric skeletal mode of microporous silicates.^44-45^

Both M-BEA show a feature at 144 cm^-1^. The feature is also present in Si-BEA, suggesting the feature results from a mode inherent to the *BEA framework. Alternatively, the feature may be an artifact of the Raman filter used to suppress signal from Rayleigh scattering. Figure S3b zooms into a region in which Al_2_O_3_ (640, 751 cm^-1^)^6-8^ and SnO_2_ (632, 773 cm^-1^)^9-11^ have been reported to show Raman features. Both M-BEA show nearly identical spectra in this region, with no notable features where the respective metal oxides contain features. This supports that both M-BEA do not contain oligomeric metal oxide structures.

Figure S3c shows that each M-BEA possesses features at ~960 and ~1080 cm^-1^. The feature at ~960 cm^-1^ has been attributed to silanol defects.^46-48^ The peak at ~1080 cm^-1^ plausibly originates from the *BEA framework. Ti-O-Si features have been commonly reported in the literature at ~960 and ~1125 cm^-1^, respectively.^49-52^ However, similar features have not been well reported for Al- or Sn-containing zeolites. Therefore, the presence of each metal in the framework cannot be confirmed with Raman spectra but is indirectly supported by the absence of metal oxide features.

The Raman spectra in Figure S3 suggest that the Al and Sn atoms within M-BEA predominately reside at tetrahedral framework positions within the *BEA zeolite framework.

**S3.4 X-Ray Diffraction to Confirm Crystallinity of M-BEA**


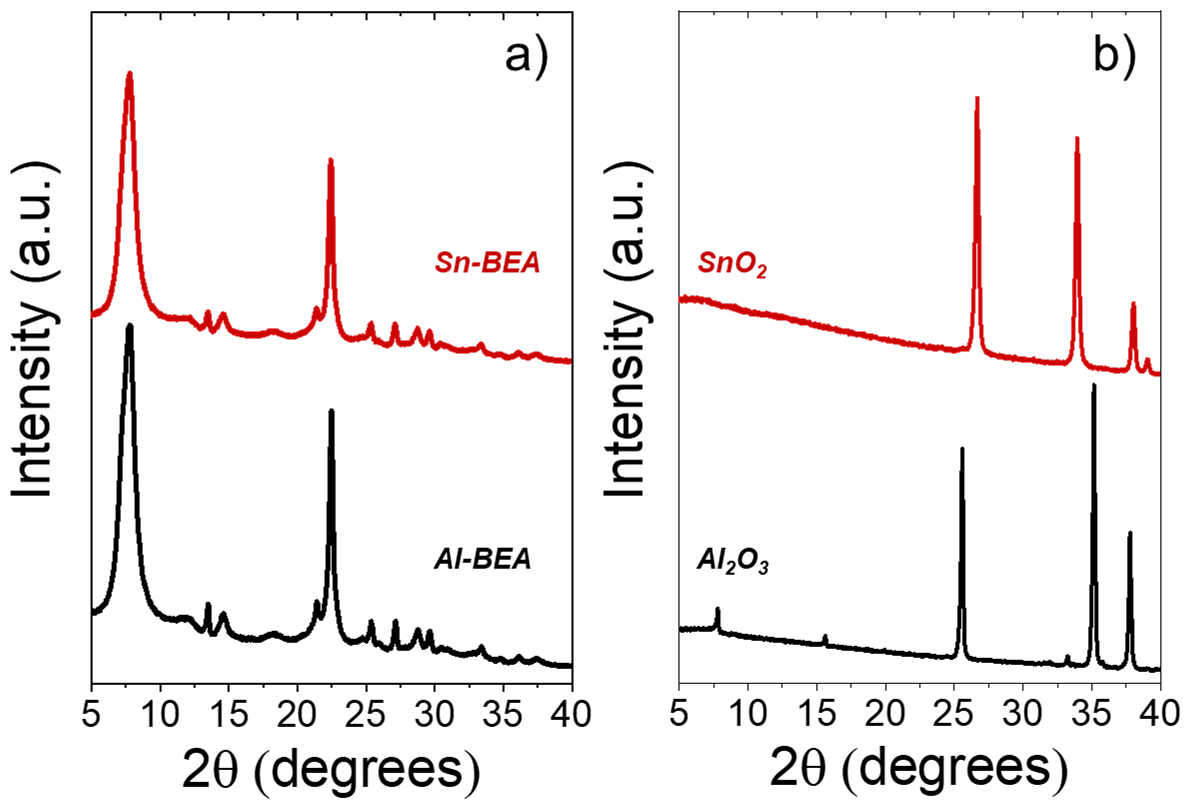


**Figure S4.** Powder X-ray diffractograms obtained with Cu Kα radiation (0.15418 nm) under ambient conditions for a) M-BEA (Al (black), Sn (red)) and b) the corresponding bulk metal oxides (γ-Al_2_O_3_ (black), SnO_2_ (red)). Spectra are vertically offset for clarity.

The crystallographic features for each M-BEA are representative of the *BEA framework.^12^ As displayed in Figure S4a, each M-BEA material shows weak features around 25.5, 27, 29, and 33 degrees. The broad feature at ~22.5 degrees suggests a small crystallite size^53^ and the presence of internal (SiOH)_x_ defects formed by the dealumination of the parent Al-BEA material (Si:Al = 20).

Figure S4b reveals that several of these peak locations coincide with strong features in the diffractograms of at least one of the metal oxides. However, the presence of weak features at these positions in all M-BEA suggests that the features are characteristic of the *BEA framework, rather than originating from the presence of metal oxide. Overall, the XRD patterns in Figure S4 support that each M-BEA possesses the crystalline *BEA framework, with minimal to no detectable metal oxide crystallites present.

**S3.5 Infrared Spectra to Measure Silanol Density of M-BEA**


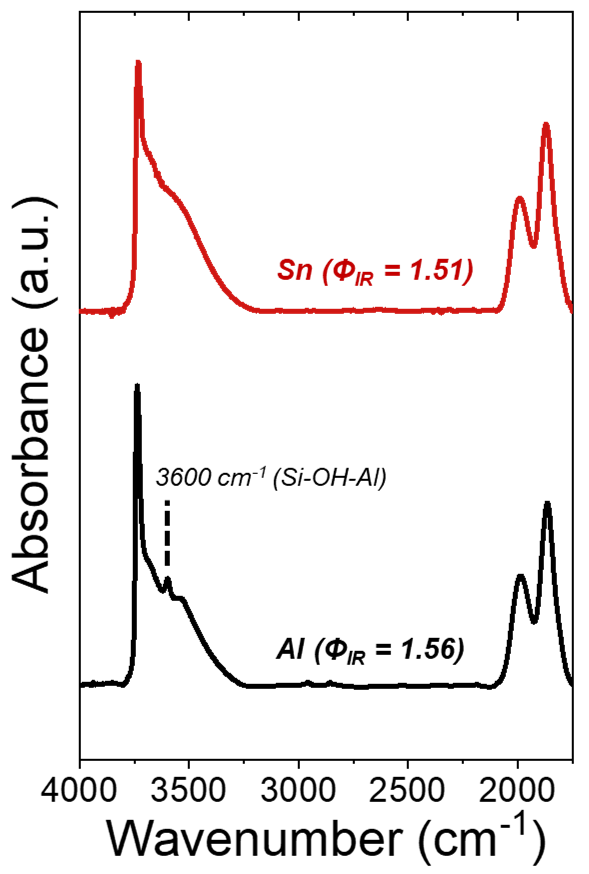


**Figure S5.** Infrared spectra of dehydrated samples of M-BEA (Sn (red), Al (black)). The samples were dehydrated under flowing Ar (101 kPa He, 50 cm^3^ min^-1^) at 573 K prior to measurement. Spectra are vertically offset for clarity.

The greater *v*(O-H) areas (3300-3750 cm^-1^) for each M-BEA-OH compared to M-BEA-F, with respect to the *v*(Si-O-Si) area (1800-2100 cm^-1^), shows that the M-BEA-OH materials possess a greater density of (SiOH)_x_ groups than the M-BEA-F materials. The Φ_IR_ values in Table S1 were calculated by peak fitting on Origin 2021 (OriginLab Corporation). The peak areas for *v*(O-H) and *v*(Si-O-Si) were calculated with Gaussian fits (example shown in Figure S6).

As shown in Equation S1 above, the equation to calculate Φ_IR_ is:

$\Phi_{IR}=\frac{A_{\nu\left( O-H \right)}}{A_{\nu\left( Si-O-Si \right)}}$

The red, purple, and orange peaks represent networked SiOH features which are summed to determine A_v(O-H)_. The green and brown peaks in the Si-O-Si region were summed together to get $A_{\nu\left( Si-O-Si \right)}$. The blue peak in *v*(O-H) represents isolated SiOH features, so these peak areas were excluded from the Φ_IR_ calculations.

**
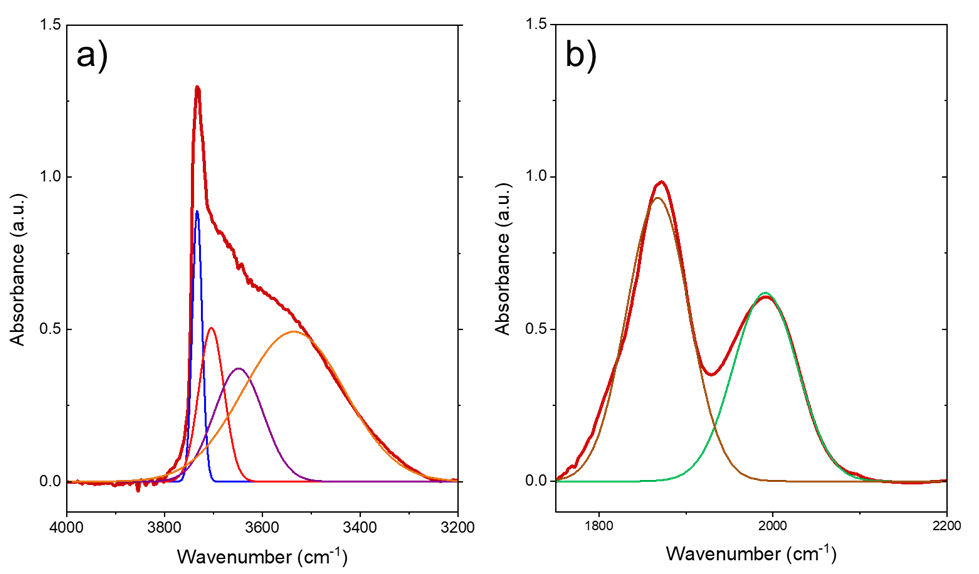
**

**Figure S6.** Fitted peaks for the (a) networked (red, purple, orange) and isolated (blue) Si-OH region and (b) Si-O-Si overtones at 1865 (brown) and 2000 cm^-1^ (green) for Sn-BEA.

**S3.6 Calculation of (SiOH)_x_ (unit cell)^-1^ for M-BEA**

The density of (SiOH)_x_ per unit cell was estimated based on the initial quantity of Al present in the commercial zeolite (Si:Al = 20). A *BEA unit cell contains 64 T atoms (i.e., Si, Al, or Sn), so the initial quantity of Al per unit cell equals 3.047:

$$Si+Al=64$$

$$20Al+Al=64$$

$$\boldsymbol{Al=3.047 per unit cell}$$

Assuming all Al atoms are removed in the thorough dealumination treatment for Sn-BEA, the dealumination produces 3.047 (SiOH)_x_ (unit cell)^-1^. The metal weight loading allows for the calculation of the Si:M ratio for each M-BEA:

$$Si+M=64$$

$$\left( Si:M \right)M+M=64$$

This can be used to calculate the number of metal atoms per unit cell incorporated into the formed (SiOH)_x_. The final (SiOH)_x_ (unit cell)^-1^ for Sn-BEA is equal to the initial amount of Al (assumed that all Al is removed) subtracted by the added Sn. In contrast, the (SiOH)_x_ (unit cell)^-1^ for the Brønsted acid equals the initial quantity of Al subtracted by the amount of Al removed:

$$\left( SiOH \right)_{x, Sn-BEA}=3.047 -added Sn$$

$$\left( SiOH \right)_{x, Sn-BEA}=3.047 -removed Al$$

Table S3 reports metal loadings per unit cell from ICP and calculated (SiOH)_x_ per unit cell. The calculated values predict a wider range of zeolite hydrophilicity than the Φ_IR_ calculations in Section S3.5. However, rates depend weakly on metal loading (Section S5, *vide infra*), and therefore (SiOH)_x_ (unit cell)^-1^, within the range of loadings examined for M-BEA.

| Catalyst | M (unit cell)^-1^ | (SiOH)_x_ (unit cell)^-1^ |
| --- | --- | --- |
| Al-BEA-OH | 0.86 | 2.17 |
| Zr-BEA-OH | 0.47 | 2.57 |

**Table S3**. Metal loadings and (SiOH)_x_ per unit cell, determined from metal loadings from ICP and estimated Al removed by dealumination treatments.

**S3.7 CD_3_CN and C_5_H_5_N Infrared Adsorption Spectra to Examine Acid Site Character**


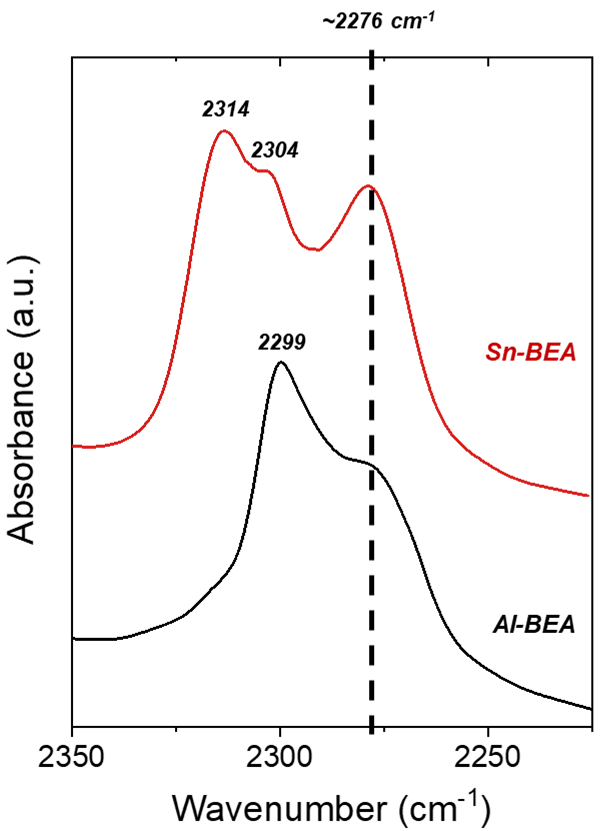


**Figure S7.** Infrared spectra of deuterated acetonitrile (CD_3_CN) bound to M-BEA (Sn (red), Al (black)) materials (0.05 kPa CD_3_CN, 101 kPa Ar, 303 K). All spectra are normalized to the area of the Si-O-Si region of the zeolite from 1750-2100 cm^-1^. Spectra are vertically offset for clarity.

The feature at ~2299 cm^-1^ for Al-BEA is consistent with previous assignments for CD_3_CN bound to Brønsted acidic Al sites.^54-55^ Al-BEA shows a shoulder at ~2276 cm^-1^ that has been reported to result from CD_3_CN coordinated to (SiOH)_x_ nests within *BEA.^54, 56^ Extraframework Lewis acidic Al species have been reported to show a feature at ~2325 cm^-1^,^54-55^ where no feature is observed in Al-BEA. The spectra suggests that the Al atoms in Al-BEA predominantly exist as tetrahedrally incorporated Brønsted acid sites.

Sn-BEA also shows a feature at ~2276 cm^-1^ that likely originates from CD_3_CN bound to Si-OH groups. Sn-BEA also displays features at ~2304 and ~2314 cm^-1^, which align with previous proposals for closed and open tetrahedral Sn sites within zeolites, respectively.^57-59^ While the presence of these two features demonstrates a mix of Sn site structures under an inert gaseous environment, the ratio of open and closed sites may change in the presence of solvent. Several past studies argue that closed Lewis acid sites may open in the presence of protic molecules such as H_2_O^60-62^ and alcohols,^63-64^ meaning that Sn sites can open *in situ* in the presence of CH_3_OH and convolute the *ex-situ* site distribution.

Collectively, the spectra of adsorbed CD_3_CN in Figure S7 demonstrate the presence of Lewis and Brønsted acid sites within the M-BEA materials.


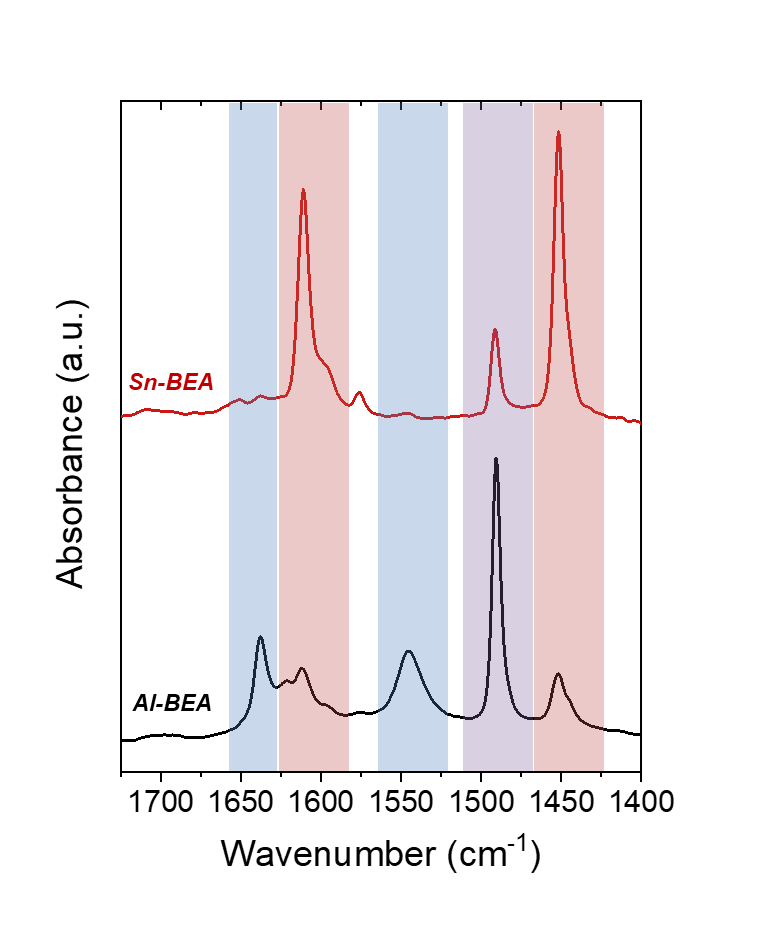


**Figure S8.** Infrared spectra of pyridine (C_5_H_5_N) bound to Lewis and Brønsted acid sites over M-BEA (Sn (red), Al (black)) materials (0.9 kPa C_5_H_5_N, 101 kPa Ar, 393 K). All spectra are normalized to the area of the Si-O-Si region of the zeolite from 1750-2100 cm^-1^. The highlighted regions on the plot represent regions for C_5_H_5_N absorbance features coordinated to only Brønsted acid sites (blue), only Lewis acid sites (red), and either Lewis or Brønsted acid sites (purple). Spectra are vertically offset for clarity.

Figure S8 reveals that Al-BEA and Sn-BEA both possess features at ~1490 cm^-1^, corresponding to C_5_H_5_N bound to Lewis or Brønsted acid sites.^65-66^ Each catalyst also possesses features at ~1450 cm^-1^ and 1580-1625 cm^-1^, which have been attributed to C_5_H_5_N bound to Lewis acid sites.^65, 67-72^ The presence of these features in Al-BEA may signal the presence of some fraction of extraframework Lewis acidic Al species, in contrast with the absence of Lewis acid Al features on the CD_3_CN spectra. Al-BEA also shows features at ~1550 and ~1640 cm^-1^, which solely originate from protonated C_5_H_5_N at Brønsted acid sites.^65-69^ Sn-BEA contains very minor features in these regions, which may suggest that a minor fraction of Sn sites possess Brønsted acid character. Sn-BEA and Al-BEA show small features at ~1575 cm^-1^, which has been attributed to physisorbed C_5_H_5_N.^65, 69, 73^

Sn-BEA contains a sharp feature at ~1611 cm^-1^, which has previously been assigned to C_5_H_5_N coordinated to Lewis acidic Sn sites.^59, 74-75^ Sn-BEA also shows a shoulder at ~1595 cm^-1^, consistent with previous assignments for C_5_H_5_N hydrogen-bonded to -OH groups.^66, 68-69, 71^ These features may arise from C_5_H_5_N coordinated to the -OH group from Sn-OH (open sites), the adjacent Si-OH, or (SiOH)*_x_* nests.

Overall, the spectra of adsorbed C_5_H_5_N in Figure S8 provide insight into the distribution of active sites within M-BEA.

**S4. Calculation and Estimation of Epoxide and Product Sensitivity Factors**


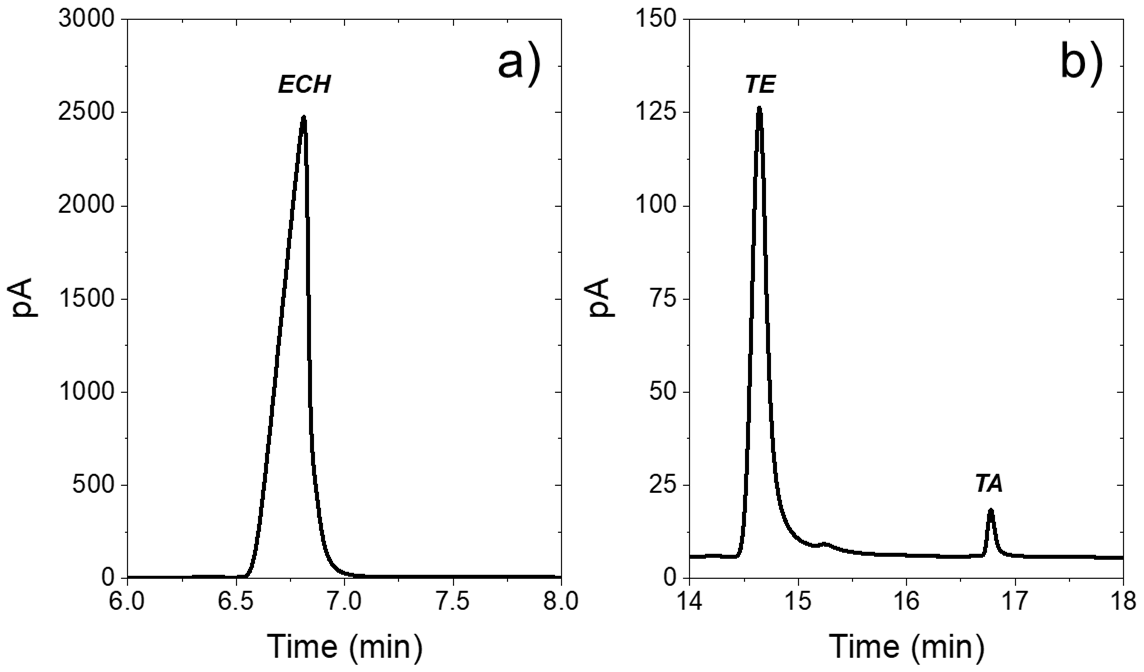


**Figure S9.** Raw GC chromatograms zoomed in to show peaks corresponding to (a) C_3_H_5_ClO, and (b) the terminal ether and terminal alcohol formed by the reaction of C_3_H_5_ClO with CH_3_OH over Sn-BEA for 0.5 h (0.2 M C_3_H_5_ClO, 24.7 CH_3_OH, 308 K).

Figure S9 demonstrates the high signal-to-noise ratio of C_3_H_5_ClO and the TE and TA product peaks from the GC chromatograms, as well as the peak separation between the ring-opening products. GC chromatograms were collected as a function of concentrations, which were used to determine the sensitivity factors of the epoxide and TE product using as-purchased chemicals (see Section S1.3 for sources and purities). Table S4 reports the sensitivity factors for these species, which equal the slope of the calibration curve fit lines for the GC peak area as a function of species concentration. A commercial sample for the TA product was not available for purchase, so we assumed that TA and TE have identical sensitivity factors. Our previous work showed that the sensitivity factors for the TA and TE products from C_4_H_8_O differ by less than 5%, so we expect the products from C_3_H_5_ClO will show very similar values as well.^19^

| Compound | Sensitivity Factor (peak area M^-1^) |
| --- | --- |
| C_3_H_5_ClO | 85210 |
| TE | 63060 |

**Table S4**. Sensitivity factors determined from calibration curves.

During reactions, the concentration of the products was calculated with the integrated peak areas and measured sensitivity factors:

$\left[ product \right]=\frac{GC Peak Area}{Sensitivity Factor}$ (S2)


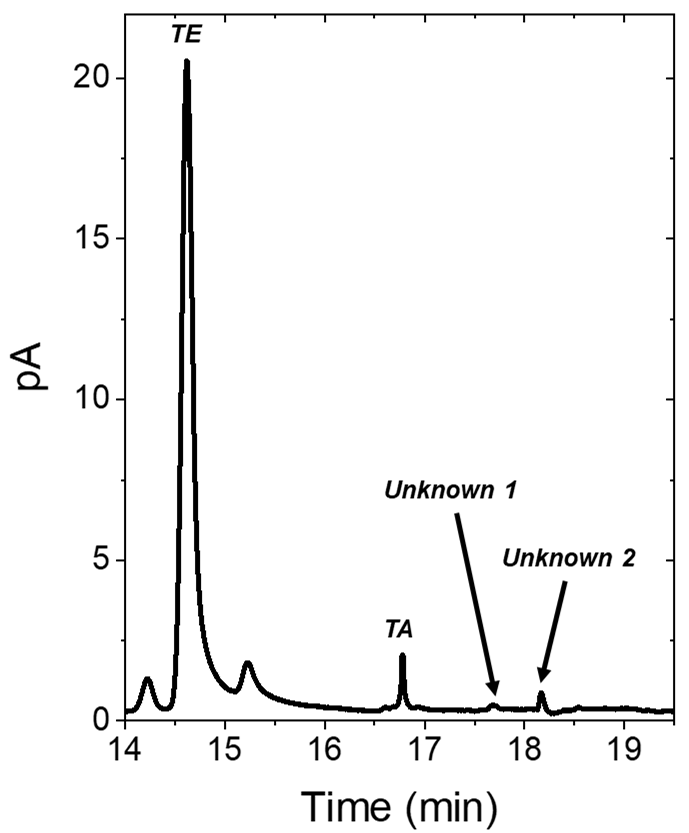


**Figure S10.** Raw GC chromatograms zoomed in to show peaks corresponding to the terminal ether product, terminal alcohol product, and unknown peaks that appear during the reaction of C_3_H_5_ClO with CH_3_OH over Sn-BEA for 0.5 h (0.2 M C_3_H_5_ClO, 1 M CH_3_OH, 308 K).

Figure S10 shows a GC chromatogram from the reaction of C_3_H_5_ClO with CH_3_OH, where unknown peaks appear at 17.7 and 18.2 minutes. The unknown peaks account for < 5% of all products formed. While the unknown species have not been identified, the peaks may originate from secondary oligomers formed by the reaction of the ring-opening products reacting with epichlorohydrin.

**S5. Madon-Boudart Criterion Test to Identify Active Centers for Ring-Opening**


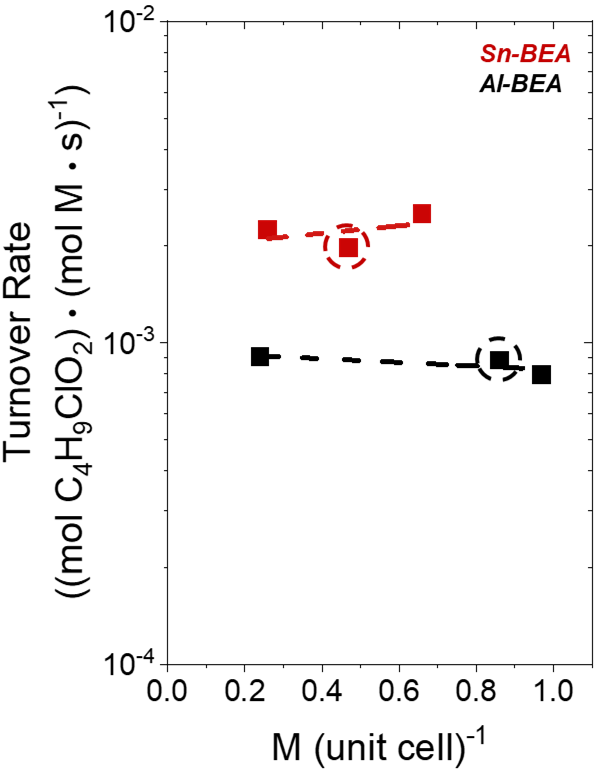


**Figure S11.** Total product formation rates from C_3_H_5_ClO ring-opening with CH_3_OH (0.07 M C_3_H_5_ClO, 0.4 M CH_3_OH, CH_3_CN solvent, 308 K) as a function of active metal loading over Al- (black) and Sn-BEA (red). The circled points signify the catalyst used for this study.

Figure S11 presents turnover rates as a function of metal loading for each M-BEA. Rates depend negligibly on metal loading within the range of loadings examined for all materials, ruling out contributions from intrapore mass transfer to turnover rate measurements.

**S6. XRD Analysis of Spent Catalyst and Hot Filtrations to Examine Catalyst Stability**


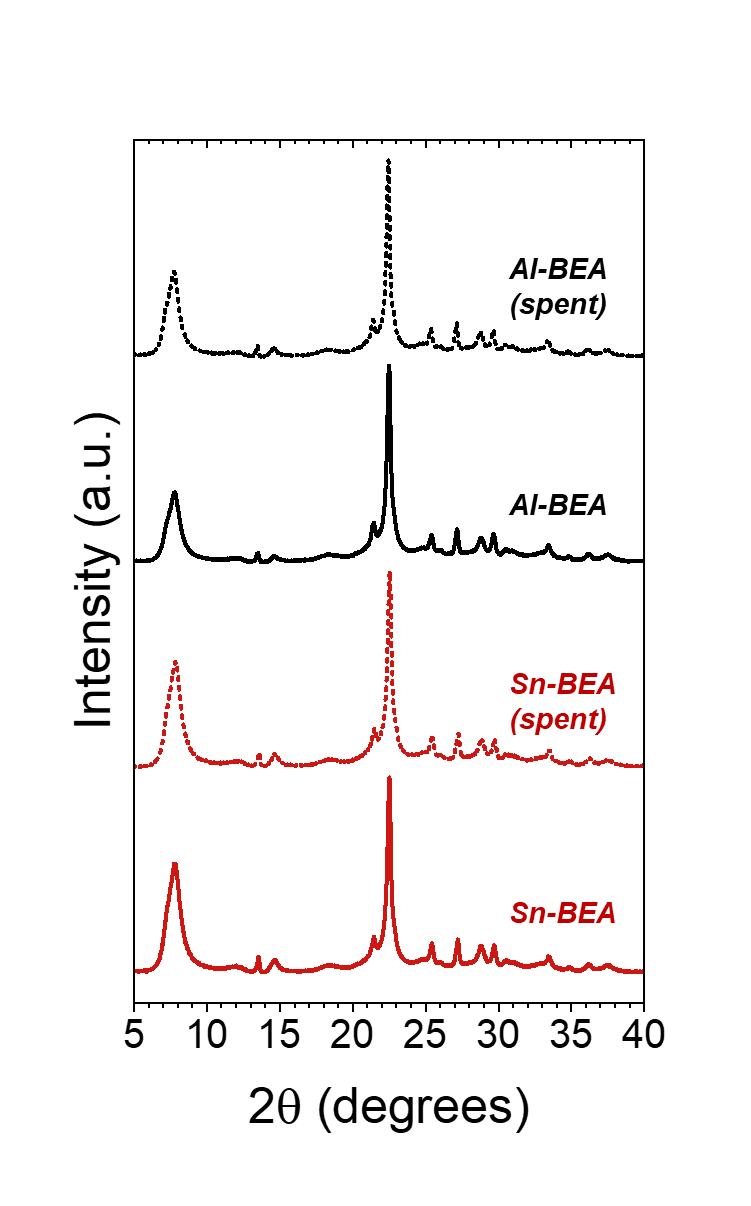


**Figure S12.** Powder X-ray diffractograms obtained before (solid lines) and after (hollow lines) the C_3_H_5_ClO ring-opening reaction with CH_3_OH (0.07 M C_3_H_5_ClO, 1 M CH_3_OH, CH_3_CN solvent, 308 K) over Al- (black) and Sn-BEA (red). The reaction was carried out for 24 hours, then the catalyst was recovered and dried by vacuum filtration. Spectra were collected with Cu Kα radiation (0.15418 nm) under ambient conditions, and are vertically offset for clarity.

Figure S12 presents X-ray diffraction patterns for M-BEA before and after the reaction of C_3_H_5_ClO with CH_3_OH over 24 hours. The fresh and spent Al-BEA and Sn-BEA materials show nearly identical peak locations and intensities, supporting that contacting these catalysts with the reaction mixture does not cause structural changes or catalyst deactivation.


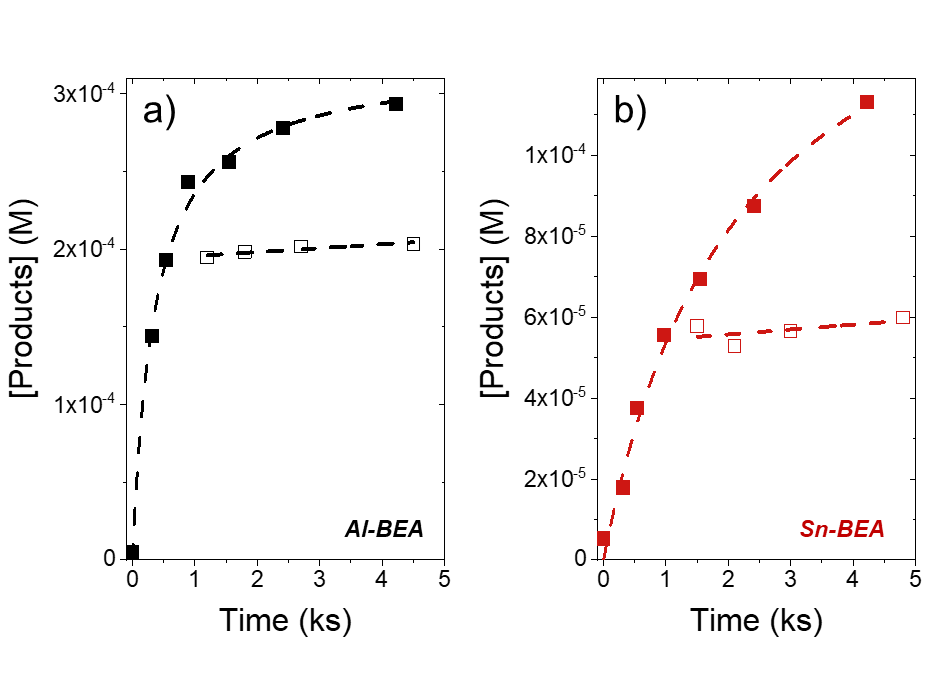


**Figure S13.** Measured total product concentrations (sum of [1M2B] and [2M1B]) as functions of time during hot filtration tests for 1,2-epoxybutane (C_4_H_8_O) ring-opening (0.005 M C_4_H_8_O, 6 M CH_3_OH, CH_3_CN solvent, 308 K) over (a) Al-BEA (black) and (b) Sn-BEA (red). The hollow points represent epoxide concentrations from aliquots that were separated from the catalyst after ~10-15 min, while solid points indicate concentrations from aliquots that were kept in contact with the catalyst throughout the reaction.

Figure S13 displays the results of hot filtration tests for Al-BEA and Sn-BEA during the ring-opening of 1,2-epoxybutane (C_4_H_8_O) with CH_3_OH, taken from our previous work.^19^ After separating the solution from the catalyst, the concentration of the ring-opening products stops increasing with time (hollow points). This result provides evidence that metal atoms do not leach from the *BEA framework, and the active sites remain within the framework during catalysis. While these experiments utilize C_4_H_8_O instead of C_3_H_5_ClO, we argue that the experiments support that the active sites in Al-BEA and Sn-BEA will also remain coordinated to the zeolite framework during C_3_H_5_ClO ring-opening with CH_3_OH.

**S7. Counting Active Sites with 1,2-Diphenyl-1,2-Ethylenediamine Titrations**

**
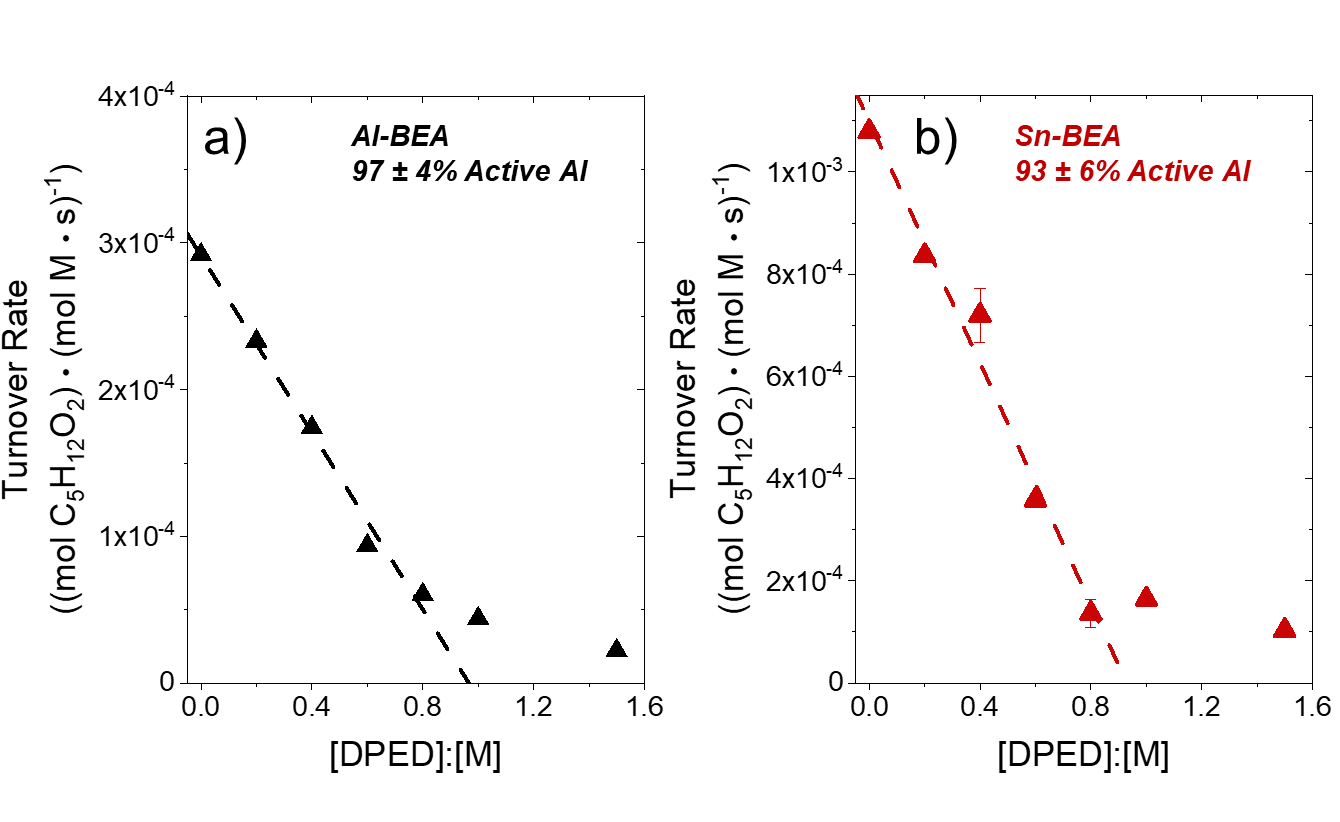
**

**Figure S14.** Total product formation rates from C_4_H_8_O ring-opening with CH_3_OH (0.005 M C_4_H_8_O, 0.4 M CH_3_OH, CH_3_CN solvent, 308 K) as a function of 1,2-diphenyl-1,2-ethylenediamine (DPED) to active metal ratio over (a) Al-BEA (black) and (b) Sn-BEA (red). The data for Al-BEA was reported in a previous manuscript.^23^

1,2-diphenyl-1,2-ethylenediamine (DPED) is a titrant that could poison active sites in both Lewis and Brønsted acidic M-BEA, which was utilized to count active sites during C_4_H_8_O ring-opening. A line was fit to the linear portion of the plots in Figure S14 and extrapolated to the x-axis to estimate the number of active sites present in the M-BEA materials. All materials show greater than 90% active metal atoms, providing evidence that all (or nearly all) metal atoms are tetrahedrally bound to the *BEA framework and catalyze ring-opening. We anticipate that the M-BEA materials would show a similarly high quantity of active metal atoms during C_3_H_5_ClO ring-opening.

**S8. Derivation of Rate Expression for C_3_H_5_ClO Ring-Opening with CH_3_OH over M-BEA**

| Catalyst | [CH_3_OH]:[C_3_H_5_ClO] < 15 | | [CH_3_OH]:[C_3_H_5_ClO] > 30 | |
| --- | --- | --- | --- | --- |
|  | TE | TA | TE | TA |
| Al-BEA | [CH_3_OH]**^1.05^**  [C_3_H_5_ClO]^0.14^ | [CH_3_OH]**^0.58^**  [C_3_H_5_ClO]^0.19^ | [CH_3_OH]**^0.29^**  [C_3_H_5_ClO]^0.77^ | [CH_3_OH]**^0.24^**  [C_3_H_5_ClO]^0.83^ |
| Sn-BEA | [CH_3_OH]**^0.99^**  [C_3_H_5_ClO]^0.16^ | [CH_3_OH]**^0.45^**  [C_3_H_5_ClO]^0.22^ | [CH_3_OH]**^0.19^**  [C_3_H_5_ClO]^0.83^ | [CH_3_OH]**^0.10^**  [C_3_H_5_ClO]^0.80^ |

**Table S5.** Formation Rate Orders of TE and TA for C_3_H_5_ClO ring-opening from Figure 1 in main text.

The formation rates of TE and TA derived from C_3_H_5_ClO show clear differences in their dependence on the concentration of the nucleophile (i.e., [CH_3_OH]), which contrasts with prior studies of epoxide ring-opening on solid catalysts. The apparent power-law dependencies for TE and TA formation differ significantly (by 0.5 – 0.6) at [CH_3_OH]:[C_3_H_5_ClO] < 15.

Scheme S2 and Scheme 1 in the main text depict the proposed steps for the ring-opening of C_3_H_5_ClO with CH_3_OH over Sn-BEA and Al-BEA, respectively. The catalytic cycle begins with the adsorption of CH_3_CN (step 1, not shown), C_3_H_5_ClO (step 2), or CH_3_OH (step 3). The products may form through adsorbed CH_3_OH in a kinetically relevant S_N_2 reaction step (4). Alternatively, an S_N_1 pathway may occur, where adsorbed C_3_H_5_ClO irreversibly ring-opens to form a primary or secondary carbocation (step 5). The products then form when CH_3_OH attacks the adsorbed carbocations (step 6). The products desorb in reversible steps (7 and 8) to complete the cycle. The total rates of product formation from C_3_H_5_ClO ring-opening rates can be modeled with:


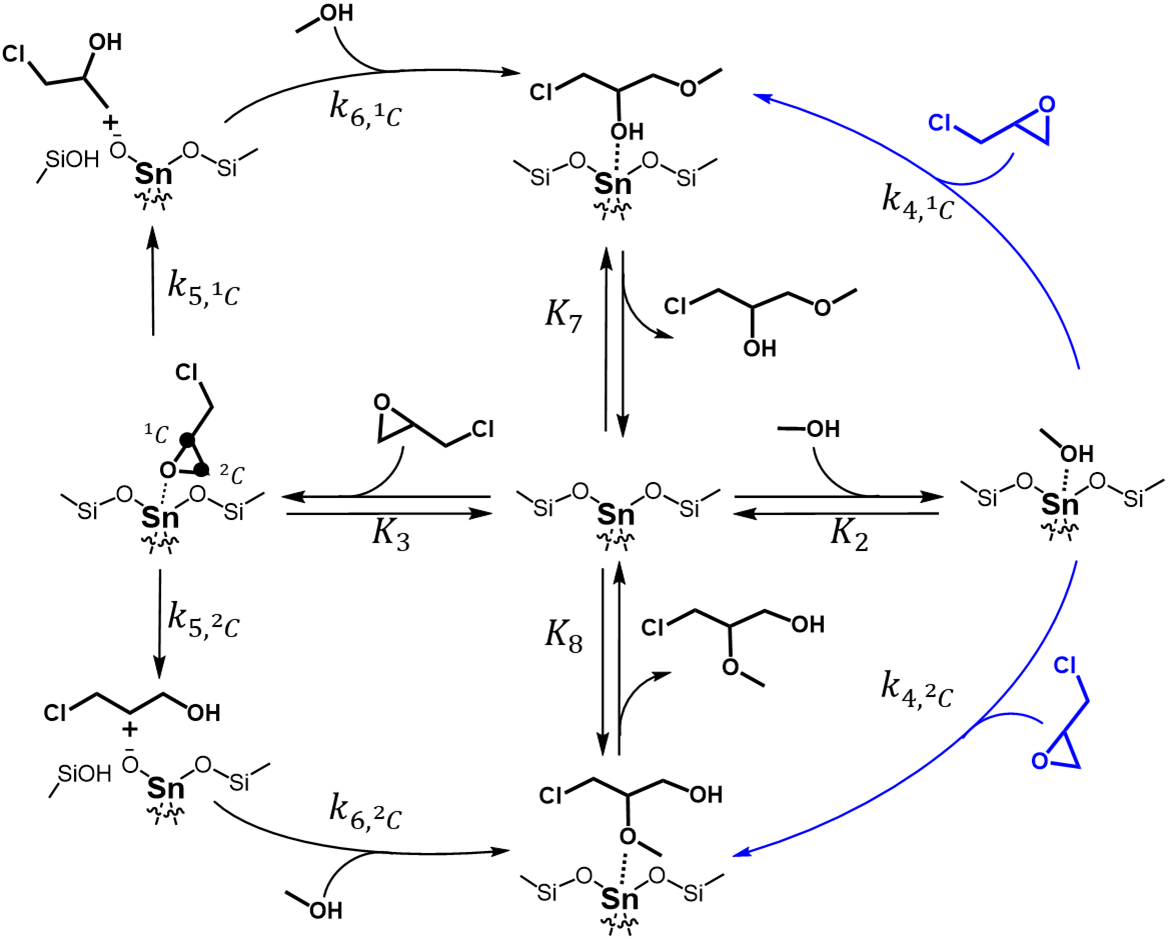


**Scheme S2**. Plausible product-forming steps for ring-opening of C_3_H_5_ClO with CH_3_OH over Sn-BEA. Reaction may proceed through a concerted S_N_2 pathway with an activated CH_3_OH intermediate (blue) or a stepwise S_N_1 pathway in which C_3_H_5_ClO ring-opens to form carbocations, followed by adsorption and activation of CH_3_OH (black). For brevity, we do not show the reversible adsorption of CH_3_CN molecules (step 1) in this cycle.

$r_{RO}= r_{4}+r_{6}=k_{4}\left[ C_{3}H_{5}ClO \right]\left[ CH_{3}OH^{*} \right]+(k_{6,{}^{1}C}\left[ {C_{1+}}^{*} \right]+k_{6,{}^{2}C}\left[ {C_{2+}}^{*} \right])\left[ CH_{3}OH \right]$ (S3)

where $\left[ {C_{1+}}^{*} \right]$ and $\left[ {C_{2+}}^{*} \right]$ represent the numbers of primary and secondary ring-opened carbocations formed from steps with the rate constants $k_{5,{}^{1}C}$ and $k_{5,{}^{2}C}$, respectively. The term $k_{4}$ represents the sum of the rate constants to form the products in step 4 ($k_{4}=k_{4,{}^{1}C}+k_{4,{}^{2}C}$). We apply the pseudo-state hypothesis to the respective bound intermediates, which gives:

$r_{RO}=\frac{k_{2}k_{4}\left[ C_{3}H_{5}ClO \right]\left[ CH_{3}OH \right][*]}{k_{-2} +k_{4}\left[ C_{3}H_{5}ClO \right]} + \frac{k_{3}k_{5,{}^{1}C}\left[ C_{3}H_{5}ClO \right][*]}{k_{-3} + k_{5}}+ \frac{k_{3}k_{5,{}^{2}C}\left[ C_{3}H_{5}ClO \right][*]}{k_{-3} + k_{5}}$ (S4)

The $r_{6}$ term expands to two terms to account for the formation of the terminal ether and terminal alcohol products. The total number of active sites in each M-BEA ([L]) can be written as:

$\left[ L \right]=\left[ * \right]+\left[ CH_{3}CN^{*} \right]+\left[ CH_{3}OH^{*} \right]+\left[ C_{3}H_{5}{ClO}^{*} \right]+\left[ {C_{1+}}^{*} \right]+\left[ {C_{2+}}^{*} \right]+\left[ {TE}^{*} \right]+\left[ {TA}^{*} \right]$ (S5)

Where $\left[ * \right]$ represents the number of unoccupied active sites and the other terms represent the number of adsorbed solvent, reactant, and product molecules. Substituting in terms for the solvent, product, and reactant concentrations, rate constants, and equilibrium constants yields:

$\frac{\left[ L \right]}{\left[ * \right]}=1+K_{1}\left[ CH_{3}CN \right]+\frac{k_{2}\left[ CH_{3}OH \right]}{k_{-2}+k_{4}\left[ C_{3}H_{5}ClO \right]}+\frac{k_{3}\left[ C_{3}H_{5}ClO \right]}{k_{-3}+k_{5}}+\frac{k_{3}k_{5,{}^{1}C}\left[ C_{3}H_{5}ClO \right]}{k_{6,{}^{1}C}\left[ CH_{3}OH \right]\left( k_{-3} + k_{5} \right)}+\frac{k_{3}k_{5,{}^{2}C}\left[ C_{3}H_{5}ClO \right]}{k_{6,{}^{2}C}\left[ CH_{3}OH \right]\left( k_{-3} + k_{5} \right)}+\frac{\frac{k_{3}k_{5,{}^{1}C}k_{6,{}^{1}C}\left[ C_{3}H_{5}ClO \right]}{k_{6,{}^{1}C}\left[ CH_{3}OH \right]\left( k_{-3} + k_{5} \right)}+\frac{k_{2}k_{4,{}^{1}C}\left[ CH_{3}OH \right]}{k_{-2}+k_{4,{}^{1}C}\left[ C_{3}H_{5}ClO \right]}+k_{-8}\left[ TE \right]}{k_{7}}+\frac{\frac{k_{3}k_{5,{}^{2}C}k_{6,{}^{2}C}\left[ C_{3}H_{5}ClO \right]}{k_{6,{}^{2}C}\left[ CH_{3}OH \right]\left( k_{-3} + k_{5} \right)}+\frac{k_{2}k_{4,{}^{2}C}\left[ CH_{3}OH \right]}{k_{-2}+k_{4,{}^{2}C}\left[ C_{3}H_{5}ClO \right]}+k_{-8}\left[ TA \right]}{k_{8}}$ (S6)

$r_{RO}=\frac{\frac{k_{2}k_{4}\left[ C_{3}H_{5}ClO \right]\left[ CH_{3}OH \right]}{k_{-2} +k_{4}\left[ C_{3}H_{5}ClO \right]} + \frac{k_{3}k_{5,{}^{1}C}\left[ C_{3}H_{5}ClO \right]}{k_{-3} + k_{5}}+ \frac{k_{3}k_{5,{}^{2}C}\left[ C_{3}H_{5}ClO \right]}{k_{-3} + k_{5}}}{\frac{\left[ L \right]}{\left[ * \right]}}$ (S7)

The total rate of product formation depends linearly on [CH_3_OH] and weakly on [C_3_H_5_ClO] at ratios of [CH_3_OH] to [C_3_H_5_ClO] less than 15 over each M-BEA, implicating active sites saturated with a C_3_H_5_ClO-derived species. In this case, we assume that the [C_3_H_5_ClO*], [C_1+_*], and [C_2+_*] terms dominate in the denominator of Equation S7, allowing other terms to be canceled out:

$r_{RO}=\frac{\frac{k_{2}k_{4}\left[ C_{3}H_{5}ClO \right]\left[ CH_{3}OH \right]}{k_{-2} +k_{4}\left[ C_{3}H_{5}ClO \right]} + \frac{k_{3}k_{5,{}^{1}C}\left[ C_{3}H_{5}ClO \right]}{k_{-3} + k_{5}} + \frac{k_{3}k_{5,{}^{2}C}\left[ C_{3}H_{5}ClO \right]}{k_{-3} + k_{5}}}{\frac{k_{3}\left[ C_{3}H_{5}ClO \right]}{k_{-3}+k_{5}} + \frac{k_{3}k_{5,{}^{1}C}\left[ C_{3}H_{5}ClO \right]}{k_{6,{}^{1}C}\left[ CH_{3}OH \right](k_{-3} + k_{5})} + \frac{k_{3}k_{5,{}^{2}C}\left[ C_{3}H_{5}ClO \right]}{k_{6,{}^{2}C}\left[ CH_{3}OH \right](k_{-3} + k_{5})}}$ (S8)

The second and third terms in the numerator dominate when ratios of [CH_3_OH] to [C_3_H_5_ClO] are sufficiently low and the value of $k_{3}k_{5}$ far exceeds $k_{2}k_{4}$ (i.e., when adsorption and reaction of C_3_H_5_ClO overtakes the reaction through protonated CH_3_OH). Independent measurements of the adsorption enthalpies of these reactants obtained by ITC support this assumption: adsorption enthalpies for C_3_H_5_ClO to M-BEA (-120 to -150 kJ mol^-1^) are significantly more exothermic than those for CH_3_OH (~ 0 kJ mol^-1^) in CH_3_CN (Section S11). These thermodynamic measurements suggest that C_3_H_5_ClO adsorbs more readily than CH_3_OH (i.e., $k_{3}$ >> $k_{2}$). These insights collapse Equation S8 to the following form after combining and pulling out common terms:

$r_{RO}=\frac{\frac{k_{3}\left[ C_{3}H_{5}ClO \right]}{k_{-3} + k_{5}}\left( k_{5,{}^{1}C} + k_{5,{}^{2}C} \right)}{\frac{k_{3}\left[ C_{3}H_{5}ClO \right]}{k_{-3} + k_{5}}\left( 1 + \frac{k_{5,{}^{1}C}}{k_{6,{}^{1}C}\left[ CH_{3}OH \right]} + \frac{k_{5,{}^{2}C}}{k_{6,{}^{2}C}\left[ CH_{3}OH \right]} \right)}$ (S9)

From there, the expression can be simplified to a rate equation that agrees with experimental observations (Figure 1) and describes rates for S_N_1 pathways that proceed through protonated C_3_H_5_ClO as:

$r_{RO,S_{N}1}=\frac{\left( k_{5,{}^{1}C} + k_{5,{}^{2}C} \right)k_{6,{}^{1}C}\cdot k_{6,{}^{2}C}\left[ CH_{3}OH \right]}{\left( k_{5,{}^{1}C}{\cdot k}_{6,{}^{2}C} \right) +\left( k_{5,{}^{2}C}\cdot k_{6,{}^{1}C} \right) +\left( k_{6,{}^{1}C}\cdot k_{6,{}^{2}C}\left[ CH_{3}OH \right] \right)}$ (S10)

Notably, the form of Equation S10 implies that the number of ring-opened carbocation species derived from C_3_H_5_ClO exceeds the number of C_3_H_5_ClO* that retain the oxirane ring, which agrees with *in situ* NMR spectra (*vide infra*). Table S5 shows that TE formation rates increase linearly and TA formation rates increase sub-linearly with [CH_3_OH], which implies that the rate constant to form a ring-opened primary carbocation intermediate (e.g., $k_{5,{}^{1}C}$) exceeds that to remove that intermediate from the active site (i.e., $k_{6,{}^{1}C}$). In contrast, $k_{5,{}^{2}C}$ and $k_{6,{}^{2}C}$ possess similar magnitudes to provide a sublinear dependence on [CH_3_OH] for TA formation.

When CH_3_OH-derived species saturate active sites, Equation S7 simplifies to:

$r_{RO}=\frac{\frac{k_{2}k_{4}\left[ C_{3}H_{5}ClO \right]\left[ CH_{3}OH \right]}{k_{-2} +k_{4}\left[ C_{3}H_{5}ClO \right]} + \frac{k_{3}k_{5,{}^{1}C}\left[ C_{3}H_{5}ClO \right]}{k_{-3} + k_{5}} + \frac{k_{3}k_{5,{}^{2}C}\left[ C_{3}H_{5}ClO \right]}{k_{-3} + k_{5}}}{\frac{k_{2}\left[ CH_{3}OH \right]}{k_{-2} +k_{4}\left[ C_{3}H_{5}ClO \right]}}$ (S11)

Equation S11 reproduces the observations for rates and describes S_N_2 pathways ($r_{4}$) when the first term in the numerator dominates, which occurs when catalysis proceeds at sufficiently high ratios of [CH_3_OH] to [C_3_H_5_ClO] (>30). These conditions simplify Equation S11 to the form:

$r_{RO,S_{N}2}=k_{4}\left[ C_{3}H_{5}ClO \right]$ (S12)

which predicts both TA and TE products form at rates proportional to [C_3_H_5_ClO] and independent of [CH_3_OH]. The form of Equation S12 deviates slightly from the measured dependence of rate on [C_3_H_5_ClO] and [CH_3_OH] (Figure 1 and Table S5). These differences indicate that the number of CH_3_OH* species do not achieve much greater values than C_3_H_5_ClO*-derived intermediates even at the greatest concentration ratios examined, which agrees with expectations based on the more exothermic adsorption of C_3_H_5_ClO relative to CH_3_OH (*vide supra*).

TE and TA formation show very similar rate orders with respect to [CH_3_OH] at [CH_3_OH]:[C_3_H_5_ClO] >30. As such, the significant preference towards the TE product at the highest [CH_3_OH] in Figure 7 (*β* > 18) must occur because $k_{4,{}^{1}C}$ >> $k_{4,{}^{2}C}$. The S_N_2 pathway therefore dominantly forms the TE product due to differences in the rate constants for product formation.

Equations S10 and S12 describe the two kinetic regimes observed for TA and TE formation in Figure 1 of the main text.

Equation S3 can be utilized to define the regioselectivity through the rate ratio of terminal ether to terminal alcohol formation (*β*):

$\beta=\frac{r_{TE}}{r_{TA}}=\frac{k_{4,{}^{1}C}\left[ C_{3}H_{5}ClO \right]\left[ CH_{3}OH^{*} \right] + k_{6,{}^{1}C}\left[ {C_{1+}}^{*} \right]\left[ CH_{3}OH \right]}{k_{4,{}^{2}C}\left[ C_{3}H_{5}ClO \right]\left[ CH_{3}OH^{*} \right] + k_{6,{}^{2}C}\left[ {C_{2+}}^{*} \right]\left[ CH_{3}OH \right]}$ (S13)

As described in the main text, the NMR spectra suggest that $k_{4,{}^{2}C}$ shows the lowest value of all rate constants in Equation S13, allowing the expression to simplify to:

$\beta=\frac{r_{TE}}{r_{TA}}=\frac{k_{4,{}^{1}C}\left[ C_{3}H_{5}ClO \right]\left[ CH_{3}OH^{*} \right] + k_{6,{}^{1}C}\left[ {C_{1+}}^{*} \right]\left[ CH_{3}OH \right]}{k_{6,{}^{2}C}\left[ {C_{2+}}^{*} \right]\left[ CH_{3}OH \right]}$ (S14)

Substituting in terms for $\left[ CH_{3}OH^{*} \right]$, $\left[ {C_{1+}}^{*} \right]$, and $\left[ {C_{2+}}^{*} \right]$ then simplifying the expression yields:

$\beta=\frac{r_{TE}}{r_{TA}}=\frac{\frac{k_{2}k_{4,{}^{1}C}\left[ C_{3}H_{5}ClO \right]\left[ CH_{3}OH \right]}{k_{-2} +k_{4}\left[ C_{3}H_{5}ClO \right]} + \frac{k_{3}k_{5,{}^{1}C}\left[ C_{3}H_{5}ClO \right]}{k_{-3} + k_{5}}}{\frac{k_{3}k_{5,{}^{2}C}\left[ C_{3}H_{5}ClO \right]}{k_{-3} + k_{5}}}$ (S15)

Finally, combining terms yields a simplified expression for *β*:

$\beta=\frac{r_{TE}}{r_{TA}}=\frac{k_{2}k_{4,{}^{1}C}\left[ CH_{3}OH \right]\left( k_{-3} + k_{5} \right)}{k_{3}k_{5,{}^{2}C}\left( k_{-2} +k_{4}\left[ C_{3}H_{5}ClO \right] \right)} +\frac{k_{5,{}^{1}C}}{k_{5,{}^{2}C}}$ (S16)

Equation S16 predicts a linear dependence of *β* on $\left[ CH_{3}OH \right]$ and a negative, sublinear dependence on $\left[ C_{3}H_{5}ClO \right]$. As discussed in the main text, this equation captures the linear dependence of *β* on $\left[ CH_{3}OH \right]$ shown in Figure 7a of the main text. The weak dependence of $\beta$ on $\left[ C_{3}H_{5}ClO \right]$ (Figure 7b) suggests that CH_3_OH desorption proceeds much more rapidly than the S_N_2 reaction step that forms the ring-opened products with adsorbed CH_3_OH (i.e., $k_{-2}$ >> $k_{4}\left[ C_{3}H_{5}ClO \right]$). Under this assumption, Equation S16 simplifies to a form that clearly demonstrates the linear dependence of *β* on $\left[ CH_{3}OH \right]$ and negligible dependence on $\left[ C_{3}H_{5}ClO \right]$ shown in Figure 7 of the main text:

$\beta=\frac{r_{TE}}{r_{TA}}=\frac{K_{2}k_{4,{}^{1}C}\left[ CH_{3}OH \right]\left( k_{-3} + k_{5} \right)}{k_{3}k_{5,{}^{2}C}} +\frac{k_{5,{}^{1}C}}{k_{5,{}^{2}C}}$ (S17)

Scheme S2 depicts Sn in both closed (4-coordinated) and open (Sn-OH, 3-coordinated) configurations. The roles of the two site populations in the S_N_1 and S_N_2 pathways cannot be isolated, but previous literature can provide valuable insight into which sites provide greater catalytic activity. Brunelli, Kulkarni and coworkers^76^ reported barriers for S_N_1 and S_N_2 pathways of C_3_H_5_ClO ring-opening with CH_3_OH over Sn-BEA. All DFT simulations utilized closed sites, and the authors propose an S_N_1 pathway where CH_3_OH dissociates and an Sn-OH group forms while Sn remains 4-coordinated to the framework. The Sn-OH group protonates C_3_H_5_ClO but does not fully ring-open the epoxide before nucleophilic attack, likely leading to a less electrophilic center and a less favorable S_N_1 pathway than with the ring-opened carbocations that can form over open Sn-OH sites. This suggests that the closed Sn sites may also catalyze an S_N_1 pathway, but at lower rates than the open sites. Recently, experimental results from Brunelli et al. show that Sn-BEA materials with greater quantities of open Sn sites provide greater rate constants for ECH ring-opening with CH_3_OH at a fixed CH_3_OH:ECH ratio of 60.^77^ The large excess of CH_3_OH leads to S_N_2 being the dominant pathway, evidenced by terminal ether selectivities greater than 95% for all Sn-BEA catalysts. These findings suggests that open Sn-OH sites show greater reaction rates even under an S_N_2 pathway, although the reaction proceeds over closed sites as well. The CD_3_CN IR experiments in Figure S7 suggest that closed Sn sites remain the predominant site in *ex situ* conditions, but closed sites can hydrolyze to form open Sn-OH sites upon CH_3_OH dissociation at the site.^64^ This suggests that greater fractions of the more active Sn-OH sites may form in the presence of CH_3_OH under ring-opening reaction conditions, which may facilitate both S_N_1 and S_N_2 pathways.

**S9. Excess Energies to Probe Liquid-Phase C_3_H_5_ClO and CH_3_OH Stability**

|  | **C_3_H_5_ClO** | | | **CH_3_OH** | | |
| --- | --- | --- | --- | --- | --- | --- |
| Solvent | γ | $H^{\varepsilon}$ (kJ mol^-1^) | $G^{\varepsilon}$ (kJ mol^-1^) | γ | $H^{\varepsilon}$ (kJ mol^-1^) | $G^{\varepsilon}$ (kJ mol^-1^) |
| CH_3_CN | 0.82 | -0.54 | -0.53 | 2.64 | 2.39 | 2.53 |
| 6 M CH_3_OH (in CH_3_CN) | 0.83 | -0.93 | -0.50 | 1.77 | 2.43 | 1.48 |
| 12 M CH_3_OH (in CH_3_CN) | 0.92 | -0.81 | -0.21 | 1.29 | 0.9 | 0.66 |
| CH_3_OH | 1.63 | -0.73 | 1.27 | 1 | 0 | 0 |

**Table S6**. Activity coefficients (γ), excess enthalpies ($H^{\varepsilon}$), and excess free energies ($G^{\varepsilon}$) calculated at 308 K in mixtures of CH_3_CN and CH_3_OH. Activity coefficients were obtained with the UNIFAC method on ChemCAD.

Table S6 presents excess energies and activity coefficients for C_3_H_5_ClO and CH_3_OH in mixtures of CH_3_OH and CH_3_CN. The equations used to compute the excess energies from the activity coefficients are:

$G_{j}^{\varepsilon}=RT*\ln\left( \gamma_{j} \right)$ (S18)

$H_{j}^{\varepsilon}=-RT^{2}*\frac{d\ln\left( \gamma_{j} \right)}{dT}$ (S19)

where *j* represents C_3_H_5_ClO or CH_3_OH, R is the ideal gas constant, and T equals temperature. The differential term was obtained from activity coefficients between 303-323 K. The natural log of the activity coefficients was plotted against the temperature, and the slope of this line equaled the differential term.

γ values vary by factors of less than 2 and 3 for C_3_H_5_ClO and CH_3_OH, respectively, across the range of solvent compositions examined for ring-opening. Furthermore, both $H^{\varepsilon}$ and $G^{\varepsilon}$ values span a range of less than 3 kJ mol^-1^. The weak dependences of these values on the fractions of CH_3_OH and CH_3_CN support that the stability of reactive species and adsorbates in the liquid phase does not drive changes in ring-opening rates, activation enthalpies for ring-opening, and enthalpies of adsorption for C_3_H_5_ClO.

**S10. Activation Enthalpy and Entropy Measurements**


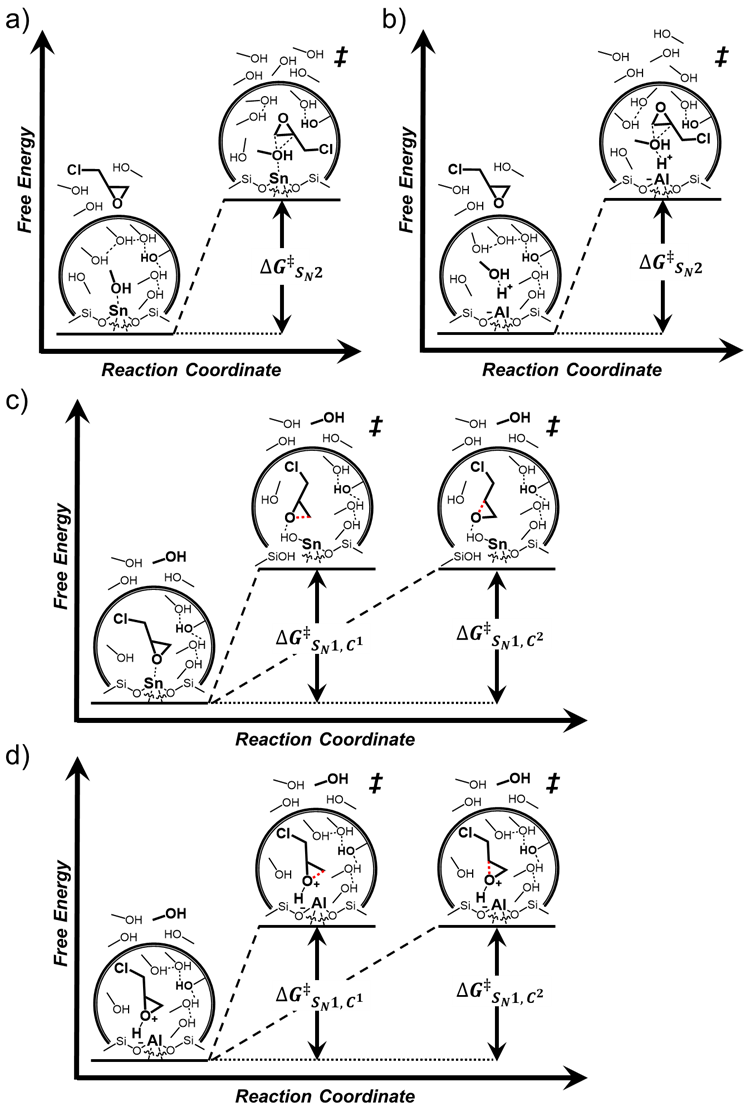


**Scheme S3.** Proposed reaction coordinate diagram for C_3_H_5_ClO ring-opening with CH_3_OH through an a) S_N_2 pathway over Sn-BEA, b) S_N_2 pathway over Al-BEA, c) S_N_1 pathway over Sn-BEA, d) S_N_1 pathway over Al-BEA. *Note*: we acknowledge that the reaction of CH_3_OH and the carbocations in c) and d) may also carry kinetic relevance.

Figure S15 presents activation barrier measurements for the formation of the terminal ether (TE) and terminal alcohol (TA) products from C_3_H_5_ClO ring-opening with CH_3_OH. The formation of TA shows more positive ${\Delta H}^{\ddagger}$ (by > 17 kJ mol^-1^) and ${\Delta S}^{\ddagger}$ (by > 40 J mol^-1^ K^-1^) values than TE at 0.07 M CH_3_OH. The significant differences for ${\Delta H}^{\ddagger}$ and ${\Delta S}^{\ddagger}$ suggests that distinct reaction pathways may govern product formation in this regime. S_N_1 pathways feature unimolecular transition states, which should lead to more positive ${\Delta S}^{\ddagger}$ values than S_N_2 pathways that react through bimolecular transition states (see Scheme S3 for proposed reaction pathways).^78^ An S_N_1 reaction pathway may dominate for TA formation at low [CH_3_OH] values while TE may form from a combination of S_N_1 and S_N_2 reactions. Interestingly, the difference between ${\Delta H}^{\ddagger}$ and ${\Delta S}^{\ddagger}$ for the product is less significant over Al-BEA than Sn-BEA in this low [CH_3_OH] regime, which may suggest that TE formation shows more S_N_1 character to provide more positive ${\Delta H}^{\ddagger}$ and ${\Delta S}^{\ddagger}$.


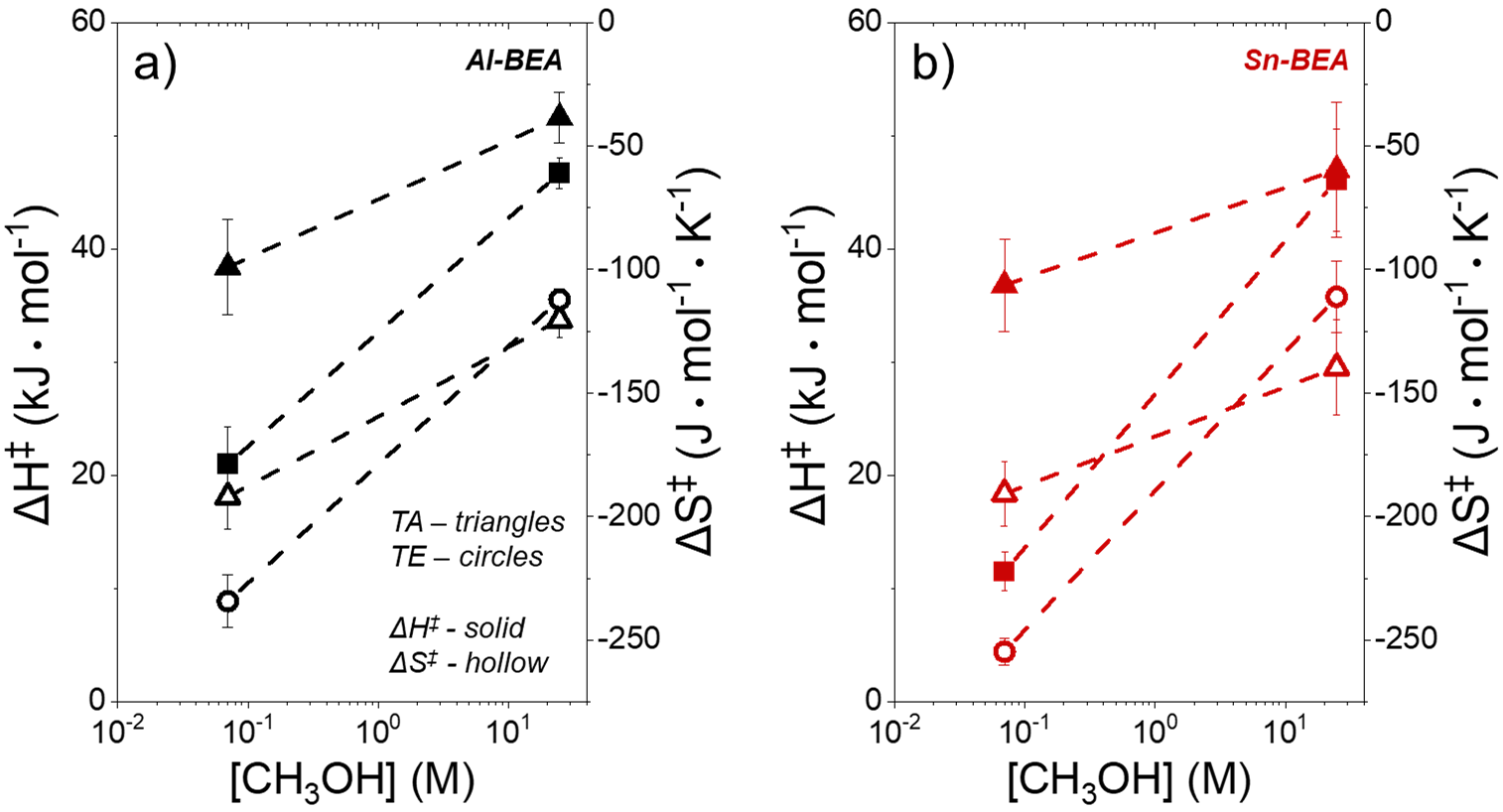
 **Figure S15**. Activation enthalpy and entropy to form the terminal ether (circles) and terminal alcohol (triangles) products from C_3_H_5_ClO ring-opening (0.07 M C_3_H_5_ClO, 298-328 K) over a) Al-BEA (black) and b) Sn-BEA (red).

In the absence of CH_3_CN co-solvent (24.7 M CH_3_OH), the products shift to very similar ${\Delta H}^{\ddagger}$ values (< 5 kJ mol^-1^). This similarity may suggest that both products shift towards similar dominant reaction pathways, which we expect would be an S_N_2 pathway through CH_3_OH-derived adsorbed species. Here, more positive ${\Delta S}^{\ddagger}$ values may drive the dominant formation of TE through lesser steric hindrance during the S_N_2 pathway.

While we argue that the differences in activation parameters between the products provide evidence for the shift in reaction pathways with [CH_3_OH] evidenced by the NMR spectra discussed in the main text, we cannot rule out that solvation effects may also play a role. We have previously argued that the disruption of hydrogen-bonded CH_3_OH molecules drives increases in ${\Delta H}^{\ddagger}$ and ${\Delta S}^{\ddagger}$ with [CH_3_OH] for C_4_H_8_O ring-opening over M-BEA materials. Interactions with surrounding solvent molecules may also play a role in C_3_H_5_ClO ring-opening and may affect the TE and TA formation transition states differently.

**S11. Isothermal Titration Calorimetry Thermograms and Heats of Adsorption**


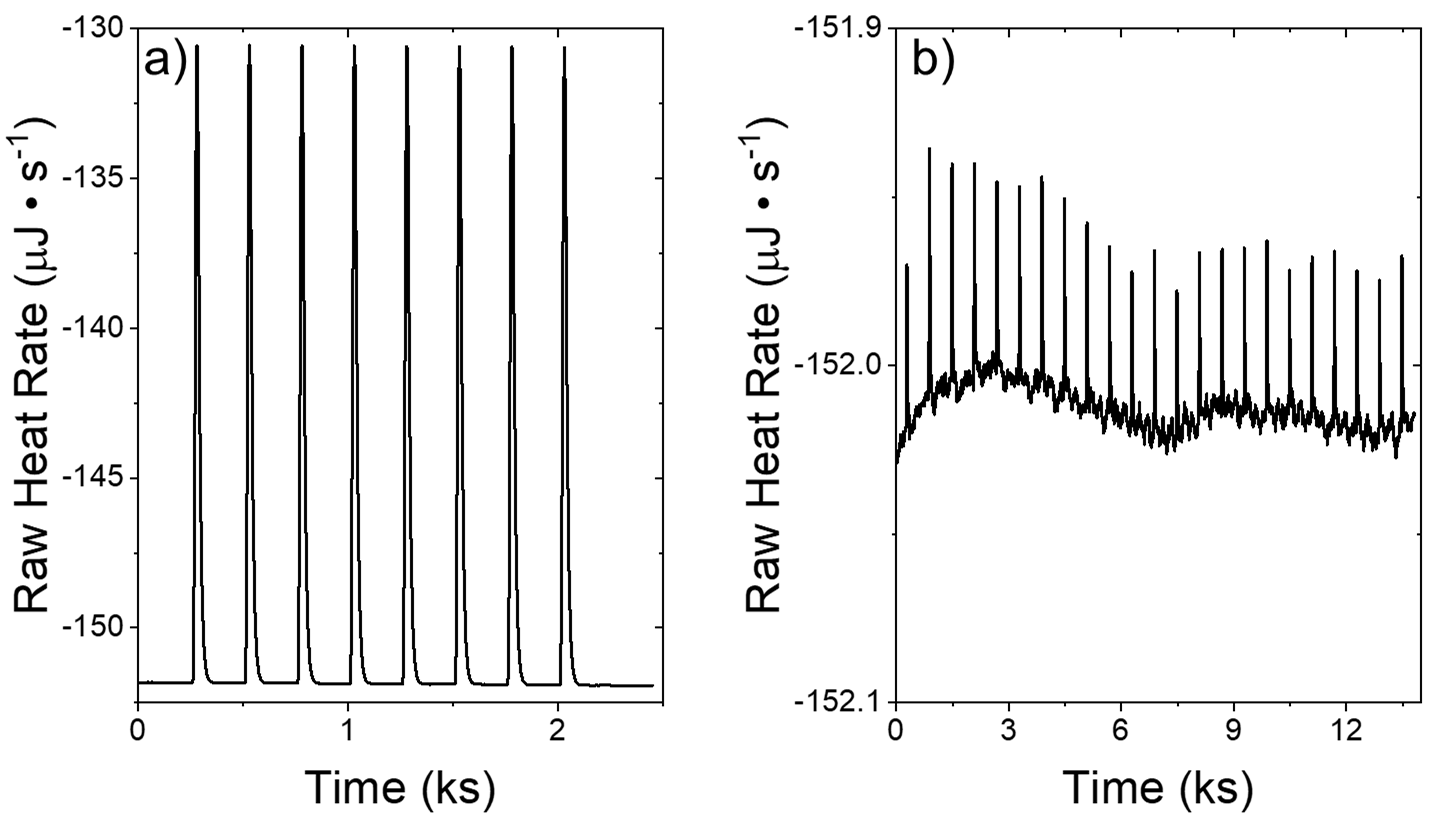


**Figure S16.** a) ITC thermogram from the electrical calibration of the NanoITC. b) ITC thermogram from the water-water adsorption to check the cleanliness of the ITC cell.

The plots in Figure S16 were obtained during the calibration and cleaning procedures for isothermal titration calorimetry (ITC), as discussed in Section S1.4. Figure S16a shows the heat released from the sequential pulses during the electrical calibration. The instrument gave a calibration factor, for which values greater than 0.98 were assumed to be satisfactory. Figure S16b shows the heat released during the water-water injection, during which each 1 μL injection led to a peak with an area between -3 and +3 μJ.

Figure S17 shows an acid-base titration of NaHCO_3_ into HNO_3_, which was used as a standard to verify the results obtained from the NanoITC. The adsorption enthalpy calculated from Figure S17b was -8.1 ± 1.2 kJ mol^-1^, which agrees well with the reported enthalpy of -9.1 kJ mol^-1^.^79-80^


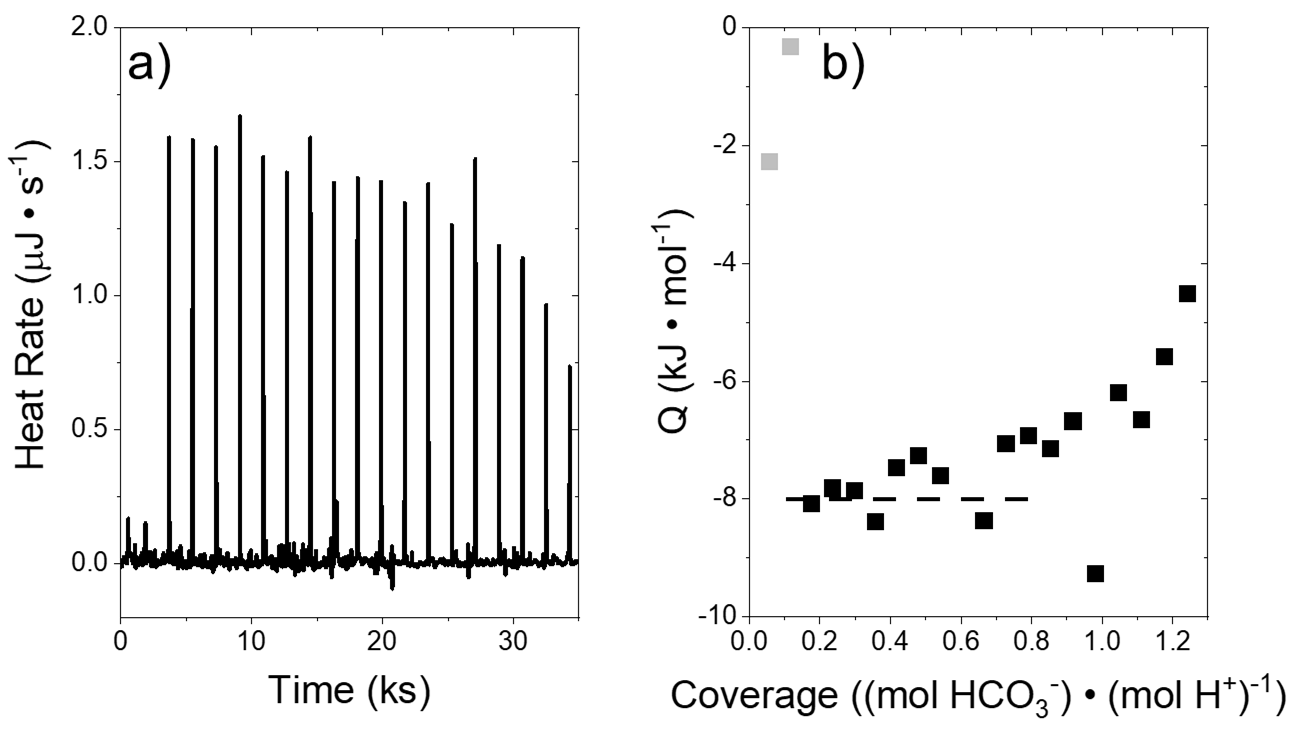


**Figure S17.** a) ITC thermogram from the acid-base titration of HNO_3_ (0.0005 M in 0.1 M NaCl) with NaHCO_3_ (0.0052 M in 0.1 M NaCl), b) the corresponding heats released as a function of bicarbonate ion coverage.

The ITC thermogram and heat release plots for all ITC experiments used to calculate the adsorption enthalpies of C_3_H_5_ClO and CH_3_OH into M-BEA are shown below. The CH_3_OH adsorption experiments were performed at higher titrant concentrations (and thus higher coverages) because CH_3_OH adsorbs weakly and higher concentrations are needed to observe sufficiently large peaks in the thermogram. Early injections often show lower heat released than expected due to the evaporation of liquid from the syringe needle. Although less common, some of the experiments below show outlier points at later injections, which may result from an inconsistent volume or epoxide concentration in those injections.


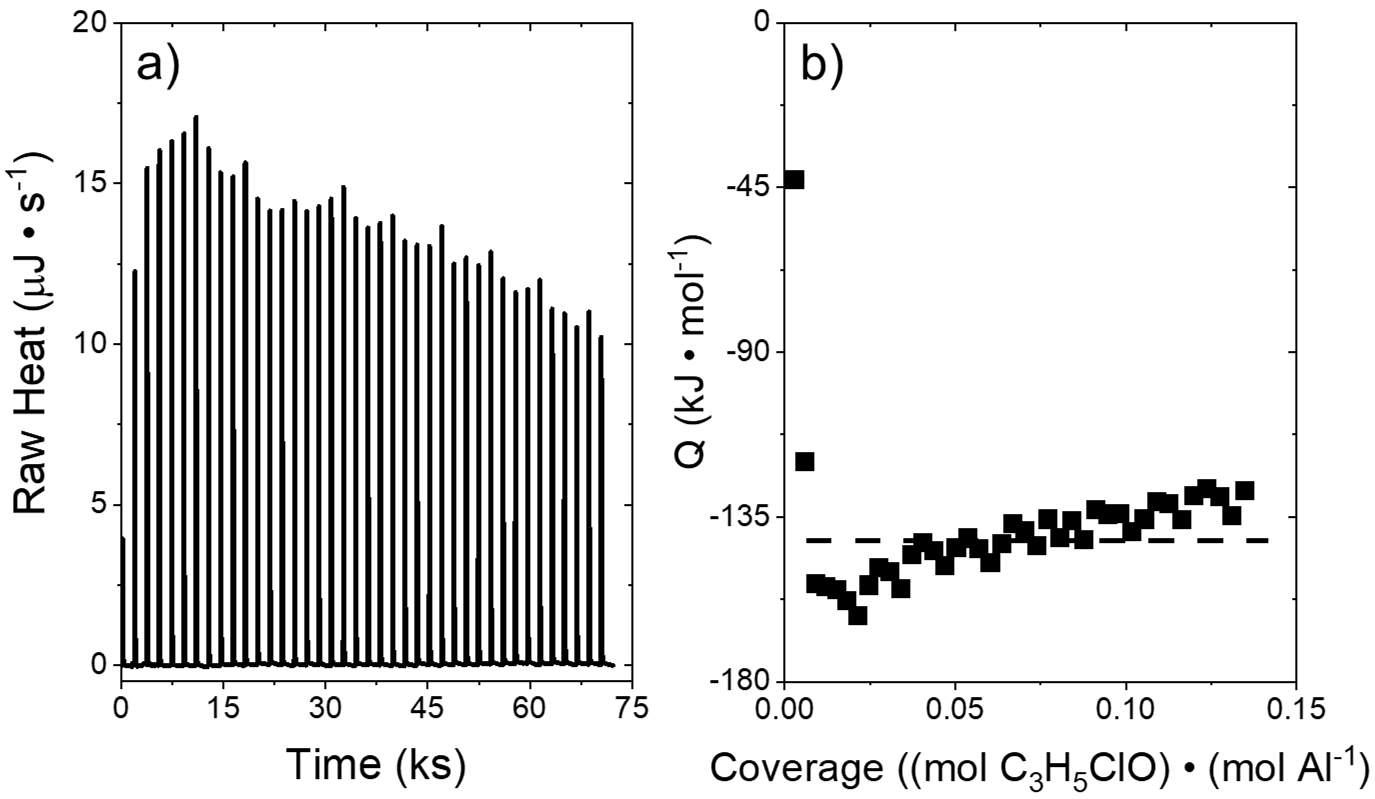


**Figure S18.** a) ITC thermogram from the titration of Al-BEA with C_3_H_5_ClO (0.005 M C_3_H_5_ClO in CH_3_CN, 308 K, 1 μL per injection), b) the corresponding heats released as a function of titrant injected.


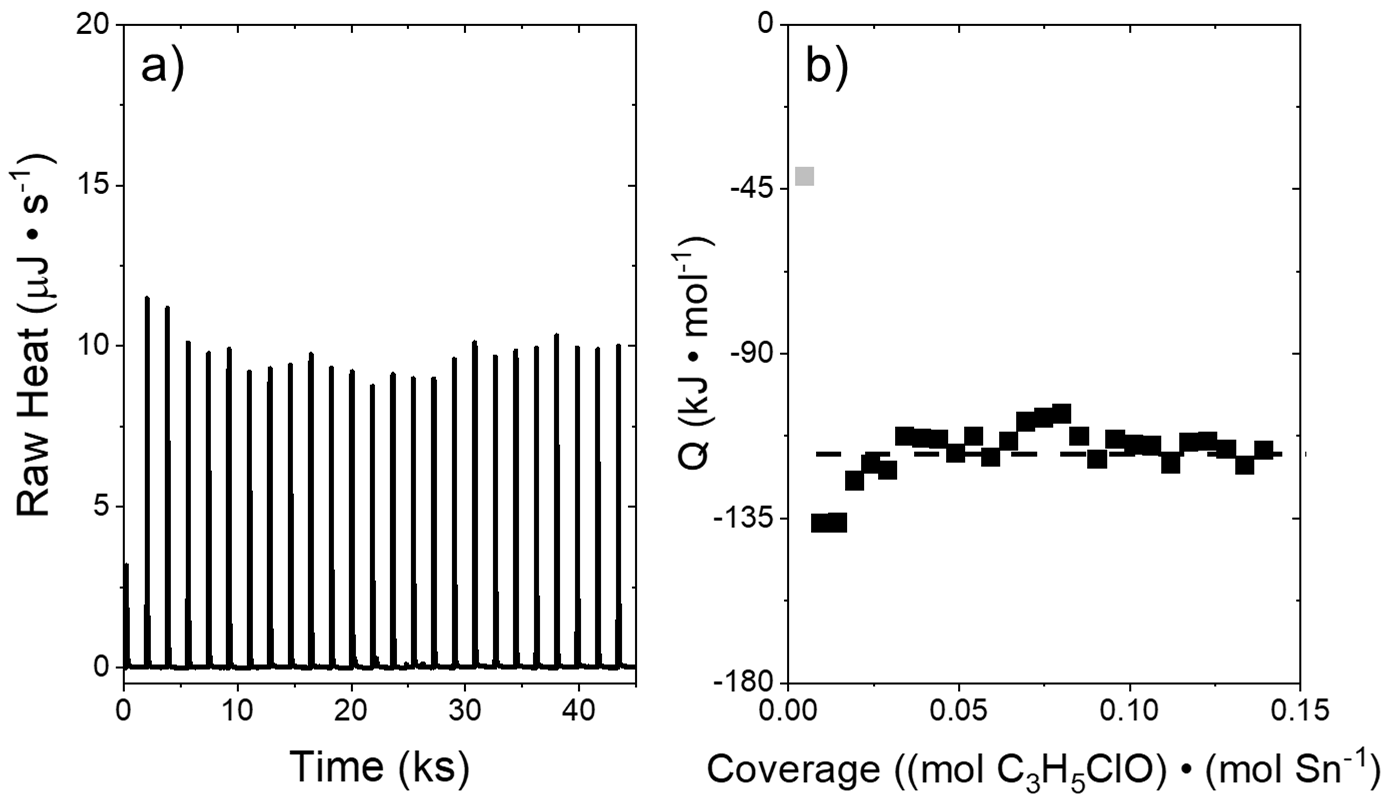


**Figure S19.** a) ITC thermogram from the titration of Sn-BEA with C_3_H_5_ClO (0.005 M C_3_H_5_ClO in CH_3_CN, 308 K, 1 μL per injection), b) the corresponding heats released as a function of titrant injected.


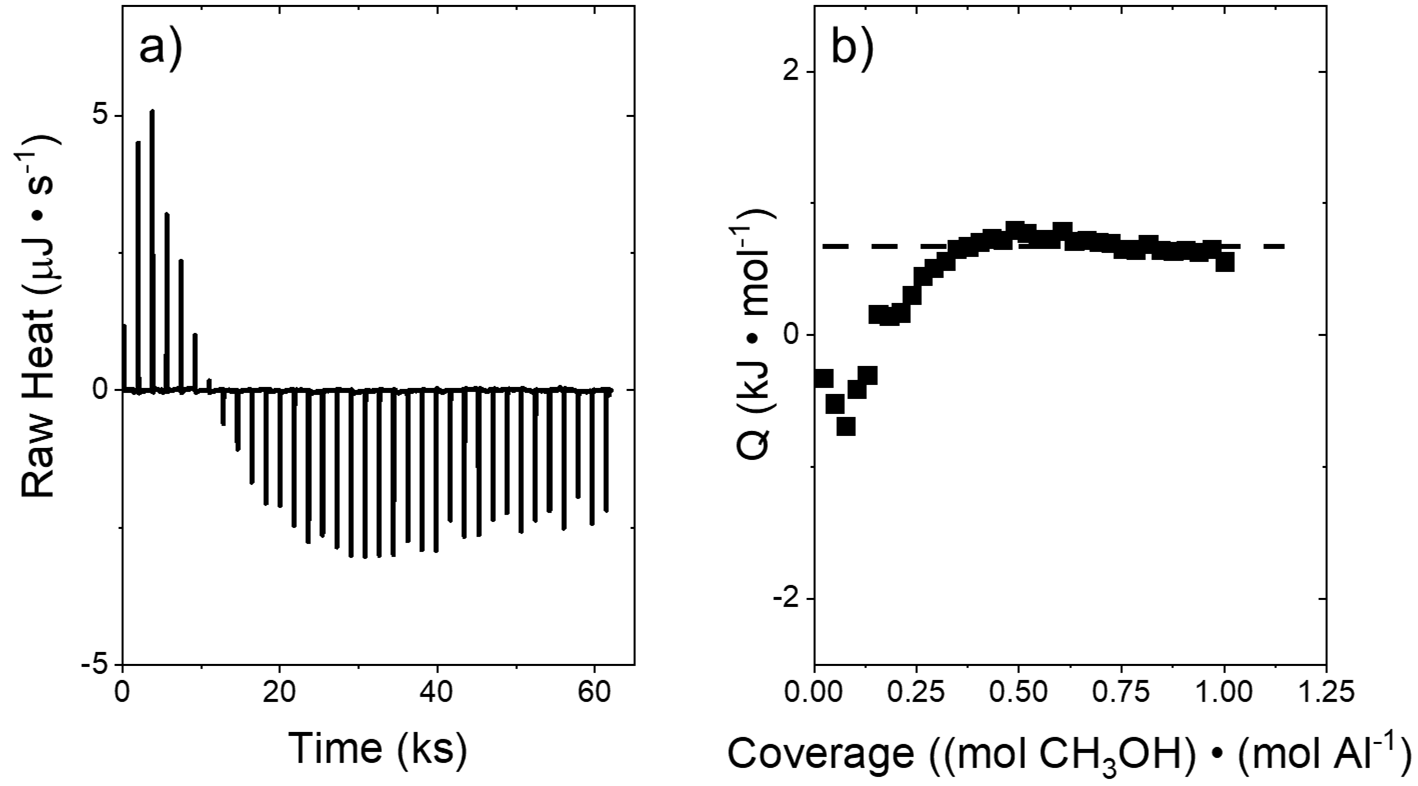


**Figure S20.** a) ITC thermogram from the titration of Al-BEA with CH_3_OH (0.1 M CH_3_OH in CH_3_CN, 308 K, 1 μL per injection), b) the corresponding heats released as a function of titrant injected.


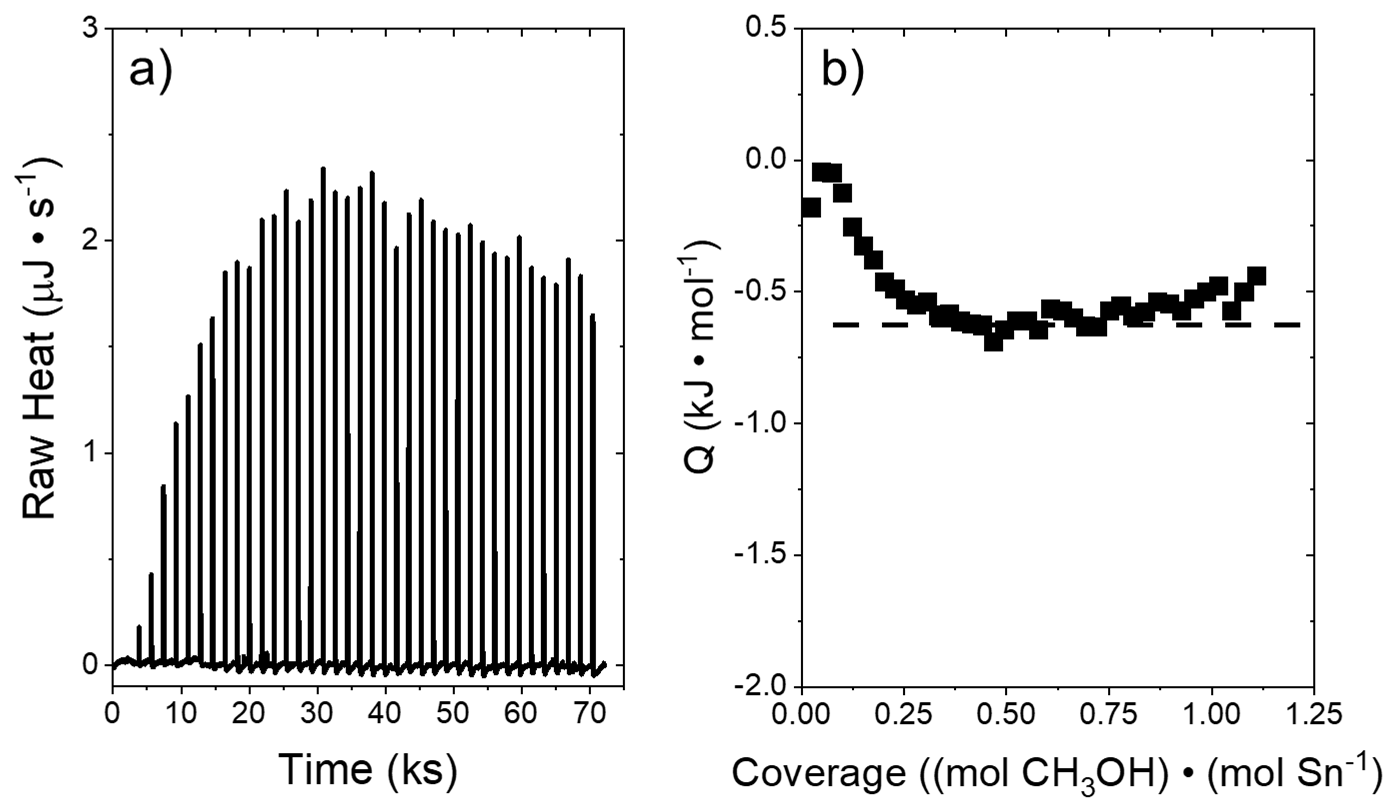


**Figure S21.** a) ITC thermogram from the titration of Sn-BEA with CH_3_OH (0.1 M CH_3_OH in CH_3_CN, 308 K, 1 μL per injection), b) the corresponding heats released as a function of titrant injected.

| Catalyst | ${\Delta H}_{ads,CH_{3}OH}$ (kJ mol^-1^) | ${\Delta H}_{ads,C_{3}H_{5}ClO}$ (kJ mol^-1^) |
| --- | --- | --- |
| Al-BEA | 1.0 ± 0.4 | -141.7 ± 8.9 |
| Sn-BEA | -0.6 ± 0.2 | -116.9 ± 7.0 |

**Table S7**. Adsorption enthalpies for CH_3_OH and C_3_H_5_ClO calculated from ITC thermograms in Figures S18 to S21.

The adsorption enthalpies in Table S7 were calculated by fitting a line through the injections where the heat released was approximately constant. C_3_H_5_ClO shows significantly more exothermic adsorption enthalpies than CH_3_OH over both Al-BEA and Sn-BEA, which may explain why adsorbed C_3_H_5_ClO dominates on active sites even in excess of CH_3_OH ([CH_3_OH]:[C_3_H_5_ClO] < 15, see Figure 1).

CH_3_OH shows more exothermic adsorption to Sn-BEA than Al-BEA. This suggests that Sn sites activate CH_3_OH more strongly than the Brønsted acid sites in Al-BEA, which may contribute to the greater reaction rates observed over Sn-BEA.

In contrast, C_3_H_5_ClO adsorbs more exothermically to Al-BEA than Sn-BEA. Figure S23 (*vide infra*) suggests that Al-BEA forms ring-opened carbocations from C_3_H_5_ClO more readily than Sn-BEA. The formation of these carbocations likely contributes to the more exothermic adsorption of C_3_H_5_ClO over Al-BEA.

**S12. Supplemental ^13^C NMR Plots and Analysis**

**S12.1. ^13^C NMR Shifts and Discussion of Peak Convolution**

| **Molecule** | **ChemDraw-Predicted ^13^C Shifts (ppm)** | **DFT-Predicted ^13^C Shifts (ppm)** | **Experimental ^13^C Shifts (ppm)** |
| --- | --- | --- | --- |
| 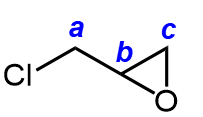 Epichlorohydrin | a = 45.4  b = 51.4  c = 46.9 | COSMO (in Methanol)  a = 51.77  b = 51.85  c = 47.56 | 1 M ^13^C ECH in Acetonitrile  a = 47.67  b = 52.44  c = 47.67 |
| 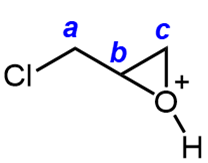  Protonated Epichlorohydrin | N/A  (unable to predict) | COSMO (in Methanol)  a = 43.0  b = 82.9  c = 74.6 | N/A |
| 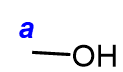    Methanol | a = 49.7 | COSMO (in Methanol)  a = 49.6 | 1 M ^13^CH_3_OH in Acetonitrile  a = 49.6 |
| 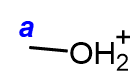  Protonated Methanol | a = 72.1 | COSMO (in Methanol)  a = 68.3 | N/A |
| 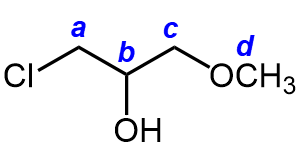Terminal ether | a = 46.2  b = 70.6 ± 3.7  c = 76.2 ± 2.5  d = 59.0 ± 1.7 | COSMO (in Methanol)  a = 54.5  b = 77.9  c = 75.9  d = 55.8 | 3.1 M C_4_H_9_ClO_2_ in CD_3_CN  a = 46.5  b = 69.8  c = 73.6  d = 58.5 |
| 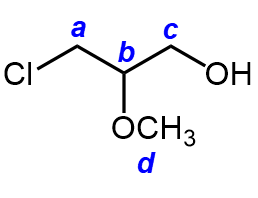Terminal alcohol | a = 44.9 ± 4.8  b = 85.3 ± 3.7  c = 66.9 ± 3.1  d = 58.4 ± 2.6 | COSMO (in Methanol)  a = 52.3  b = 85.7  c = 68.5  d = 59.1 | N/A  (no standard available) |
| 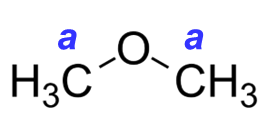  Dimethyl ether (DME) | a = 59.3 | COSMO (in Methanol)  a = 57.5 | N/A  (not measured) |

**Table S8**. Predicted ^13^C NMR peak shifts for reactants, primary products, and intermediates from the ring-opening of C_3_H_5_ClO with CH_3_OH. These peak shifts were predicted by averaging values and errors obtained from ACD/Labs (v2024) and ChemDraw (version 23.0.1), which were further cross-referenced with reported shifts available in the literature.

| **Entry** | **Binding Structure** | **DFT-Predicted ^13^C Shifts (ppm)** |
| --- | --- | --- |
| 1 | 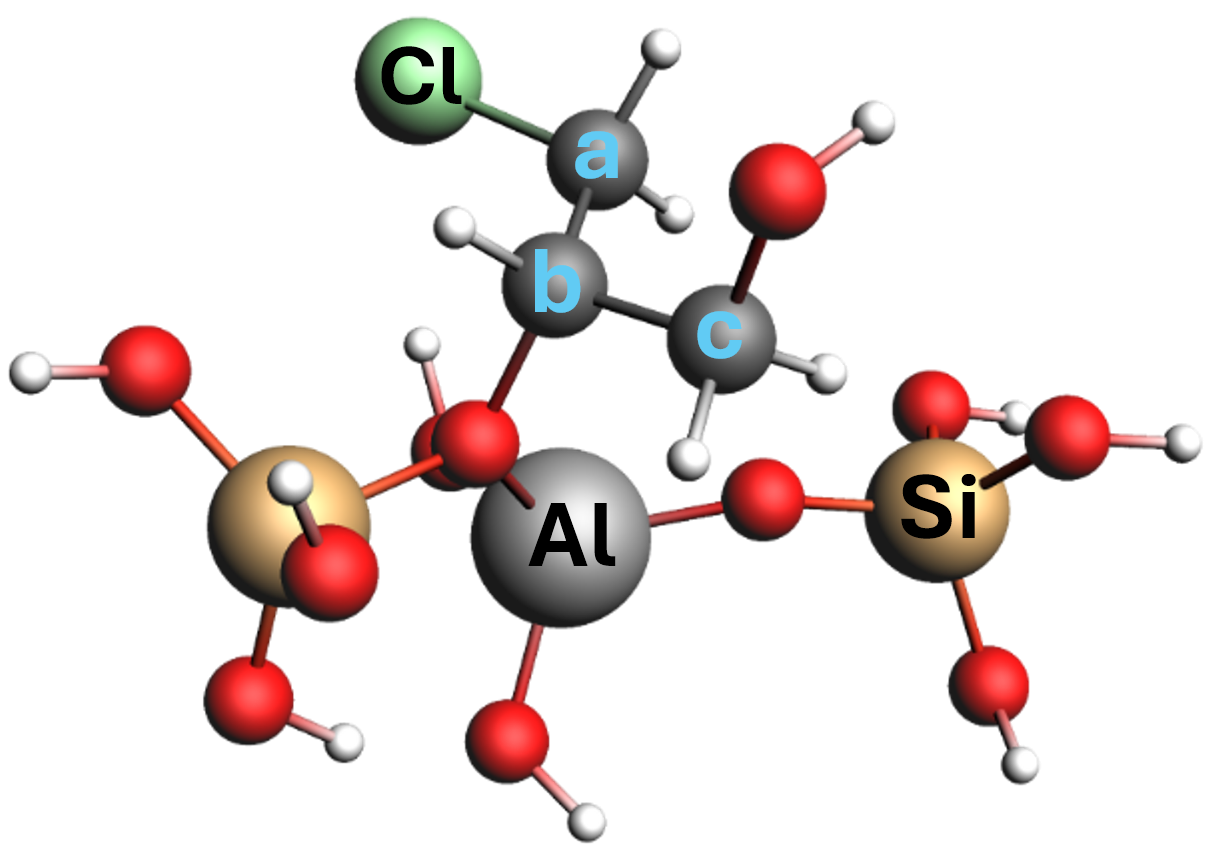  Secondary carbocation (derived from C_3_H_5_ClO)  (bound to Al-BEA) | COSMO (in Methanol)  a = 49.6  b = 89.0  c= 63.0 |
| 2 | 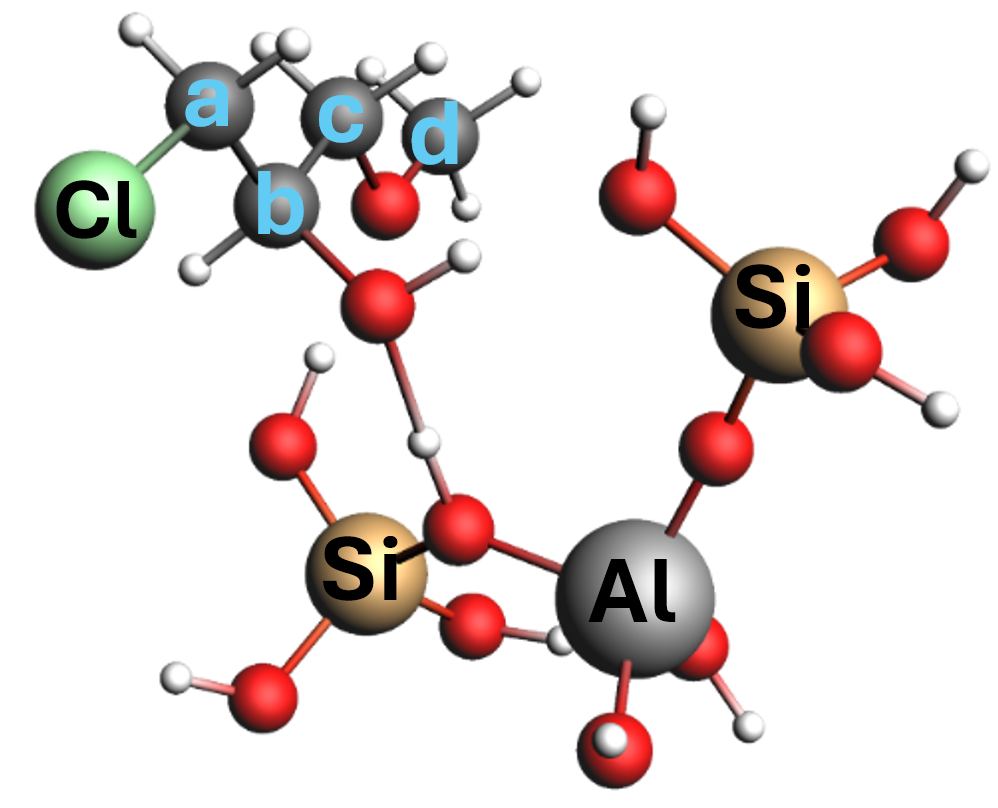  Terminal ether bound at Brønsted acid site in Al-BEA | COSMO(Methanol)  a=51.5  b=77.2  c=73.6  d=55.8 |
| 3 | 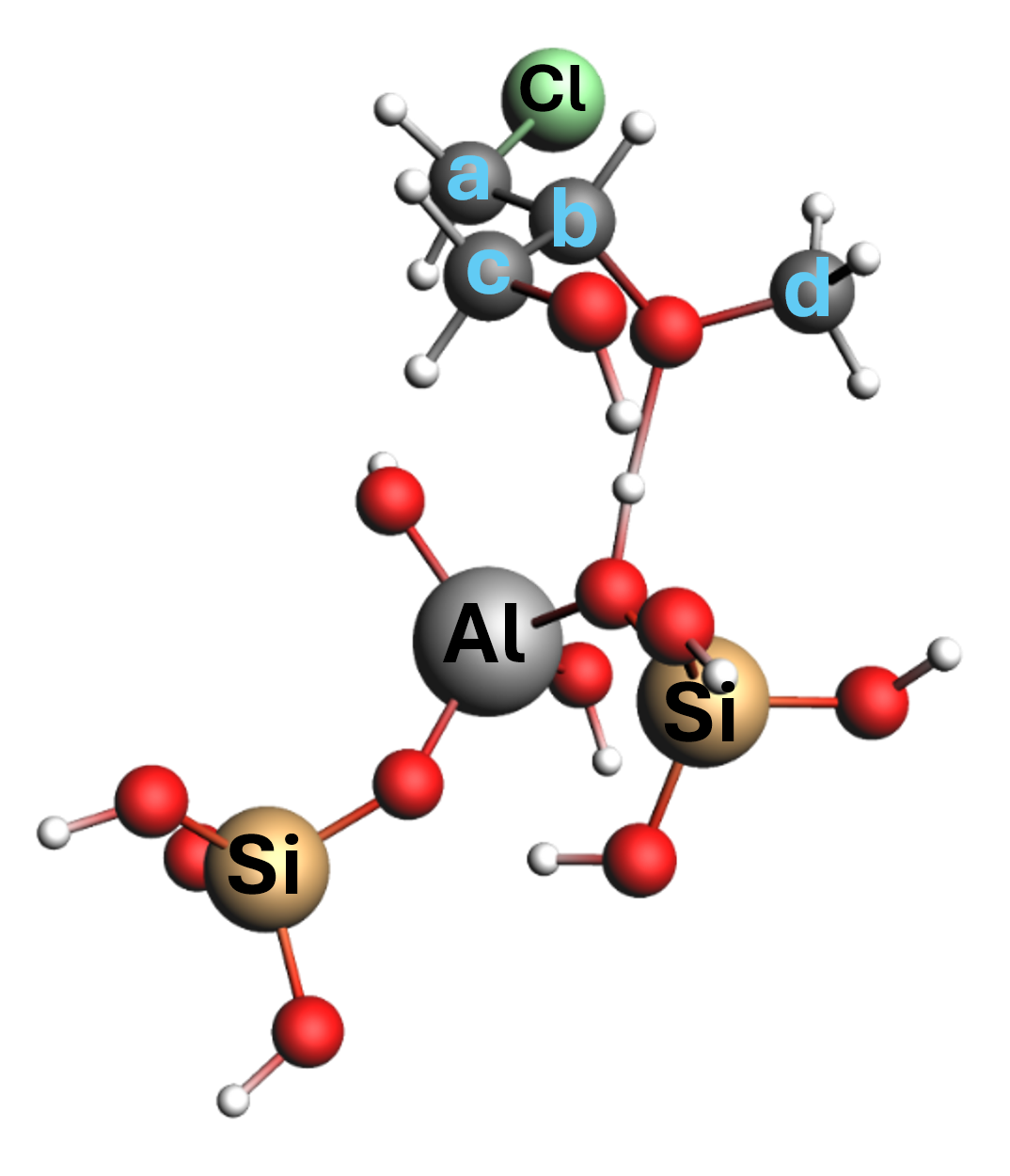    Terminal alcohol bound at Brønsted acid site, Al-BEA | COSMO(Methanol)  a=50.9  b=91.6  c=61.8  d=59.8 |
| 4 | 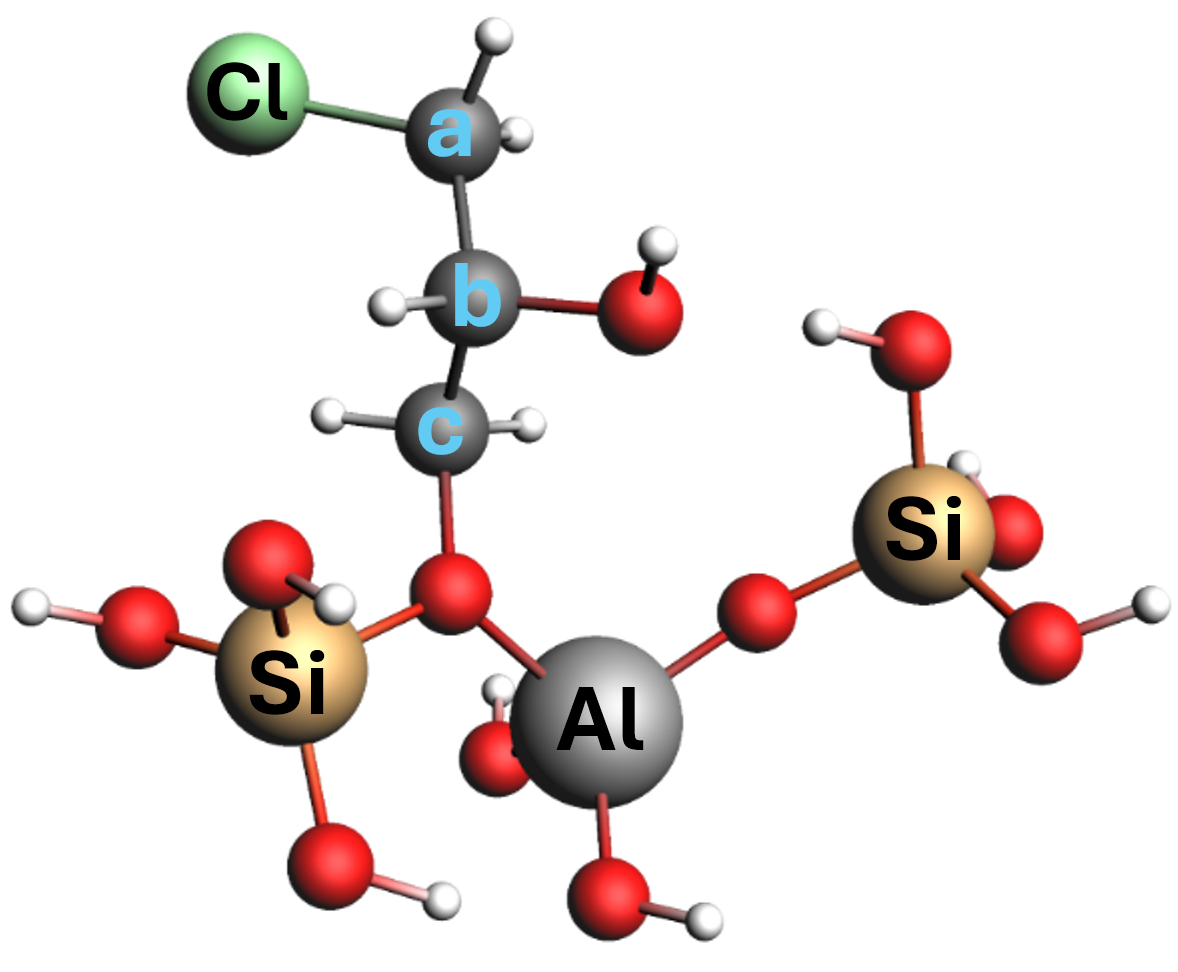  Primary carbocation (derived from C_3_H_5_ClO)  (bound to Brønsted acid site in Al-BEA) | COSMO(Methanol)  a=47.0  b=74.6  c=71.1 |

| 5 | 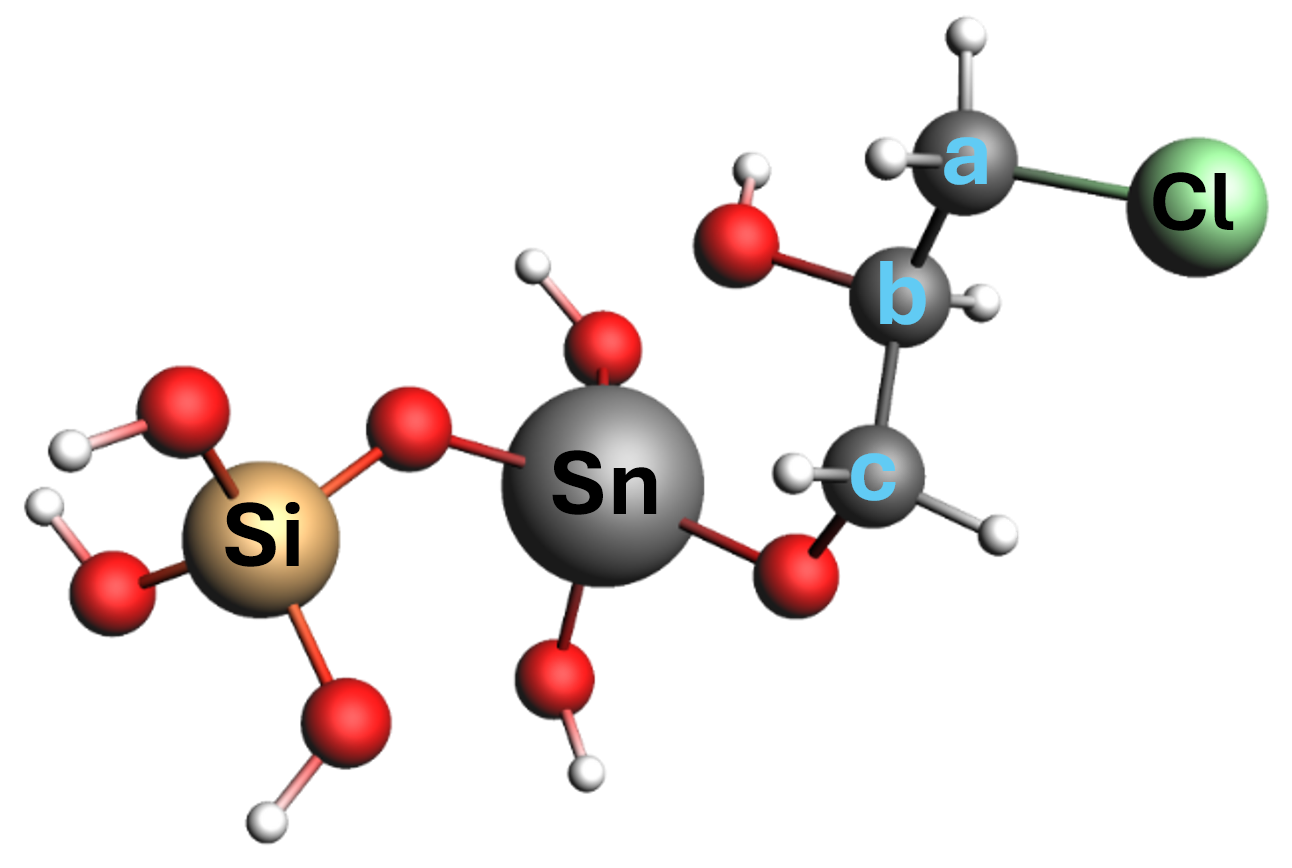  Primary carbocation bound to Sn-BEA | COSMO (Methanol)  a=49.3  b=72.5  c=67.3 |
| --- | --- | --- |
| 6 | 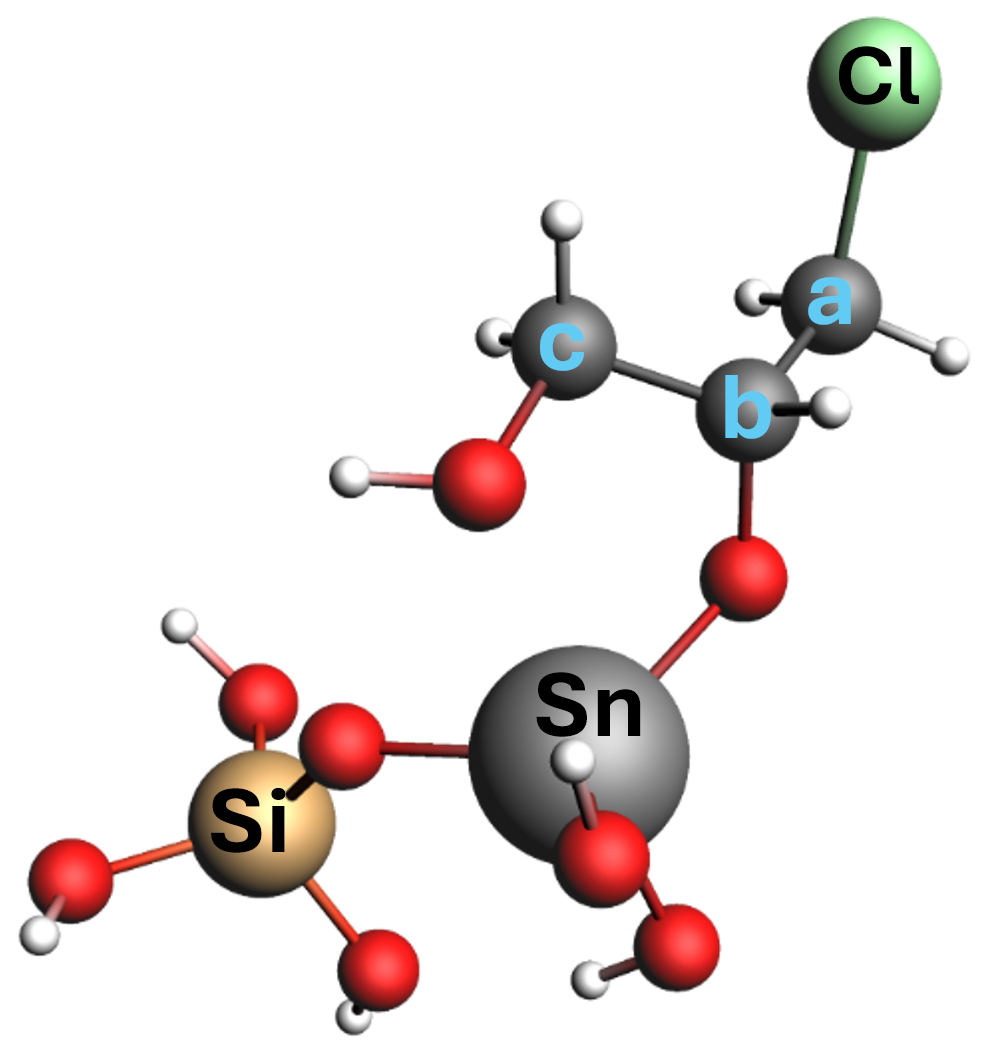  Secondary carbocation bound to Sn-BEA | COSMO (Methanol)  a=47.5  b=76.2  c=64.7 |
| 7 | 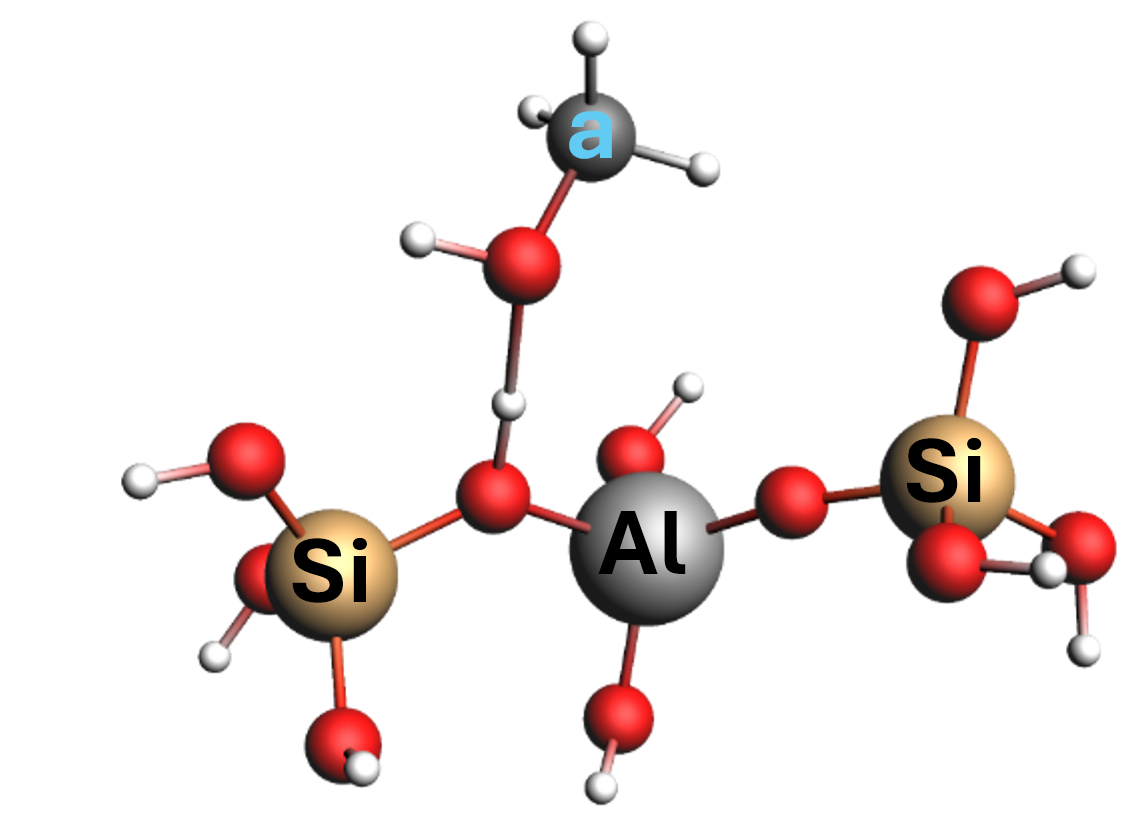  Methanol bound at Brønsted acid site in Al-BEA | COSMO(Methanol)  a=49.6  O (methanol)-H (BAS) bond length = 1.519 Å  H-O (BAS) bond length = 1.047 Å |
| 8 | 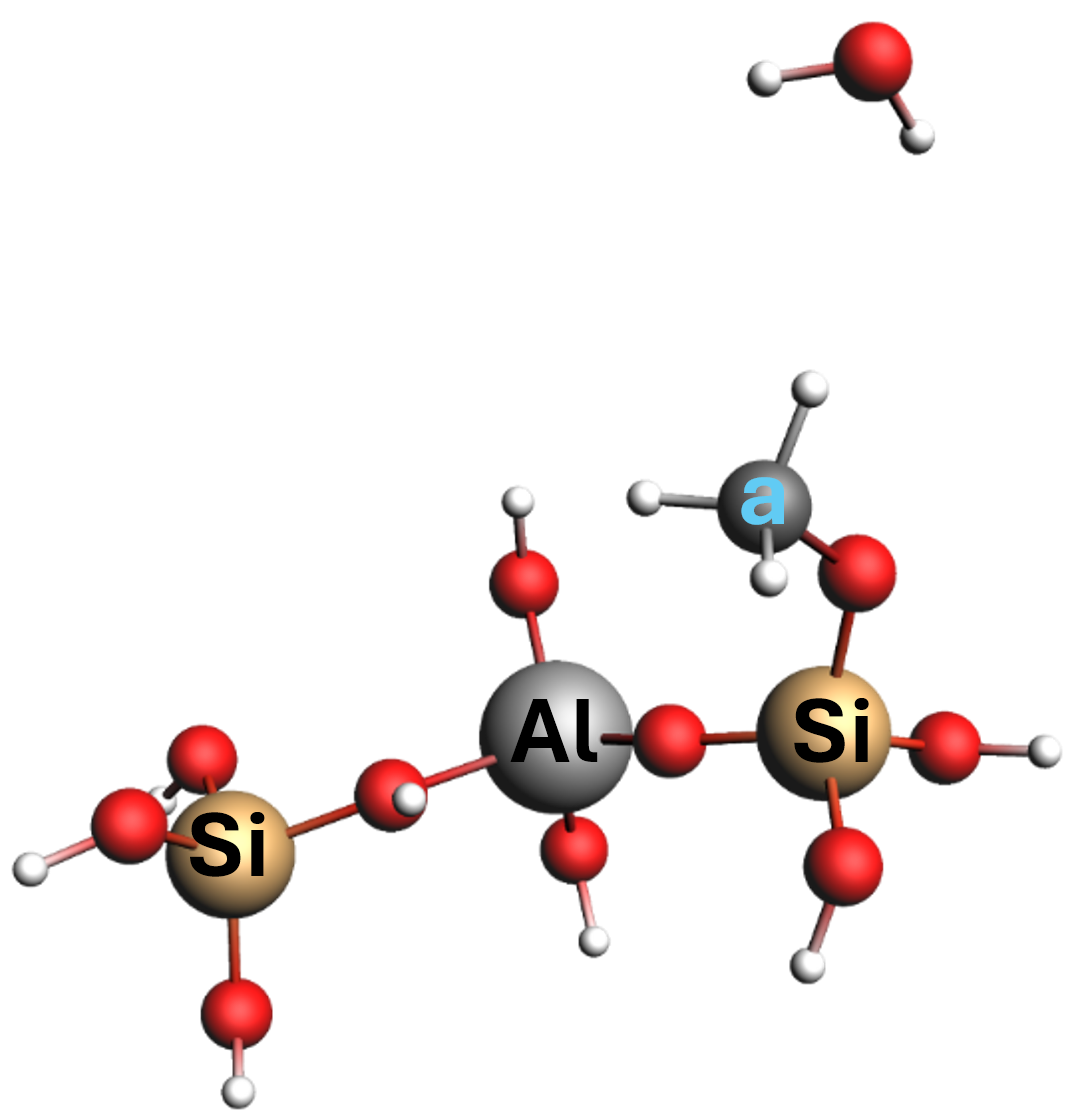  Methoxy bound at Si adjacent to Brønsted acid site in Al-BEA (with eliminated H_2_O from CH_3_OH) | COSMO(Methanol)  a=48.5 |
| 9 | 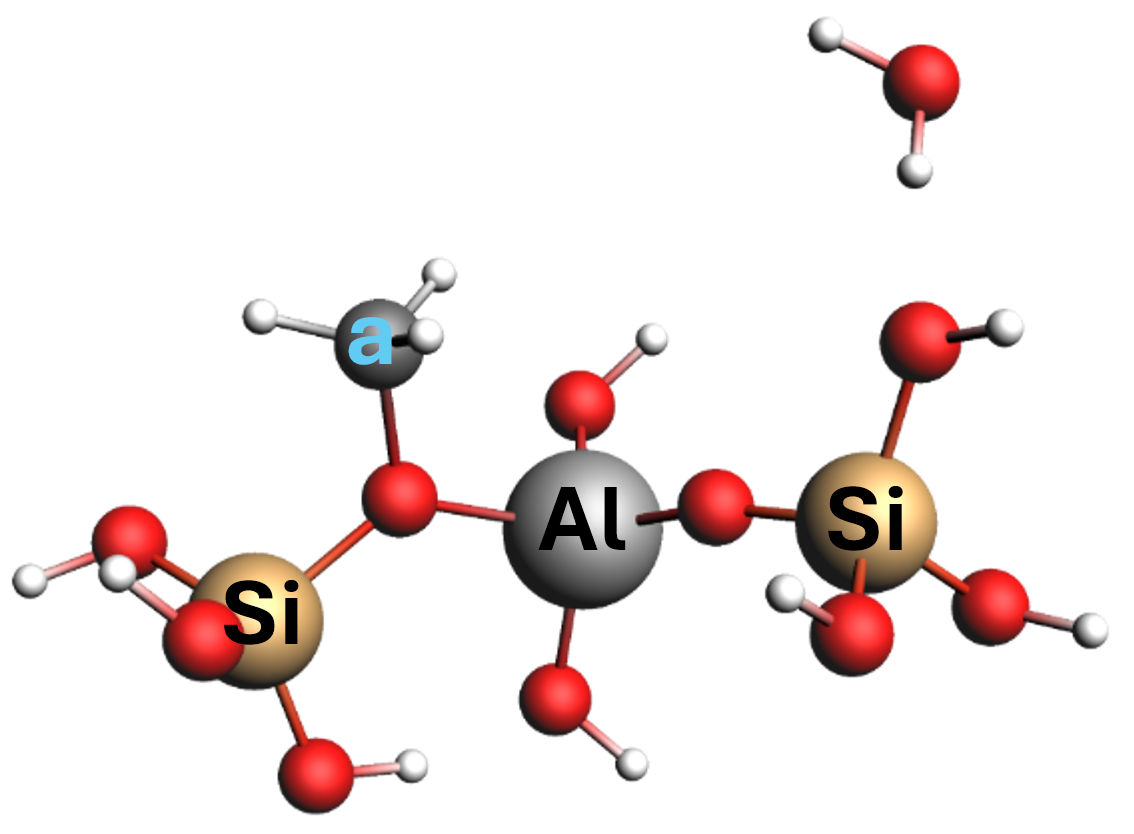  Methoxy bound at Brønsted acid site in Al-BEA  (with eliminated H_2_O molecule from CH_3_OH) | COSMO (Methanol)  a=54.6 |
| 10 | 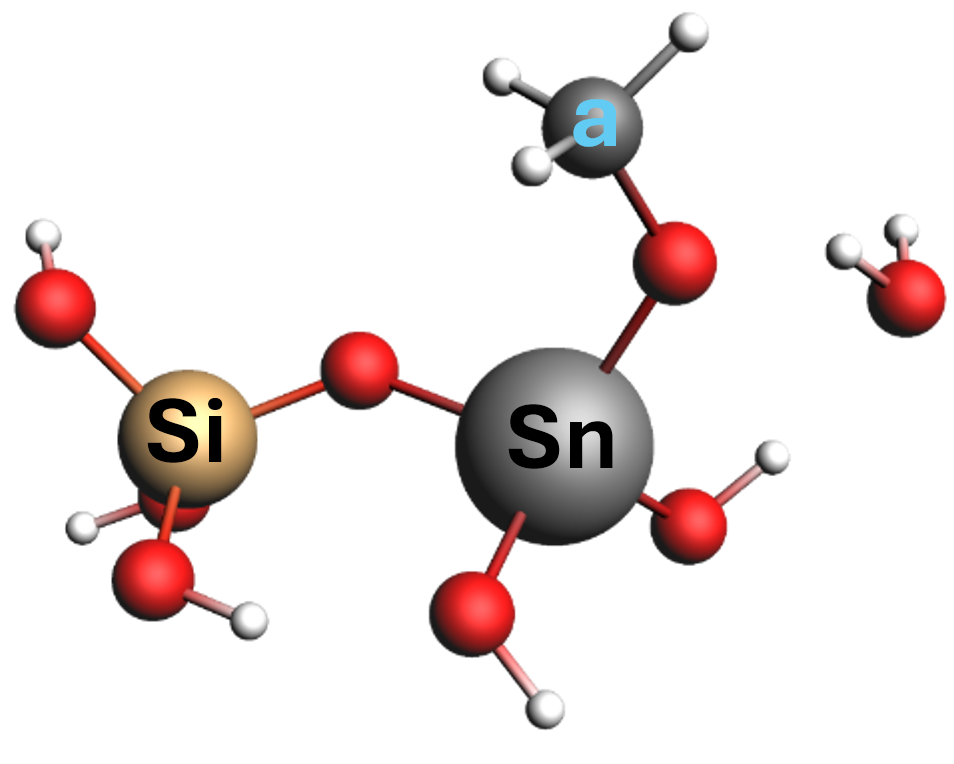  Methoxy bound to Sn site in Sn-BEA  (with eliminated H_2_O from CH_3_OH) | COSMO(methanol)  a=55.2 |
| 11 | 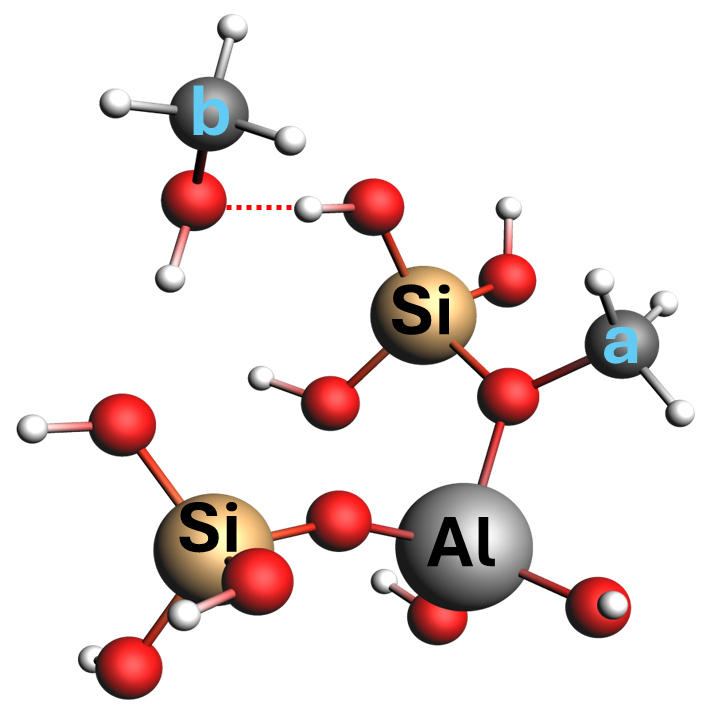  Methoxy bound at Brønsted acid site in Al-BEA, with methanol bound at adjacent Si-OH group | COSMO (Methanol)  a=52.0  b=52.3 |
| 12 | 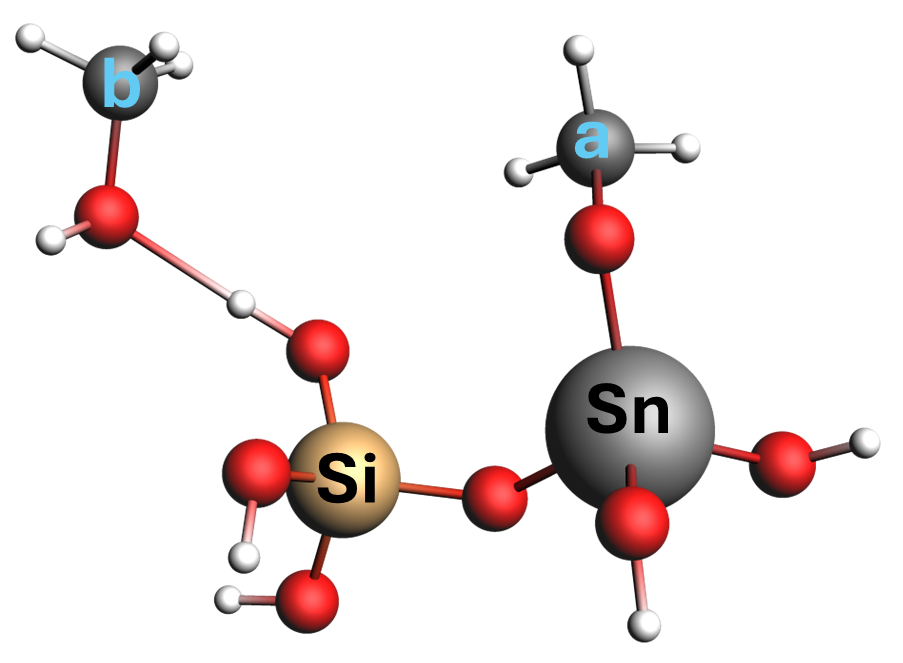  Methoxy bound at Sn-OH acid site in Sn-BEA, with methanol bound at adjacent Si-OH group | COSMO (Methanol)  a=53.8  b=49.9 |
| 13 | 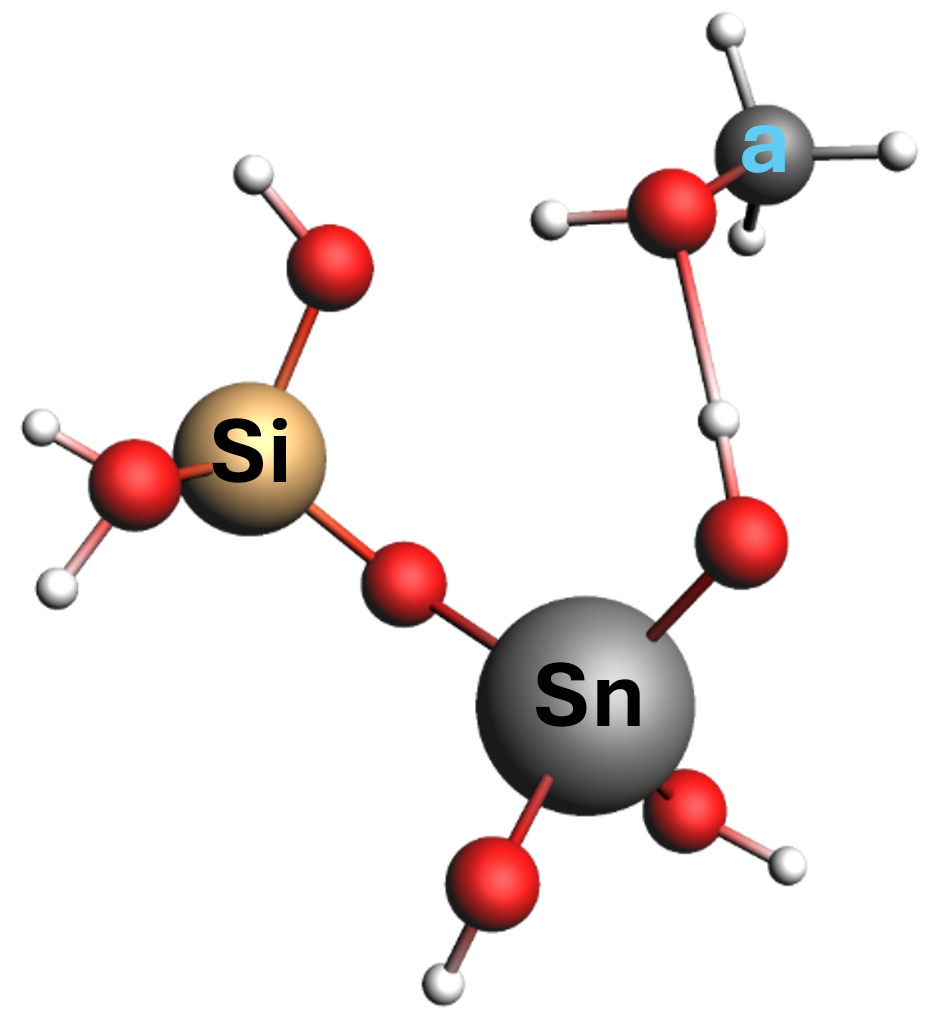  Methanol bound to -H at open site (Sn-OH) in Sn-BEA | COSMO(methanol)  a=47.9 |
| 14 | 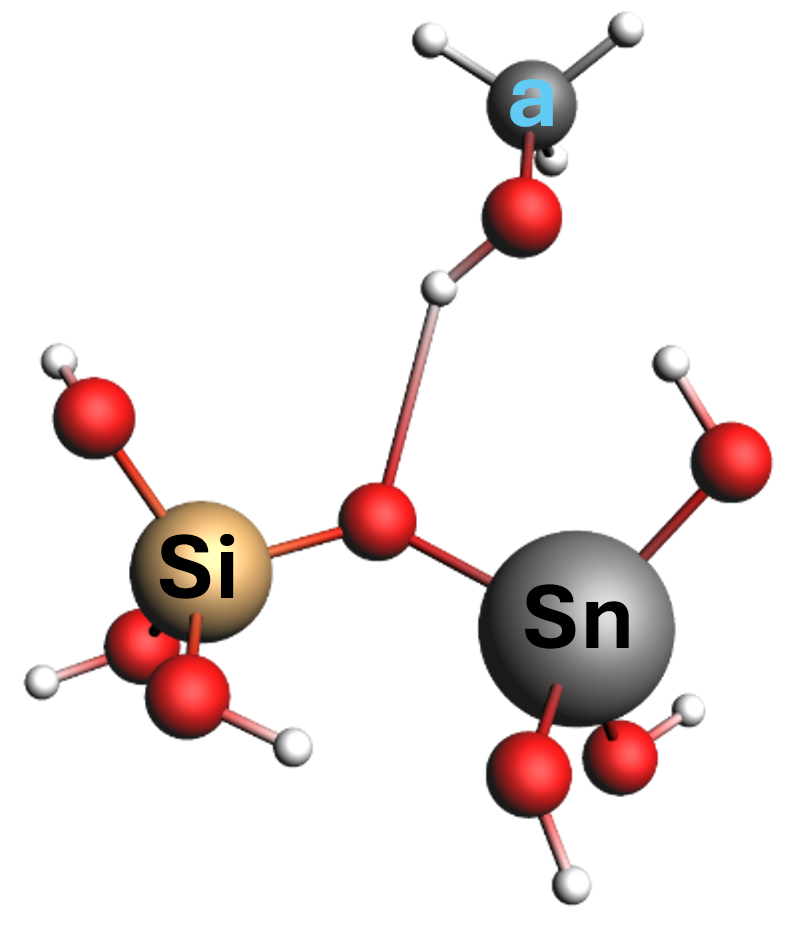Methanol bound to O in Sn-O-Si near open site (Sn-OH) in Sn-BEA | COSMO(methanol)  a=49.0 |
| 15 | 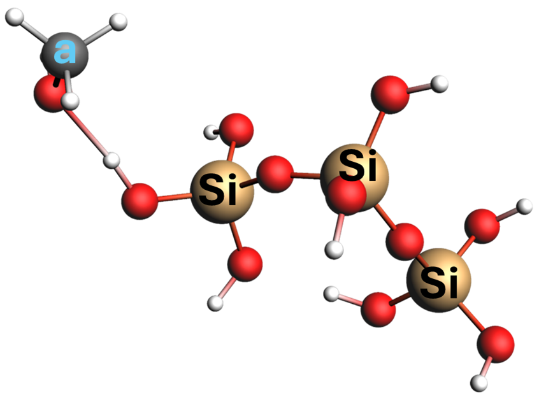Methanol bound at Si-OH | COSMO(methanol)  a=49.7  H (methanol)-O (Si-O-Si) bond length = 4.478 Å  Methanol unlikely to bind to this site  O (methanol)-H (Si-O-H) bond length = 1.714 Å  Methanol likely binds to this site |
| 16 | 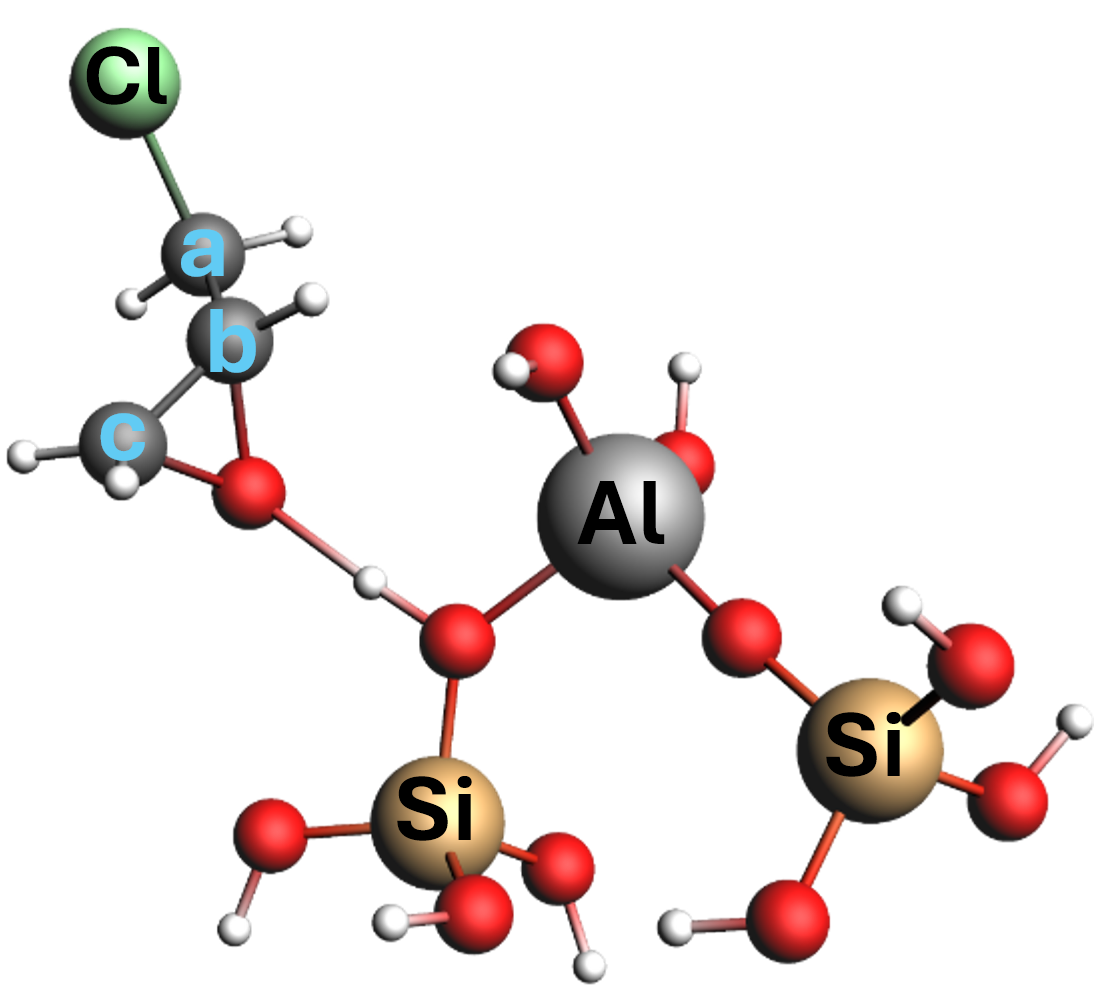Epichlorohydrin bound at Brønsted acid site in Al-BEA | COSMO(methanol)  a=49.5  b=55.7  c=51.1  BA-H to O of ECH bond length =1.567 Å  BA-H to O of BAS bond length =1.033 Å |
| 17 | 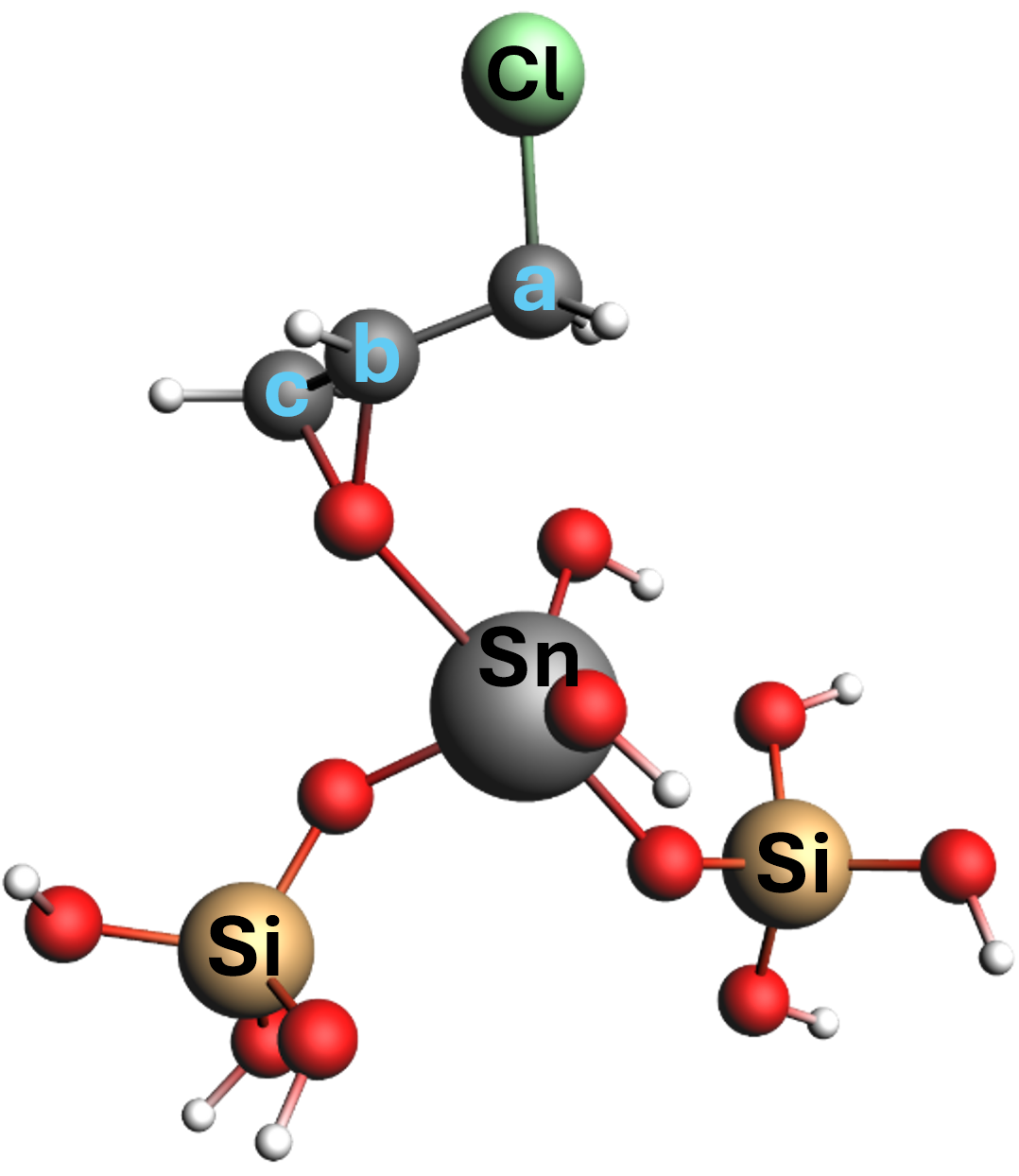  Epichlorohydrin bound at Sn site in Sn-BEA | COSMO(methanol)  a=48.5  b=58.9  c=53.5  O (ECH)-Sn bond length = 2.398 Å |
| 18 | Protonated CH_3_OH at Brønsted acid site, Al-BEA 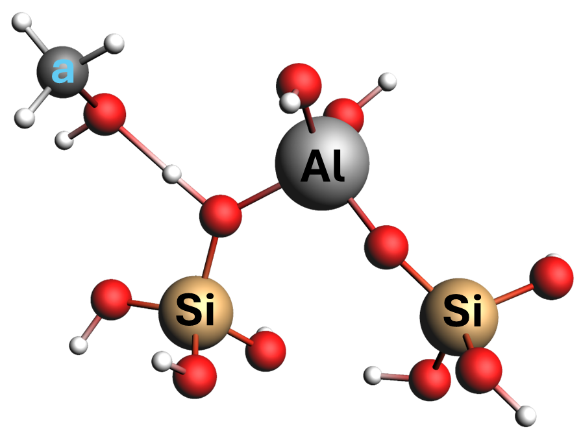 | COSMO(methanol)  a=50.2  O (methanol)-H (BAS) bond length = 1.492 Å  H-O (BAS) bond length = 1.055 Å  *Note that the predicted structure for protonated CH_3_OH has a nearly identical structure to un-protonated CH_3_OH bound to Brønsted acid sites, but with a slightly shorter bond between CH_3_OH and the proton and a 0.6 ppm ^13^C shift downfield. |
| 19 | 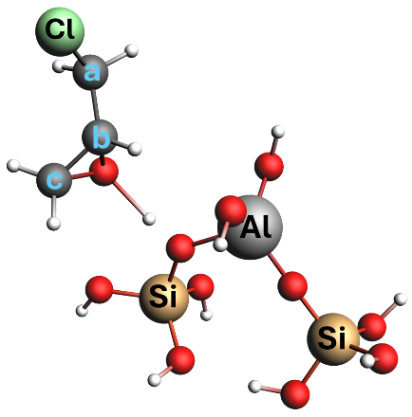  Protonated ECH at Brønsted acid site, Al-BEA | COSMO(methanol)  a=49.1  b=55.5  c=51.0  O (ECH)-H (BAS) bond length = 1.567 Å  H-O (BAS) bond length = 1.034 Å  *Note that the predicted structure for protonated ECH has nearly identical bond lengths and predicted peak shifts to un-protonated ECH bound to Brønsted acid sites. |
| 20 | 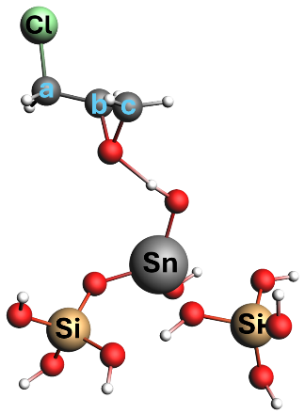  Protonated ECH at Sn-OH site, Sn-BEA | COSMO(methanol)  a=50.4  b=54.0  c=50.2  O (ECH)-H (Sn-OH) bond length = 1.697 Å  H-O (Sn-OH) bond length = 1.007 Å  *Note that the predicted structure for protonated ECH at Sn-OH has a significantly shorter bond length (1.697 Å) than for un-protonated ECH bound to Sn atoms (2.398 Å). |
| 21 | 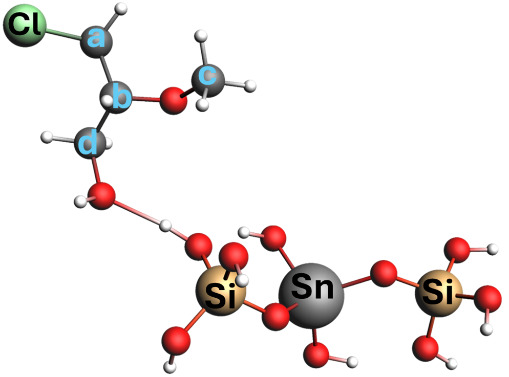  Terminal alcohol bound at Sn site, Sn-BEA | COSMO(methanol)  a=44.6  b=87.2  c=55.5  d=65.6  O (TA, -OCH_3_)-Sn bond length = 6.202 Å  TA unlikely to bind to this site  O (TA, -OH)-H(Si-O-H) bond length = 1.732 Å  TA likely binds to this site |
| 22 | 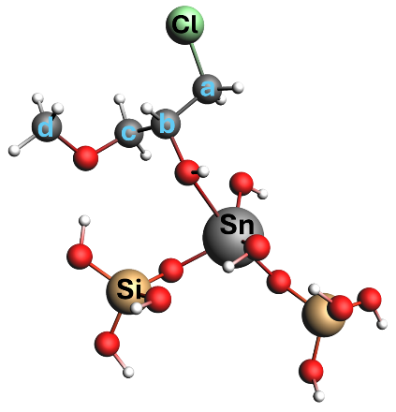  Terminal ether bound at Sn site, Sn-BEA | COSMO(methanol)  a=47.3  b=76.5  c=71.6  d=54.2  O (TE)-Sn bond length = 2.481 Å |
| 23 | 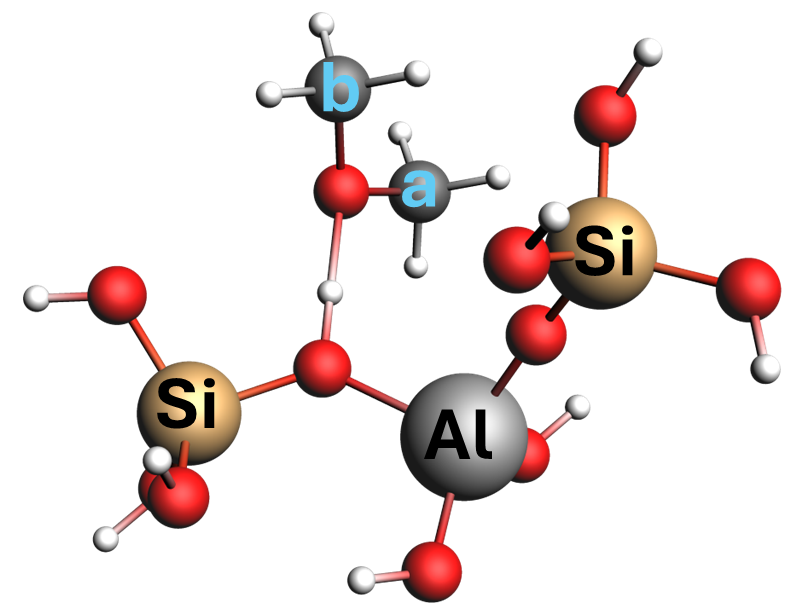  Dimethyl ether bound at Brønsted acid site, Al-BEA | COSMO(methanol)  a=51.4  b=52.4  O (DME)-H (BAS) bond length = 1.490 Å  H-O (BAS) bond length = 1.054 Å |
| 24 | 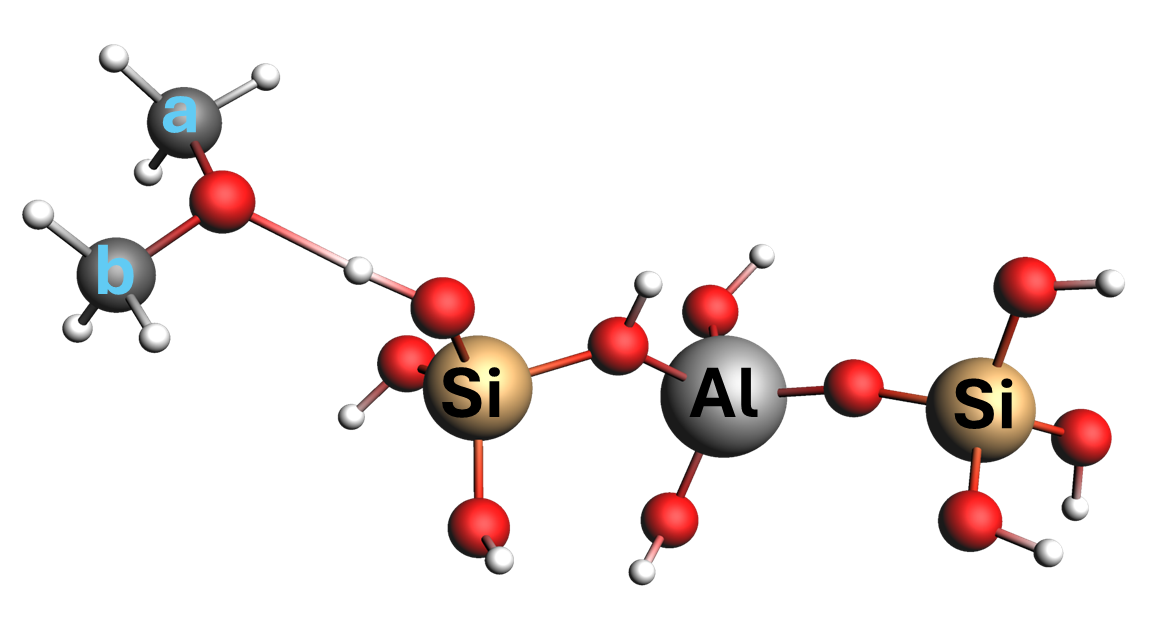  Dimethyl ether bound at Si-OH site, Al-BEA | COSMO(methanol)  a=57.1  b=57.0  O (DME)-H (Si-OH) bond length = 1.654 Å  H-O (Si-OH) bond length = 1.007 Å |
| 25 | 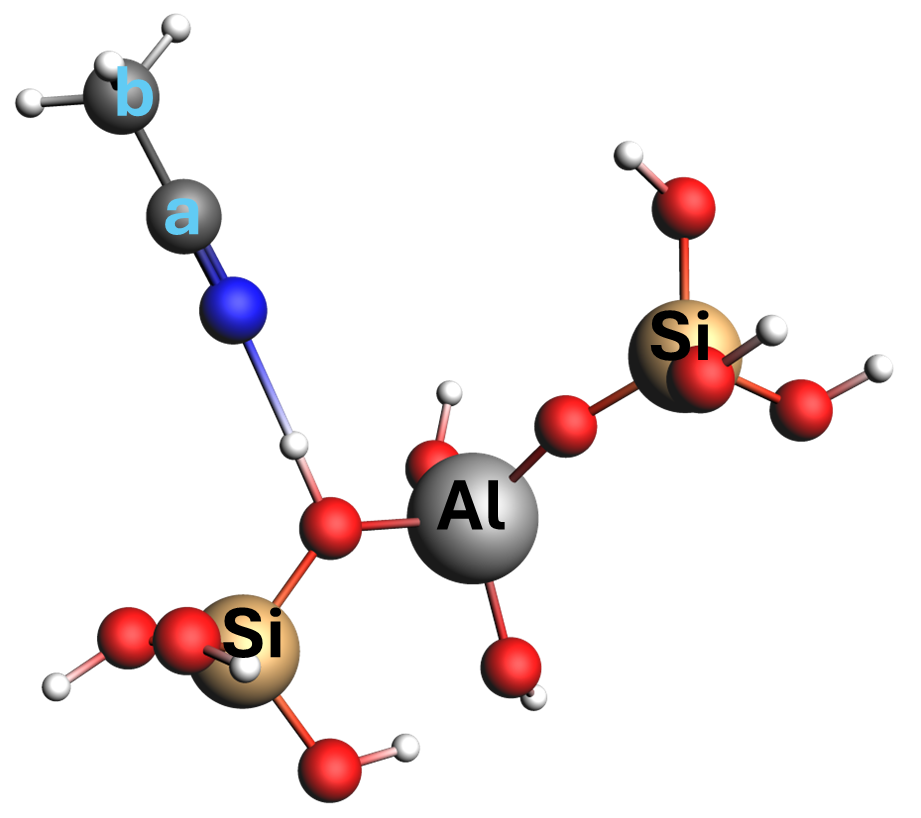  Acetontrile bound to Brønsted acid site, Al-BEA | COSMO(methanol)  a=113.8  b=-9.4  N (CH_3_CN)-H (BAS) bond length = 1.646 Å  H-O (BAS) bond length = 1.025 Å |
| 26 | 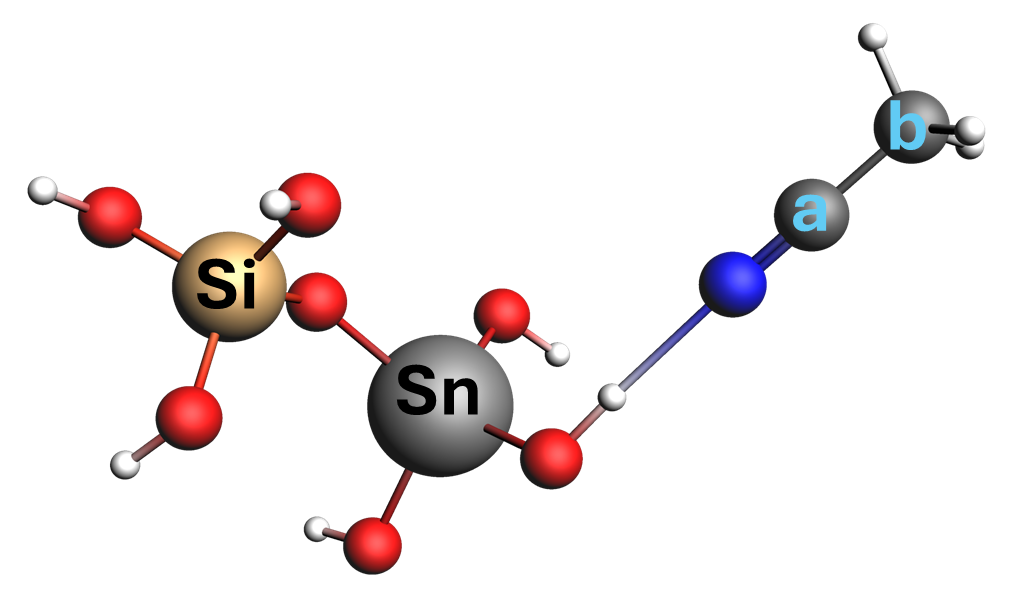  Acetontrile bound to Sn-OH site, S-BEA | COSMO(methanol)  a=115.6  b=-9.0  N (CH_3_CN)-H (Sn-OH) bond length = 1.872 Å  H-O (Sn-OH) bond length = 0.993 Å |

**Table S9**. Predicted ^13^C NMR peak shifts for reactants, primary products, and intermediates from the ring-opening of C_3_H_5_ClO with CH_3_OH, when adsorbed to Al-BEA and Sn-BEA acid sites. These peak shifts were predicted by the Amsterdam Density Functional package (ADF 2024).

The DFT calculations over Sn-BEA consider relativistic effects, while the calculations over Al-BEA do not. The heaviness of Sn atoms (118.71 amu) requires the consideration of relativistic effects for the DFT calculations to run properly.^81^ However, Al atoms (26.98 amu) do not suffer from such limitations, as the DFT software predicts approximately identical peak shifts with and without relativistic effects included. We therefore ran the DFT calculations of Al-BEA without relativistic effects for simplicity.

| **Molecule** | **NO COSMO** Shift (ppm) | **COSMO in Acetonitrile** Shift (ppm) | **COSMO in Methanol** Shift (ppm) |
| --- | --- | --- | --- |
| 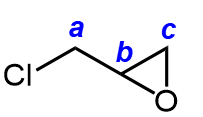Epichlorohydrin | a = 50.26  b = 50.62  c = 45.20 | a = 51.76  b = 51.84  c = 47.54 | a = 51.77  b = 51.85  c = 47.56 |
| 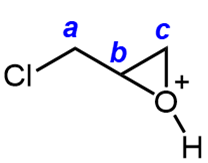  Protonated Epichlorohydrin | -- | a = 43.0  b = 82.8  c = 74.6  b-O bond: 1.591 Å  c-O bond: 1.545 Å  O-H bond: 0.986 Å | a = 43.0  b = 82.9  c = 74.6  b-O bond: 1.591 Å  c-O bond: 1.545 Å  O-H bond: 0.986 Å |
| 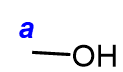    Methanol | -- | a = 49.56 | a = 49.6 |
| 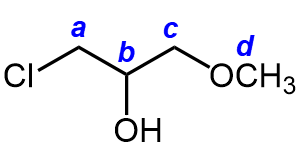Terminal ether | -- | a = 54.5  b = 77.9  c = 75.9  d = 55.8 | a = 54.5  b = 77.9  c = 75.9  d = 55.8 |
| 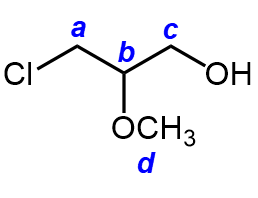Terminal alcohol | -- | a = 52.3  b = 85.7  c = 68.5  d = 59.1 | a = 52.3  b = 85.7  c = 68.5  d = 59.1 |
| 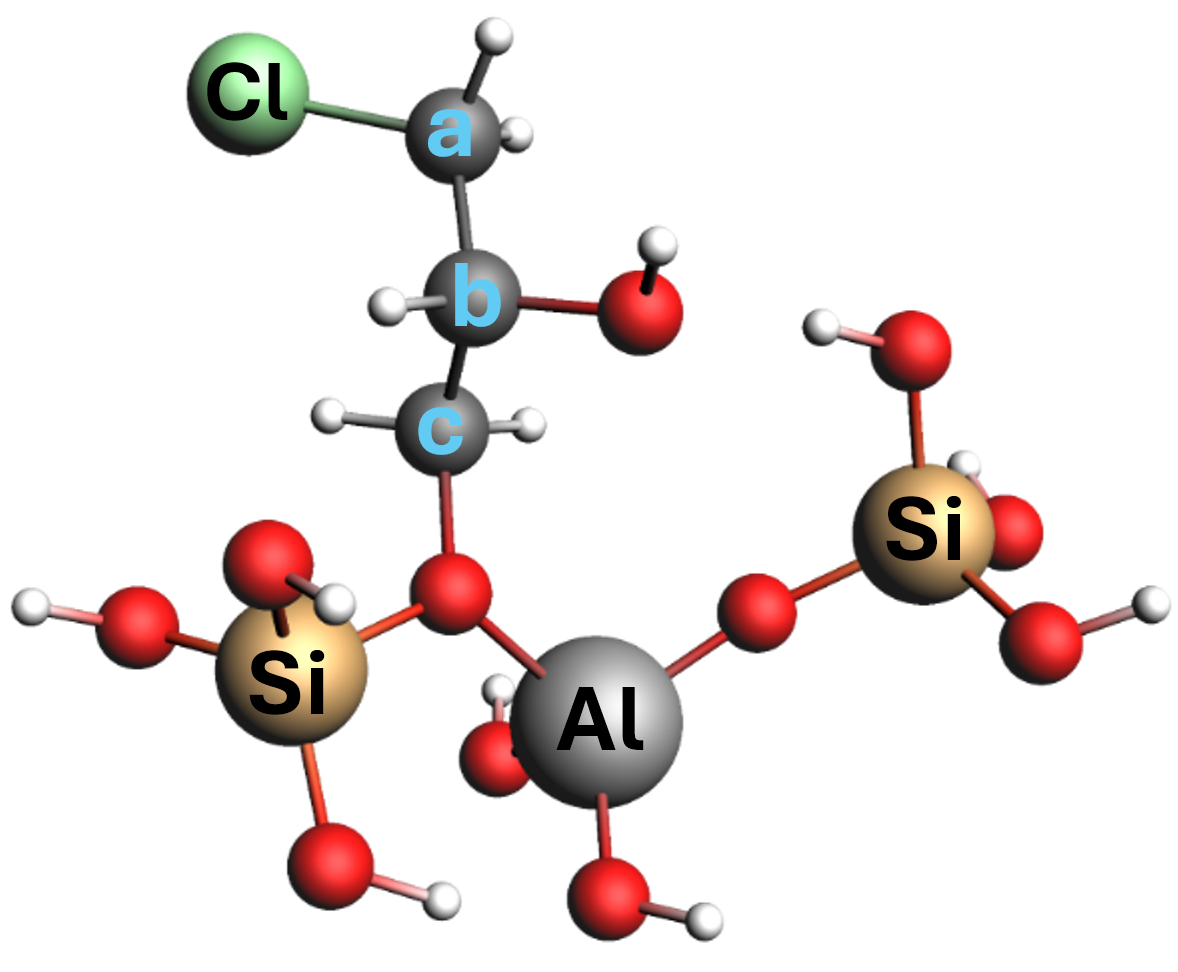  Primary carbocation (bound to Brønsted acid site, Al-BEA) | -- | a=46.8  b=75.1  c=71.2 | a=47.0  b=74.6  c=71.1 |
| 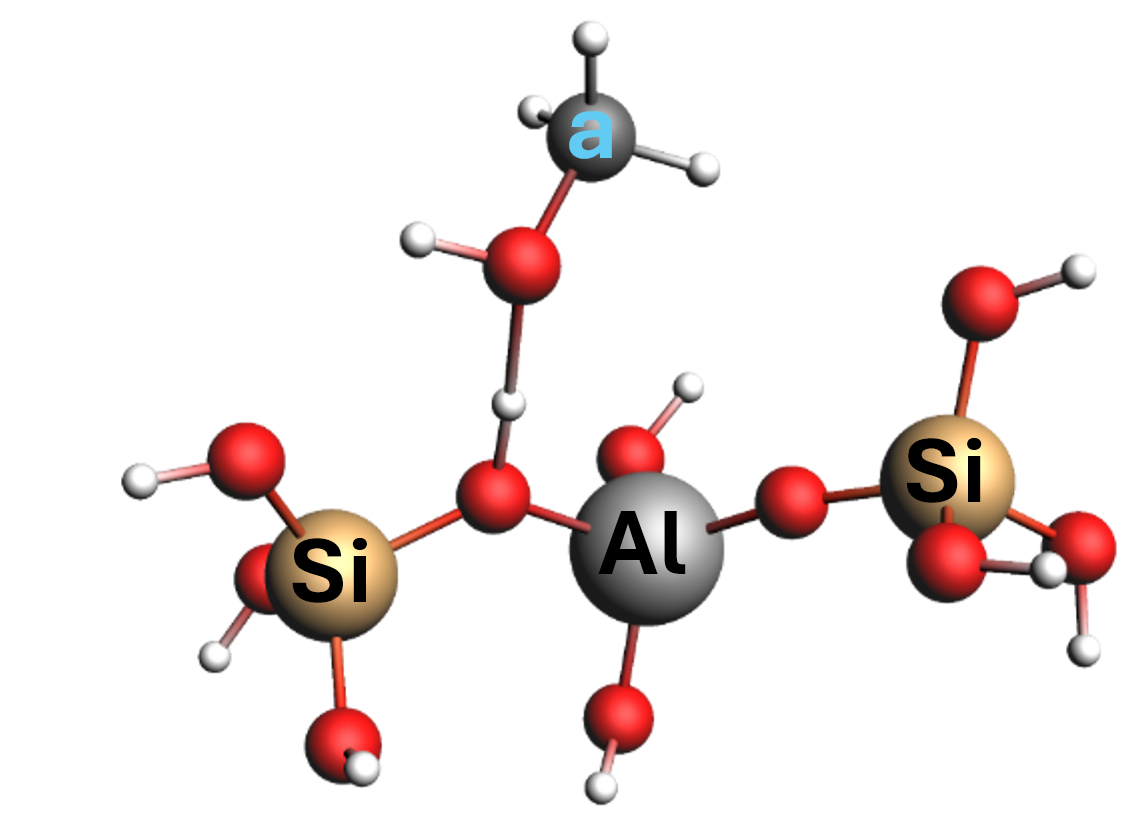  CH_3_OH bound at BAS in Al-BEA | -- | a = 49.5  Bond lengths:   - BA-O-H (O-H) = 1.049 Å - BA-H to O (-OH, CH_3_OH) = 1.513 Å | a = 49.6  Bond lengths:   - BA-O-H (O-H) = 1.047 Å - BA-H to O (-OH, CH_3_OH) = 1.519 Å |

**Table S10**. Comparisons between DFT-predicted peak shifts and bond lengths without the COSMO model (i.e., gas-phase) and with the COSMO model in a solvent continuum of acetonitrile or methanol.

Comparison of the predicted and calculated peak shifts for the liquid phase (Table S8) and surface-bound species (Table S9) to the experimentally observed peak shifts yields some interesting observations and points of discussion. First, the DFT predictions appear to frequently predict a shift further downfield for the (a) carbon attached to the chlorine within C_3_H_5_ClO, intermediate carbocations, and the ring-opened products. The experimentally obtained spectra for C_3_H_5_ClO and terminal ether standards confirm this over-prediction, as the DFT predicts shifts for (a) that are ~4 and ~8 ppm greater than those demonstrated by experiment for the respective molecules. While we cannot confirm the same for other products or intermediates (no standards available), we expect a similar over-prediction downfield from the DFT-calculated shifts.

A significant overlap of peaks occurs in the 45-55 ppm range in spectra collected during C_3_H_5_ClO ring-opening with CH_3_OH, as shown in Figure 4 and sections below (*vide infra*). This region contains peaks originating from liquid-phase and adsorbed CH_3_OH-derived species, the (a) carbon on liquid-phase C_3_H_5_ClO, intermediate carbocations, and the ring-opened products, and the (c) carbon on liquid-phase C_3_H_5_ClO. Therefore, we utilized the peak areas of carbon atoms that are not convoluted to track peak growth and measure the concentrations of the products and reactants in the NMR rotor. We utilized the secondary epoxide carbon (b) for C_3_H_5_ClO and secondary carbon (b) in both the terminal ether and terminal alcohol. We acknowledge that the formation of the TE product convolutes the primary carbocation features in the range of 69-73 ppm, which likely increases the error of peak area deconvolution for these species. We assume that all carbons show equal peak areas, supported by comparisons of the peak areas of the epoxide carbons in the absence of the reaction.

Comparison of the DFT-computed peak shifts for the (b) and (c) carbons in the secondary carbocation bound to Al-BEA (b = 89.0 ppm, c = 63.0 ppm), terminal alcohol bound to Al-BEA (b = 91.6 ppm, c = 61.8 ppm), and liquid-phase terminal alcohol (b = 85.7 ppm, c = 68.5 ppm) provide insight into intermediate and product formation in Figure 4a. The broad nature of the evolving peak between 80-90 ppm suggests that all three of the (b) carbons from these species contribute and convolute peak assignments. However, the peaks at ~63 and ~67 ppm match well with the (c) carbon predictions for the secondary carbocation and liquid-phase terminal alcohol, which appear much more intensely than a very minor peak at ~62 ppm that matches the bound terminal alcohol prediction. Considering these intensities and the growth patterns of the peaks, where the ~63 ppm peak first evolves and is followed by the ~67 ppm peak, the product formation pathway becomes clear. The secondary carbocations first form on the surface, then the terminal alcohol product desorbs quickly once formed (explaining the very low intensity of bound terminal alcohol features). Cross-referencing the (c) carbon predictions for Sn-BEA bound species with Figure 4b yields a similar observation of rapid desorption of the terminal alcohol following secondary carbocation formation and reaction with CH_3_OH. Figure S32 (*vide infra*) presents an analogous experiment to Figure 4, except Figure S32 shows faster and greater accumulation of intermediate and product peaks because the reaction was initiated by adding all species to the NMR rotor simultaneously. The evolution of the peaks at ~62, ~63, and ~67 ppm in Figure S32 illustrates the full cycle of terminal alcohol formation (further discussion provided below).

DFT predicts that coordination of the terminal alcohol product to a framework Sn atom in Sn-BEA is highly unfavorable (bond length = 6.202 Å) (Table S9). The terminal alcohol instead prefers to bind to an adjacent Si-OH group (bond length = 1.732 Å). In contrast, the terminal ether product binds more favorably to framework Sn (bond length = 2.481 Å). The difference between the binding configurations of the products may align with the significantly greater selectivity to the terminal ether during C_3_H_3_ClO ring-opening over Sn-BEA (Figure 7). While the peak areas in Figure 2 predict a primary to secondary carbocation ratio of 2.4 over Sn-BEA, the unfavorable binding of the terminal alcohol to Sn sites may contribute to the preference of Sn-BEA to form the terminal ether through either S_N_1 or S_N_2 reaction pathways.

The DFT-computed shifts for the (b) and (c) carbons unfortunately overlap more significantly for the primary carbocation bound to Al-BEA (b = 74.6 ppm, c = 71.1 ppm), terminal alcohol bound to Al-BEA (b = 77.2 ppm, c = 73.6 ppm), and liquid-phase terminal alcohol (b = 77.9 ppm, c = 75.9 ppm), with a similar issue for Sn-BEA as well. The narrow distribution of peak shifts aligns with the convoluted features observed from 69-76 ppm in Figure 4a (as discussed above), which precludes the precise observation of product formation and desorption possible for the terminal alcohol product. Nevertheless, the qualitative observation of evolving peak features in this region still demonstrates the formation of primary carbocations and the subsequent formation of terminal ether species.

Lastly, the predicted DFT binding structures for the secondary carbocation and terminal alcohol species suggest that these species will bind to the surface in a flatter configuration than other bound species, including CH_3_OH, C_3_H_5_ClO, the primary carbocation, and terminal ether. The positively charged secondary carbon should coordinate with the proton in the secondary carbocation species, while the terminal alcohol product should bind through the -OCH_3_ group that attaches to the same secondary carbon. This observation likely explains why the peaks originating from the secondary carbon in the terminal alcohol and secondary carbocation appear much more broadly than any other peaks in the NMR spectra, which makes these peaks difficult to observe and deconvolute. The flat binding configuration of these species on the zeolite surface likely leads to less freedom of motion for the secondary carbon compared to the other carbon atoms in the same molecules, which may cause the secondary carbon peaks to appear more broadly and difficult to observe.

**S12.2. ^13^C NMR Spectra for Liquid-Phase C_3_H_5_ClO and CH_3_OH**


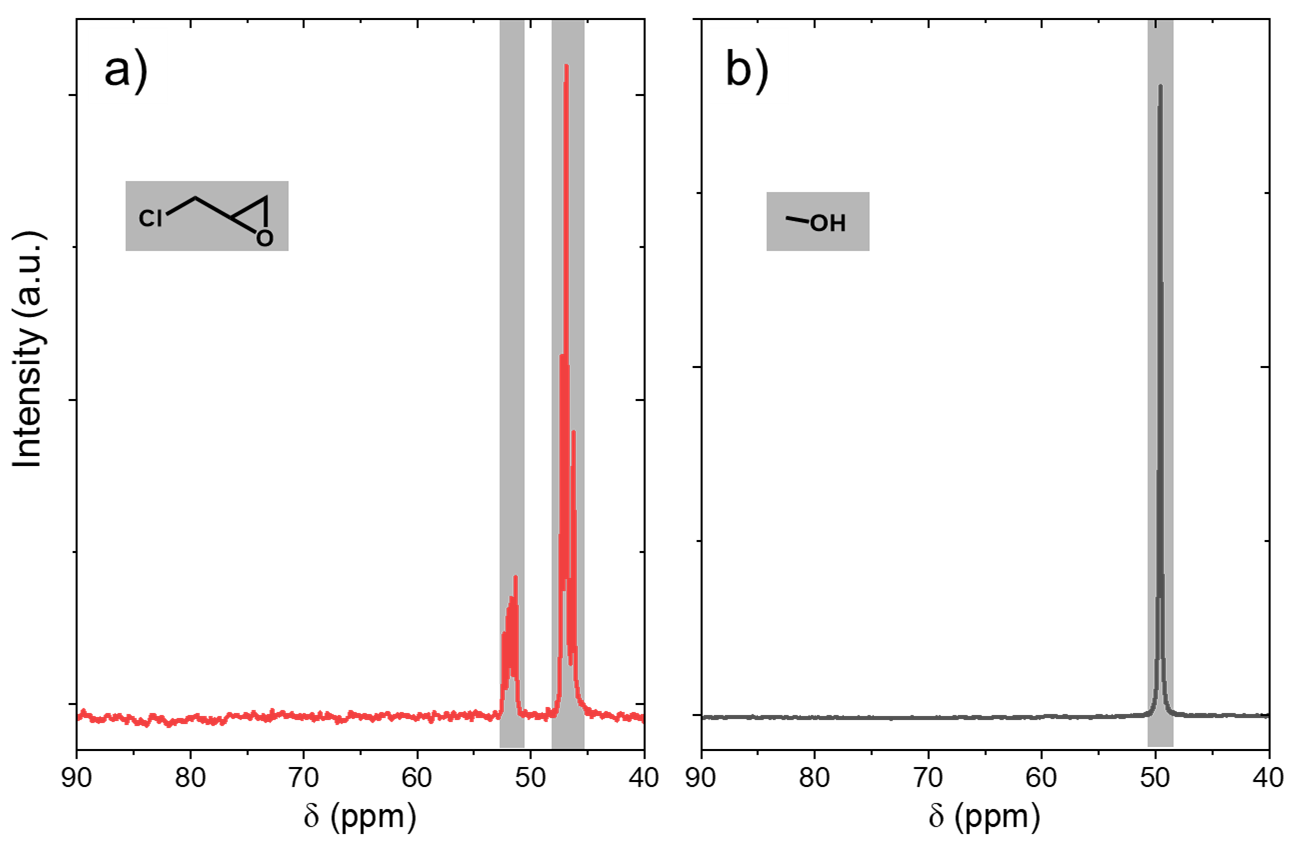
**Figure S22**. ^13^C NMR single-pulse spectra of a) C_3_H_5_ClO and b) CH_3_OH without catalyst present (0.2 M ^13^C_3_H_5_ClO or ^13^CH_3_OH, CH_3_CN, 298 K). Spectra are an average of 268 scans (C_3_H_5_ClO) and 68 scans (CH_3_OH).

Figure S22 displays single-pulse spectra for C_3_H_5_ClO and CH_3_OH diluted in CH_3_CN and without catalyst present. These spectra show the characteristic ^13^C peak shifts for the carbons within C_3_H_5_ClO and CH_3_OH, highlighted in red and black, respectively. As discussed in Section S12.1, the DFT-calculated peak shifts closely predict all peak locations except for the carbon directly bound to -Cl in C_3_H_5_ClO.

**S12.3. ^13^C NMR Spectra and Analysis for C_3_H_5_ClO Adsorption and Carbocation Formation**


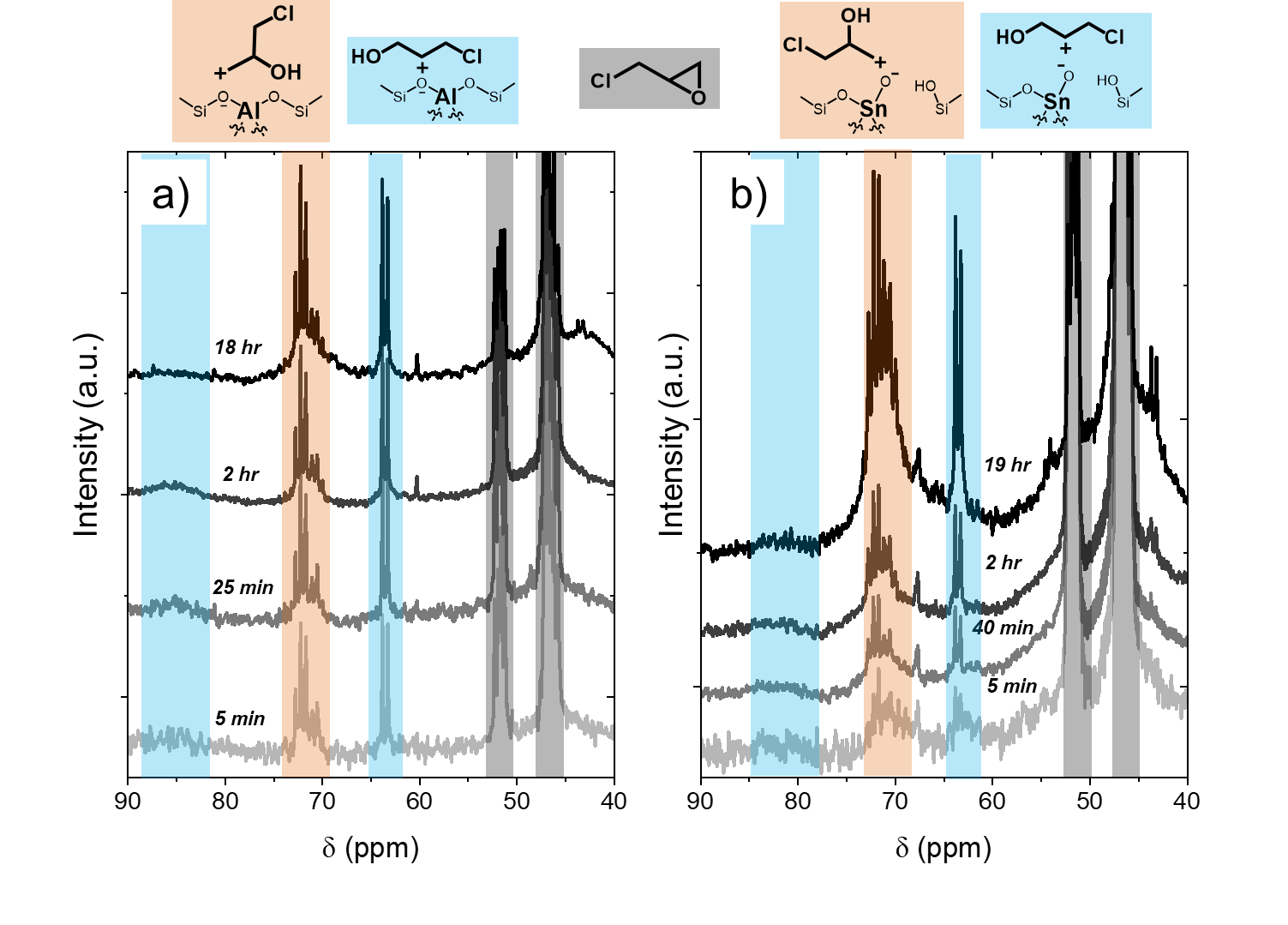


**Figure S23**. ^13^C NMR single-pulse spectra collected as a function of time during adsorption of C_3_H_5_ClO to a) Al-BEA and b) Sn-BEA (0.2 M ^13^C_3_H_5_ClO, CH_3_CN, 298 K). Spectra are an average of scans, ranging from 24 scans (5 min time points) to 256 scans (last time points).

Figure S23 shows the single-pulse spectra for C_3_H_5_ClO adsorption as a function of time. The peak assignments match well with the computationally predicted and experimentally calculated peak shifts shown in Section S12.1. The peak areas of the ring-opened carbocation species increase relative to C_3_H_5_ClO over time, supporting that more C_3_H_5_ClO opens to form carbocations over time. The carbocation peaks accumulate less rapidly over Sn-BEA than Sn-BEA. The open sites (Sn-OH) within Sn-BEA likely have a weaker Brønsted acidity than the Brønsted acid sites within Al-BEA, which likely leads to a slower activation of C_3_H_5_ClO and a lower quantity of ring-opened carbocations over Sn-BEA. Furthermore, the peak areas of the carbocation features do not decrease over a long period (> 18 hr). The stability of these features supports that the C_3_H_5_ClO irreversibly ring-opens to form stable carbocations on the Al-BEA and Sn-BEA surface.

Notably, the ratio of primary to secondary ring-opened carbocations over Sn-BEA during the ring-opening reaction within the NMR rotor shown in Figures 5 and 6 (1.2 – 2.5) of the main text generally falls below the ratio observed in the absence of the reaction (2.4, Figure 2 of main text). In contrast, the ratio during the reaction over Al-BEA (1.3 – 2.3) typically exceeds the ratio without the reaction occurring (1.4, Figure 2). Nevertheless, the different trends in the carbocation ratios between Sn-BEA and Al-BEA in the presence and absence of the ring-opening reaction likely signal that the reactivity of these carbocations differs between the two catalysts. The primary carbocation may show a greater reactivity over Sn-BEA, which reduces the quantity of these species and lowers the carbocation ratio on the surface, while the primary carbocations may have lesser reactivity over Al-BEA and lead to a greater carbocation ratio on the surface. Despite the difference in carbocation ratios between Figure 2 and Figures 5 and 6, the greater prevalence of the primary relative to secondary ring-opened carbocation holds in all cases. The preference towards forming the primary carbocation, originating from differences in $k_{5,{}^{1}C}$ and $k_{5,{}^{2}C}$, likely primarily governs $\beta$ differences at low [CH_3_OH]:[C_3_H_5_ClO] ratios in Figure 7.


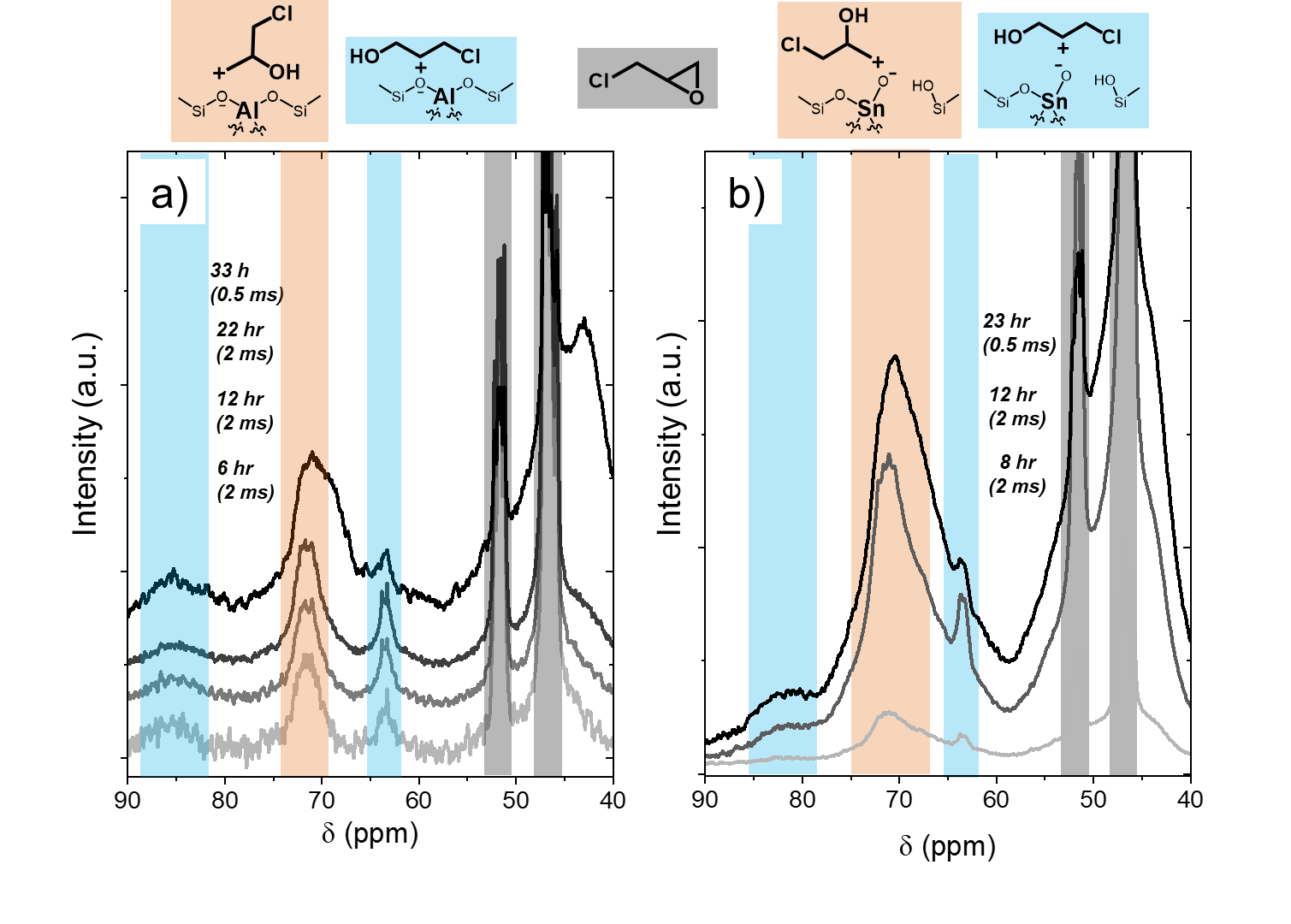


**Figure S24**. ^13^C NMR cross-polarization spectra collected as a function of time during adsorption of C_3_H_5_ClO to a) Al-BEA and b) Sn-BEA (0.2 M ^13^C_3_H_5_ClO, CH_3_CN, 298 K). Contact times are shown in parentheses. Spectra are an average of scans, ranging from 756 to 10,000 scans.

Figure S24 reveals the cross-polarization spectra for C_3_H_5_ClO adsorption over time. Features corresponding to the ring-opened carbocations and intact C_3_H_5_ClO both appear in the spectra, aligning with tabulated values from Section S12.1. The broad nature of the carbocation features supports that these species are bound to the surface. The sharp nature of the C_3_H_5_ClO in Al-BEA suggests that these features originate from liquid-phase C_3_H_5_ClO that resides near the surface but is not bound to the surface. In contrast, the broader nature of the C_3_H_5_ClO features over Sn-BEA supports that a fraction of surface C_3_H_5_ClO exists as an intact molecule rather than ring-opened carbocations.

Of note, the intensity of the ring-opened carbocation peaks increases relative to intact C_3_H_5_ClO when the contact time is decreased from 2 ms to 0.5 ms. This supports that the ring-opened carbocations bind more strongly to the surface than C_3_H_5_ClO.

**S12.4. ^13^C NMR Spectra and Analysis for CH_3_OH Adsorption**

Figure S25 displays the single-pulse spectra for CH_3_OH adsorption as a function of time. The peak for physisorbed CH_3_OH at Si-O-Si features (green) appears from very early times in both catalysts. in which the ring-opening reaction was initiated by adding CH_3_OH (0.2 M CH_3_OH). The chemisorbed (yellow) and protonated (purple) CH_3_OH species appear slowly over time in Al-BEA, demonstrating the formation of activated CH_3_OH intermediates at the Brønsted acid sites. Interestingly, chemisorbed CH_3_OH appears from much lower times within Sn-BEA, suggesting that Sn-BEA may activate more strongly and more rapidly over Sn-BEA than Al-BEA.

**
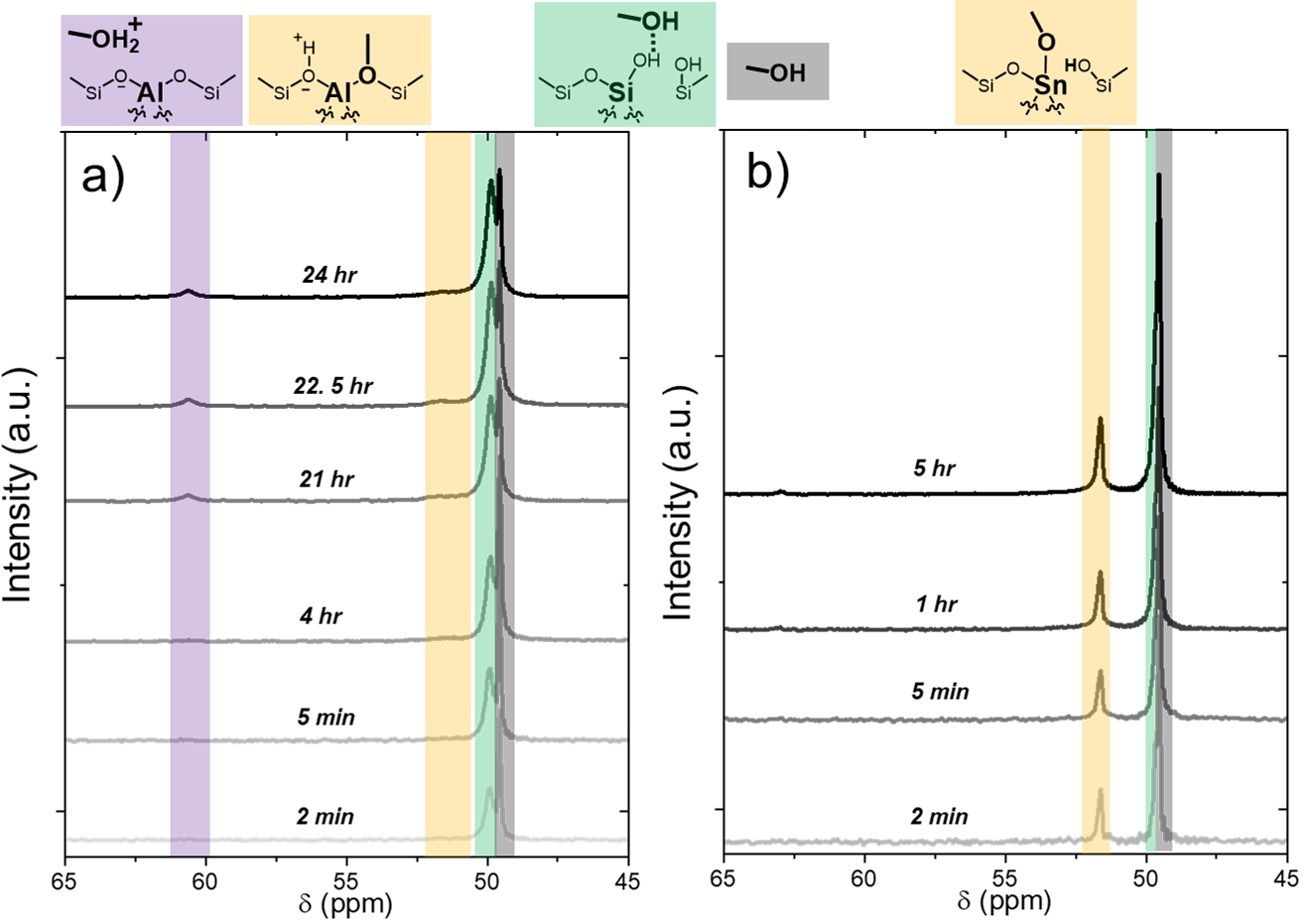
**

**Figure S25**. ^13^C NMR single-pulse spectra of ^13^CH_3_OH on a) Al-BEA and b) Sn-BEA (0.2 M ^13^CH_3_OH, CH_3_CN, 298 K) collected as a function of time after initial contact between the liquid solution and the zeolite. Spectra are an average of scans, ranging from 16 scans (2 min time points) to 2000 scans (last time points).

Figure S26 displays the cross-polarization spectra for CH_3_OH adsorption as a function of time. These spectra show the key adsorption peaks also observed in the single-pulse spectra in Figure S25: physisorbed CH_3_OH at Si-O-Si features (green), chemisorbed CH_3_OH (yellow), and protonated CH_3_OH (purple) species. Figure S26 provides further support that CH_3_OH adsorbs to the *BEA framework and activates at both Brønsted and Lewis acid sites.  Figure S27 reproduces the spectra from Figure 2 and 3 of the manuscript (Figures S27a-S27d) but with wider perspective along with a magnified view of the feature at ~ 1 ppm that represents the methyl carbon in CH_3_CN (Figures S27e-S7h). The peak at ~118 ppm appears for liquid-phase CH_3_CN but not on the cross-polarization spectra. This peak originates from the carbon atom bound to nitrogen in CH_3_CN. CH_3_CN would likely bind to the zeolite acid sites via the nitrogen atom (acid-base coordination), therefore, the absence of this feature in the cross-polarization spectra suggests that CH_3_CN does not bind at acid sites at measurable coverages. The peak at ~1 ppm shows a similar width between the cross-polarization spectra and the single-pulse spectra in the absence of the catalysts. This starkly contrasts with the C_3_H_5_ClO and CH_3_OH features, which broaden significantly within the cross-polarization spectra. The peak broadening results from adsorption to the catalyst surface. While the presence of the feature at ~1 ppm in the cross-polarization spectra supports that methyl carbons within CH_3_CN molecules reside near the surfaces of both Al- and Sn-BEA, the absence of broadening of this CH_3_CN peak demonstrates that the CH_3_CN molecules do not coordinate to the catalyst surface and therefore do not compete effectively against C_3_H_5_ClO and CH_3_OH for active sites.

**
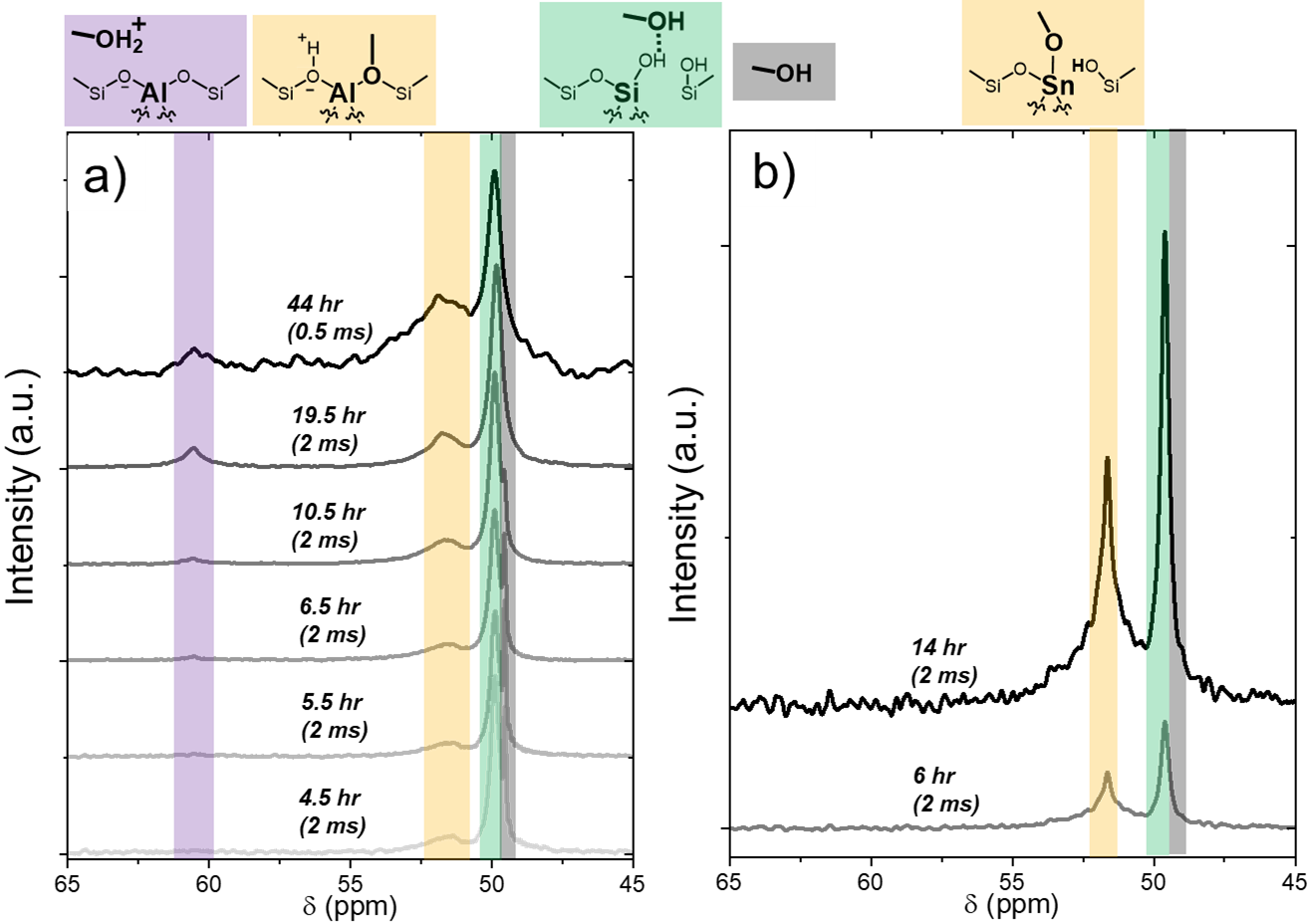
**

**Figure S26**. ^13^C NMR cross-polarization spectra of ^13^CH_3_OH on a) Al-BEA and b) Sn-BEA (0.2 M ^13^CH_3_OH, CH_3_CN, 298 K) collected as a function of time after initial contact between the liquid solution and the zeolite. Contact times are shown in parentheses. Spectra are an average of scans, ranging from 734 to 8,000 scans.


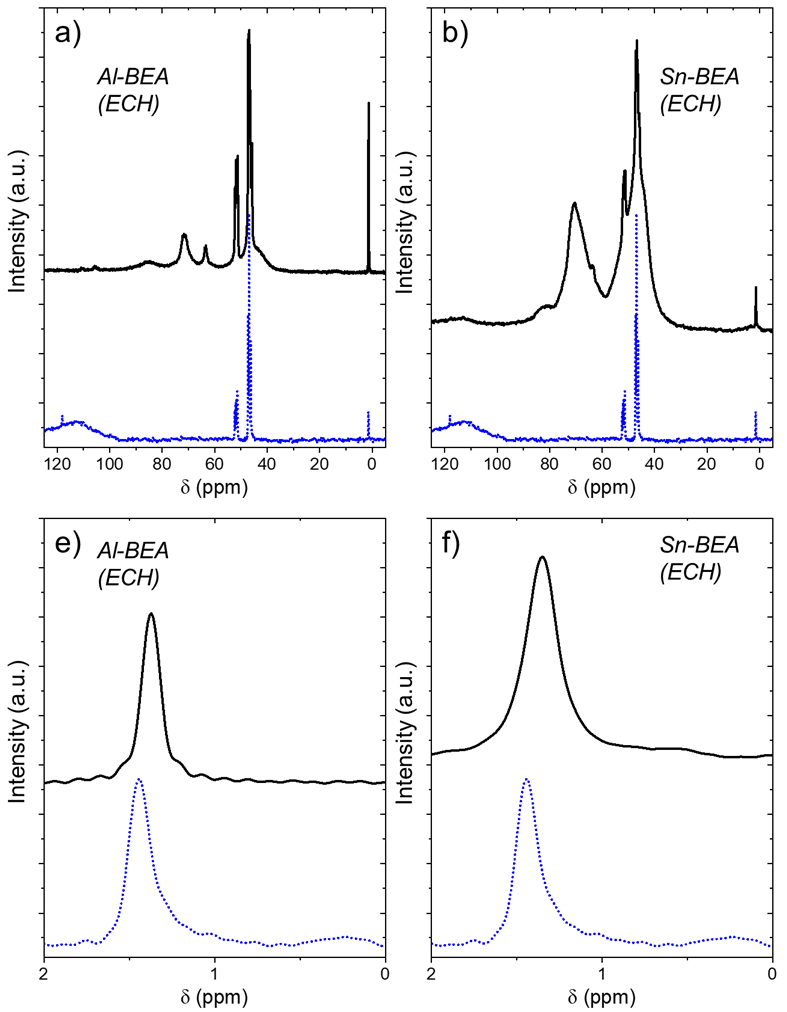

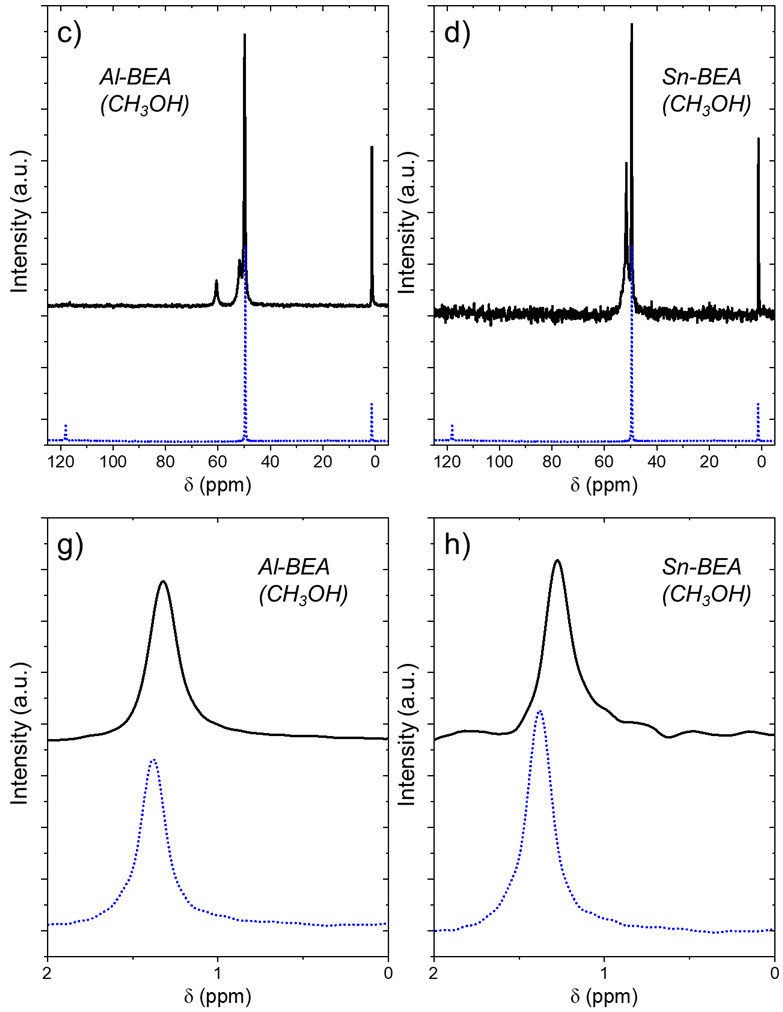


**Figure S27**. ^13^C NMR cross-polarization spectra (black lines) of ^13^C_3_H_5_ClO or ^13^CH_3_OH on Al-BEA or Sn-BEA (0.2 M ^13^C_3_H_5_ClO or ^13^CH_3_OH, CH_3_CN, 298 K, 12-20 hr after initial contact between the liquid solution and the zeolite). Blue dotted lines show the single-pulse spectra for ^13^C_3_H_5_ClO or ^13^CH_3_OH in the absence of catalyst. Parts a-d show the full spectra, while parts e-h zoom into the CH_3_CN feature at ~1.5 ppm.

Furthermore, Table S9 (entries 26 and 27) shows that the DFT-NMR predicted peak shifts for CH_3_CN adsorbed to acid sites in Sn-BEA and Al-BEA fall 10-11 ppm upfield for the methyl carbon (-9.0, -9.4 ppm) and 3-5 ppm upfield for the carbon bound to the nitrogen atom (113.8, 115.6 ppm) relative to the observed peaks. The differences in the predicted and observed peak shifts further supports that CH_3_CN does not competitively adsorb to the acid sites in Al- or Sn-BEA.

**S12.5. Supplemental ^13^C NMR Spectra from C_3_H_5_ClO Ring-Opening with CH_3_OH**


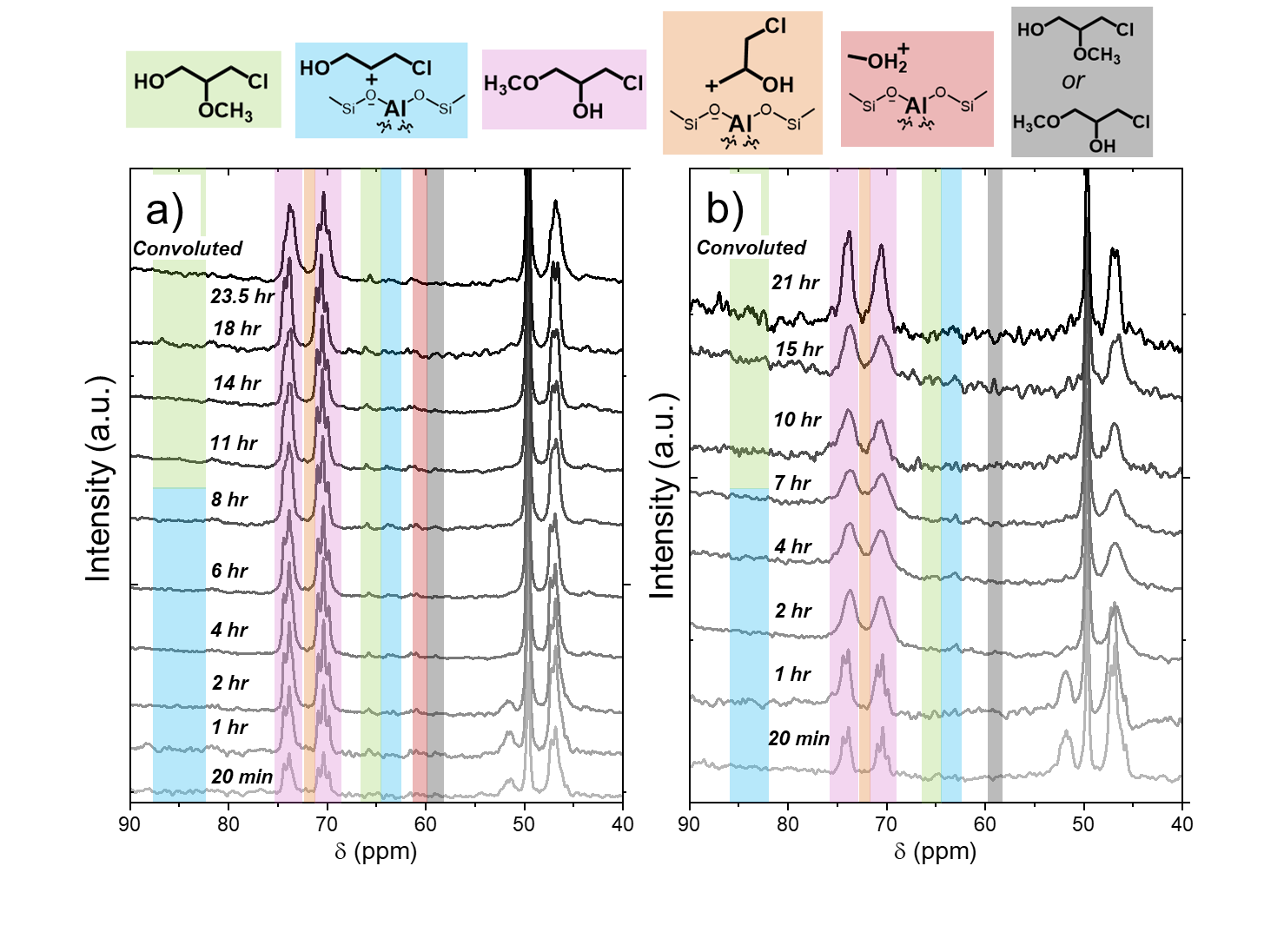


**Figure S28**. ^13^C NMR single-pulse spectra during ring-opening of C_3_H_5_ClO with CH_3_OH over a) Al-BEA and b) Sn-BEA, where reaction was initiated by adding all components to the rotor at once (0.2 M ^13^C_3_H_5_ClO, 6 M CH_3_OH, CH_3_CN, 298 K). Spectra are an average of scans, ranging from 64 scans (20 min time points) to 2,000 scans (last time points). *Note: these spectra were collected with unlabeled CH_3_OH, so CH_3_OH-derived features appear much less intensely.*

Figure S28 presents the single-pulse NMR spectra collected during the ring-opening reaction of C_3_H_5_ClO at 6 M CH_3_OH. The concentration-time profiles derived from these spectra are presented in Figure 6 of the main text. Compared to 0.2 M CH3OH, the carbocation species derived from are more difficult to observe. These features at low intensities over time in Al-BEA (Figure S28a), while the carbocation species do not appear in noticeable quantities over Sn-BEA (Figure S28b). The negligible accumulation of these ring-opened carbocations over both Al-BEA and Sn-BEA in Figure S28 gives evidence that the materials may catalyze ring-opening through similar reaction pathways in CH_3_OH-rich conditions.


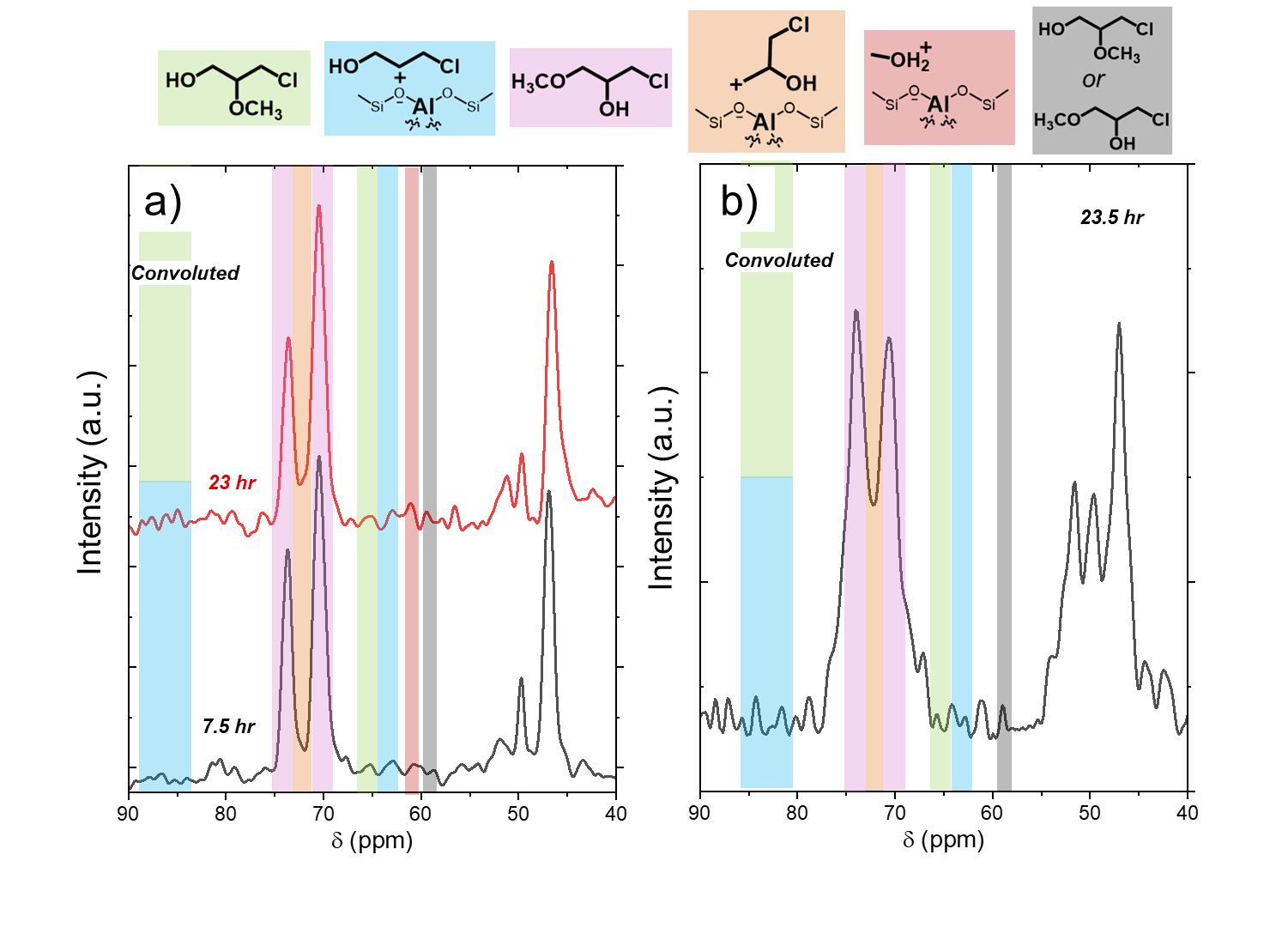
**Figure S29**. ^13^C NMR cross-polarization spectra during ring-opening of C_3_H_5_ClO with CH_3_OH over a) Al-BEA and b) Sn-BEA, where the reaction was initiated by adding all components to the rotor at once (0.2 M ^13^C_3_H_5_ClO, 6 M CH_3_OH, CH_3_CN, 298 K). Contact time was 1 ms for all spectra shown here. Spectra are an average of scans, ranging from 300 to 1500 scans. *Note: these spectra were collected with unlabeled CH_3_OH, so CH_3_OH-derived features appear much less intensely.*

Figure S29 presents the cross-polarization spectra for the same experiment discussed in Figure S28 (6 M CH_3_OH). The ring-opened carbocation species are not visible on the surface from these spectra, further supporting that C_3_H_5_ClO ring-opening proceeds through similar S_N_2 reaction pathways in CH_3_OH-rich conditions, which do not require the formation of the ring-opened carbocations as intermediates.

Figure S30 displays the cross-polarization spectra for the experiments shown with the single-pulse spectra in Figure 4 of the main text, where the reaction was initiated by adding C_3_H_5_ClO (0.2 M CH_3_OH). The spectra show a greater quantity of the ring-opened carbocations on the surface of Al-BEA than Sn-BEA, aligning with the interpretation from the single-pulse spectra. The difference in carbocation concentration supports that Al-BEA and Sn-BEA may catalyze ring-opening through different mechanisms in CH_3_CN-rich conditions.


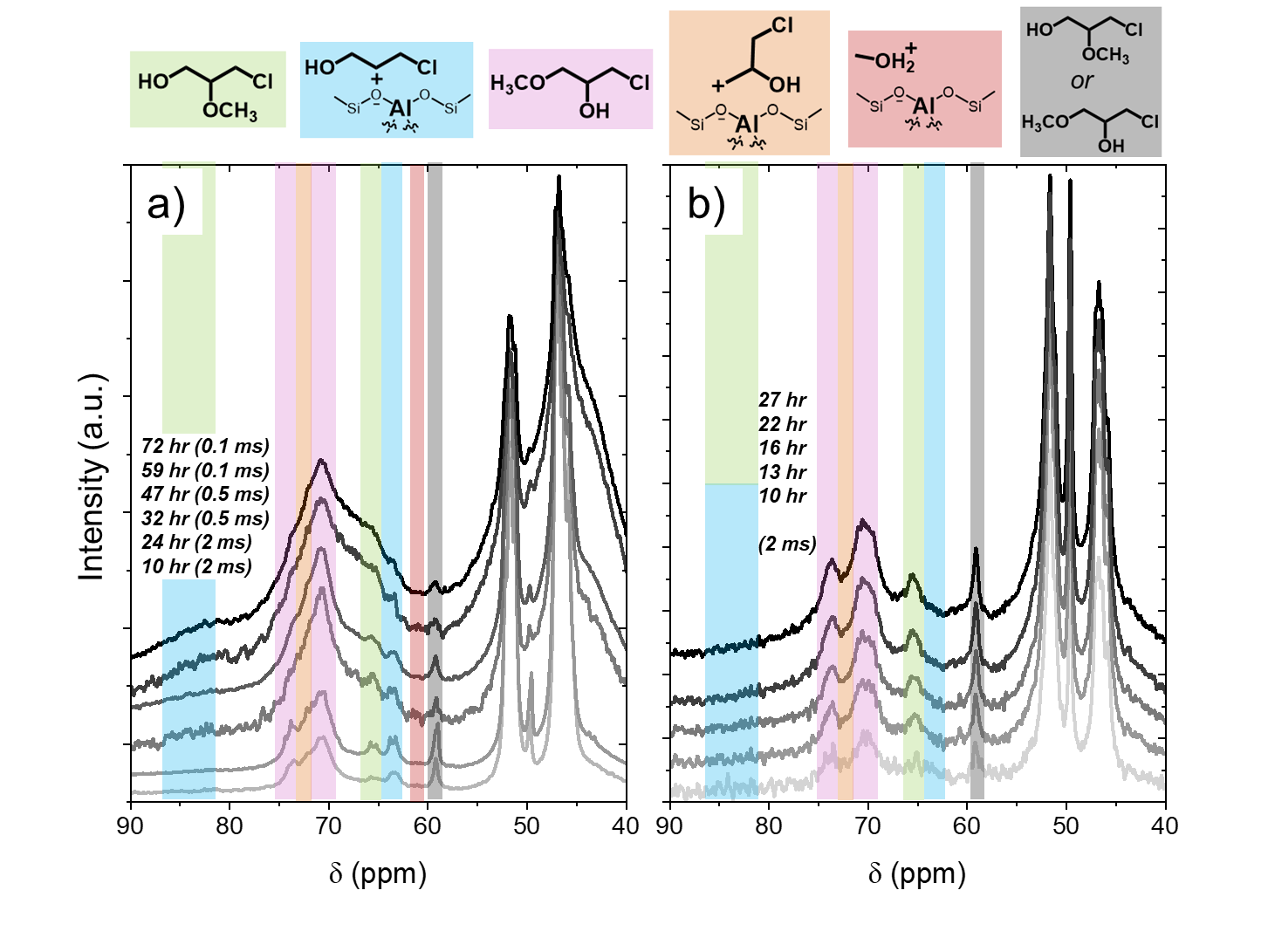


**Figure S30**. ^13^C NMR cross-polarization spectra during ring-opening of C_3_H_5_ClO with CH_3_OH over a) Al-BEA and b) Sn-BEA, where reaction was initiated by adding ^13^C_3_H_5_ClO to the NMR rotor after pre-mixing all other components (0.2 M ^13^C_3_H_5_ClO, 0.2 M ^13^CH_3_OH, CH_3_CN, 298 K). Contact times are shown in parentheses; the contact time was 2 ms for all spectra in Sn-BEA. Spectra are an average of scans, ranging from 468 to 13,200 scans.

Interestingly, the difference in contact times in Figure S30a leads to differences in the ratio of adsorbed species on Al-BEA. The peak area ratio of CH_3_OH-derived adsorbates (intact CH_3_OH, protonated CH_3_OH) to C_3_H_5_ClO-derived species (intact C_3_H_5_ClO, ring-opened carbocations, products) decreases as the contact time decreases. A lower contact time leads to greater intensities for more strongly-bound species, suggesting that C_3_H_5_ClO-derived adsorbates coordinate more strongly to the Al-BEA surface than CH_3_OH-derived species. This interpretation aligns with the much more exothermic adsorption enthalpy for C_3_H_5_ClO than CH_3_OH shown in Table S7 (*vide supra*).

Figure S31 presents single-pulse spectra where the ring-opening reaction was initiated by adding CH_3_OH (0.2 M CH_3_OH). Interestingly, initiating the reaction with CH_3_OH slows the reaction down significantly compared to initiating with C_3_H_5_ClO. The ring-opened carbocations form before the reaction was initiated with CH_3_OH, but only a small fraction converts to products over ~24 hr.


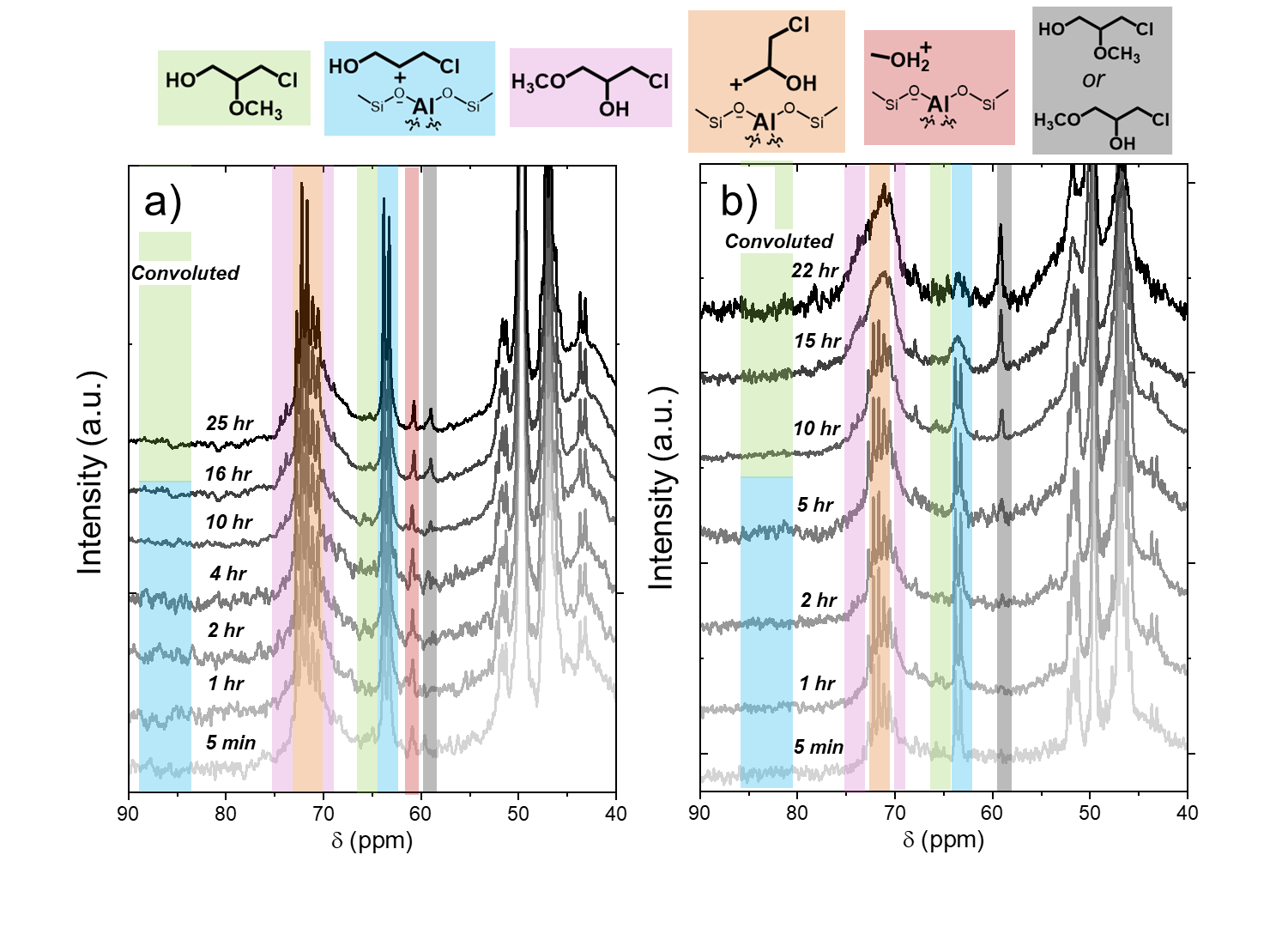


**Figure S31**. ^13^C NMR single-pulse spectra during ring-opening of C_3_H_5_ClO with CH_3_OH over a) Al-BEA and b) Sn-BEA, where reaction was initiated by adding ^13^CH_3_OH to the NMR rotor after pre-mixing all other components (0.2 M ^13^C_3_H_5_ClO, 0.2 M ^13^CH_3_OH, CH_3_CN, 298 K). Spectra are an average of scans, ranging from 32 scans (5 min time points) to 15,768 scans (last time points).

These spectra may suggest that CH_3_OH still needs to activate in some capacity to facilitate the ring-opening reaction, even through an S_N_1 pathway with ring-opened carbocations. C_3_H_5_ClO coordinates much more strongly with the active sites than CH_3_OH, so the pre-treatment of the catalyst with C_3_H_5_ClO may prevent CH_3_OH from easily accessing the active sites.

While the exact reason for the suppression of rates when initiating with CH_3_OH currently evades understanding, Figure S31 still provides strong evidence that the ring-opened carbocations convert to the terminal ether and alcohol products through an S_N_1 pathway.


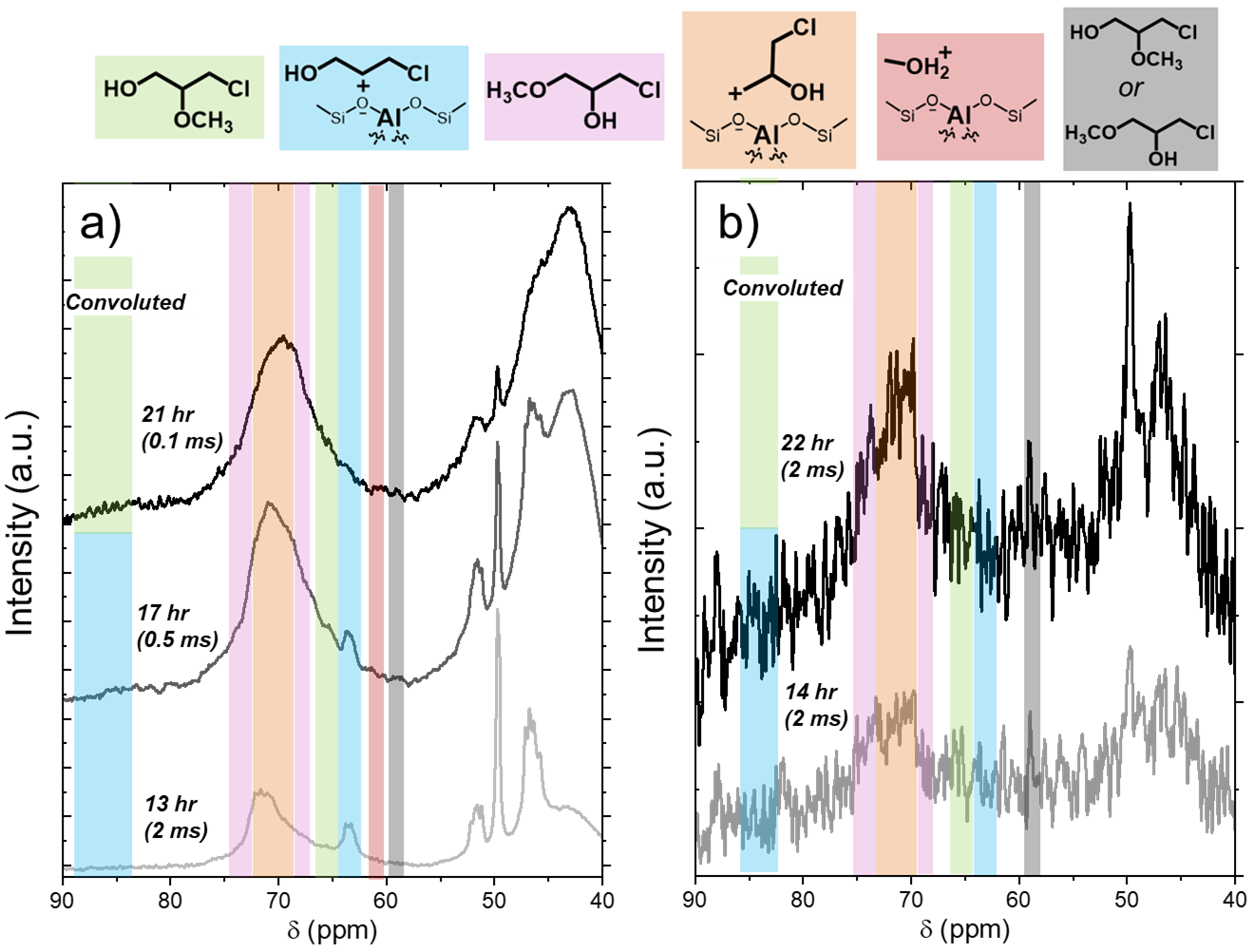


**Figure S32**. ^13^C NMR cross-polarization spectra during ring-opening of C_3_H_5_ClO with CH_3_OH over a) Al-BEA and b) Sn-BEA, where reaction was initiated by adding ^13^CH_3_OH to the NMR rotor after pre-mixing all other components (0.2 M ^13^C_3_H_5_ClO, 0.2 M ^13^CH_3_OH, CH_3_CN, 298 K). Contact times are shown in parentheses. Spectra are an average of scans, ranging from 754 to 4,096 scans.

Figure S32 presents the cross-polarization spectra from the same experiment in Figure S32, in which the ring-opening reaction was initiated by adding CH_3_OH (0.2 M CH_3_OH). As with Figure S30a, decreasing the contact time leads to a decrease in the relative intensity of CH_3_OH-derived species over Al-BEA in Figure S32a, suggesting that C_3_H_5_ClO-derived species coordinate more strongly to the surface.

Figure S33 presents single-pulse spectra where the ring-opening reaction was initiated by adding all the components to the rotor at the start of the reaction (0.2 M CH_3_OH). This experiment was carried out at an identical CH_3_OH concentration to the experiments where the reaction was initiated by adding C_3_H_5_ClO (Figure 4 of main text) or CH_3_OH (Figure S30).


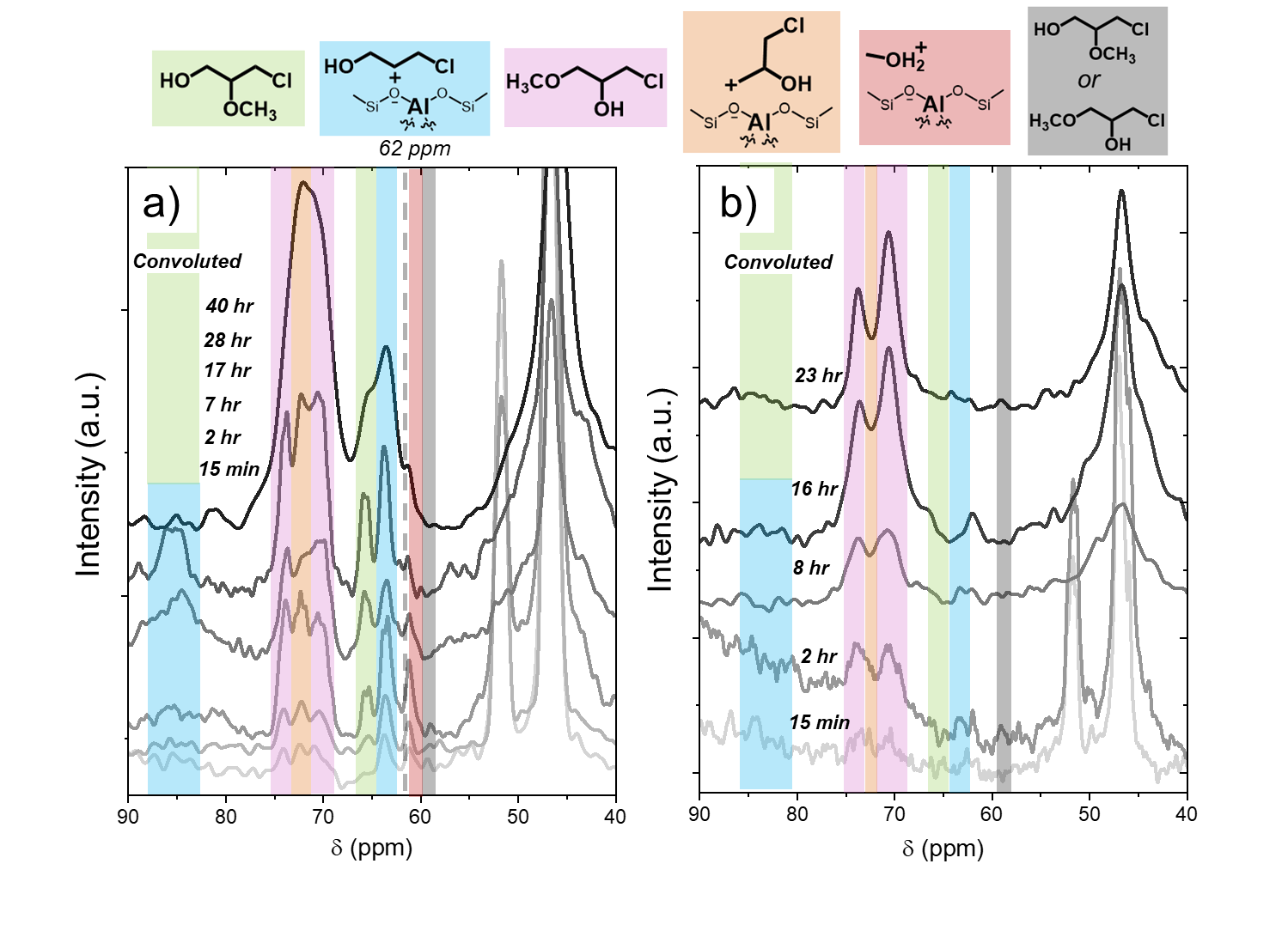


**Figure S33**. ^13^C NMR single-pulse spectra during ring-opening of C_3_H_5_ClO with CH_3_OH over a) Al-BEA and b) Sn-BEA, where reaction was initiated by adding all components to the rotor at once (0.2 M ^13^C_3_H_5_ClO, 0.2 M CH_3_OH, CH_3_CN, 298 K). Spectra are an average of scans, ranging from 32 scans (15 min time points) to 3024 scans (last time points). *Note: spectra were collected with unlabeled CH_3_OH, so CH_3_OH-derived features appear much less intensely.*

We chose to present the data where C_3_H_5_ClO initiates the reaction in Figure 4 in the main text because initiating with C_3_H_5_ClO slows down the reaction to enable easier tracking of the reactant, product, and intermediate peaks. In particular, the method of initiating with C_3_H_5_ClO demonstrates the transformation of the ring-opened carbocation intermediates to ring-opened products, as discussed above in Section S12.1. Interestingly, Figure S33 shows an intense peak at ~62 ppm over both Al- and Sn-BEA that is only observed with very weak intensity in Figure 4, which initially increases and then decreases in intensity over time. This peak in Figure S33 does not originate from a CH_3_OH-derived intermediate as observed at ~60.5 ppm in Figure 4 within the region highlighted red because Figure S33 was collected with unlabeled CH_3_OH, as even liquid-phase CH_3_OH appears hardly visible on Figure S33. Instead, the peak at ~62 ppm likely originates from the terminal alcohol bound to the zeolite surface, as predicted with the DFT peak shifts in Table S9. Therefore, the faster reaction rate in Figure S33 coincides with the easier observation of the full process of terminal alcohol formation (first discussed in Section S12.1). The secondary carbocation (~63 ppm) and bound terminal alcohol (~62 ppm) appear in the initial scans, with a greater intensity for the secondary carbocation that suggests the carbocation initially forms and then converts to the terminal alcohol. The liquid-phase terminal alcohol (~67 ppm) then appears and increases in intensity in the later scans, with a subsequent decrease of intensity for the bound terminal alcohol peak that shows desorption of the formed product over time. The secondary carbocation peak maintains the strongest intensity of the three peaks throughout the reaction, demonstrating consistent coverage of the active sites with this intermediate. Despite the differences in intensity of the product and reactant features, the ring-opening product peaks evolve in a very similar distribution for Figures 4 and S33. This suggests that the method of initiating the reaction does not affect the intrinsic behavior or regioselectivity of the reaction, but instead allows for control of the reaction rate and monitoring of specific intermediate and product peaks.

**
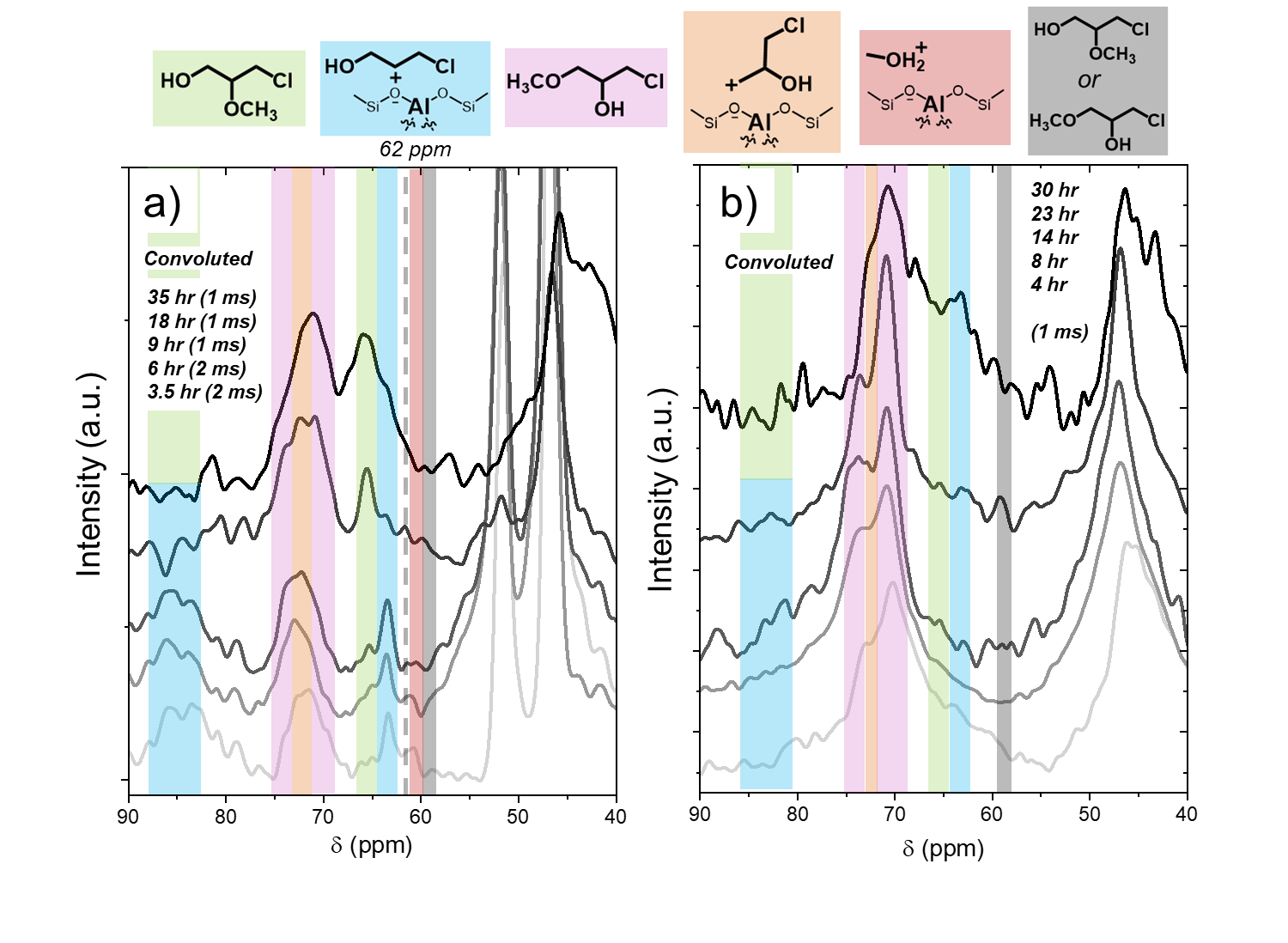
Figure S34**. ^13^C NMR cross-polarization spectra during ring-opening of C_3_H_5_ClO with CH_3_OH over a) Al-BEA and b) Sn-BEA, where reaction was initiated by adding all components to the rotor at once (0.2 M ^13^C_3_H_5_ClO, 0.2 M CH_3_OH, CH_3_CN, 298 K). Contact times are shown in parentheses; the contact time was 1 ms for all spectra in Sn-BEA. Spectra are an average of scans, ranging from 2564 to 10,094 scans. *Note: these spectra were collected with unlabeled CH_3_OH, so CH_3_OH-derived features appear much less intensely.*

Figure S34 shows the cross-polarization spectra that correspond to the single-pulse spectra in Figure S33, in which all components were added to the rotor to initiate the reaction (0.2 M CH_3_OH). These spectra demonstrate the growth of the carbocation intermediate and ring-opened product species on the surface, as most clearly demonstrated for terminal alcohol over Al-BEA in Figure S34a. The growth patterns of the secondary carbocation (~63 ppm), bound terminal alcohol (~62 ppm), and liquid-phase terminal alcohol (~67 ppm) align with those described in Figure S32 and show the sequence of carbocation formation, product formation, and product desorption. The appearance of the peak at ~67 ppm that we attribute to the liquid-phase terminal alcohol is unexpected in the cross-polarization spectra but resembles the observation of liquid-phase C_3_H_3_ClO features from Figure 2 of the main text. We attribute this to desorbed terminal alcohol product species that remain near the zeolite surface. Figure S34 shows very similar peak growth trends to those shown in Figure S30, where the reaction was initiated by adding C_3_H_5_ClO.

**S12.6. Kinetic Comparisons from Batch Reactions and *Operando* ^13^C NMR**

| **Al-BEA** | **Batch Kinetics** | | ***Operando* ^13^C NMR** | |
| --- | --- | --- | --- | --- |
| [CH_3_OH] | Product Formation Rate  (mol C_4_H_9_ClO_2_) • (mol M • s)^-1^) | $\beta= \frac{r_{TE}}{r_{TA}}$ | Product Formation Rate  (mol C_4_H_9_ClO_2_) • (mol M • s)^-1^) | $\beta= \frac{r_{TE}}{r_{TA}}$ |
| 0.2 M | 5.31 x 10^-4^ | 6.9 | 2.42 x 10^-5^ | 5.4 |
| 6 M | 5.63 x 10^-3^ | 15.3 | 4.58 x 10^-4^ | 11.6 |

**Table S11**. Comparisons of calculated total product formation rates and β values obtained from batch kinetics and *operando* ^13^C NMR measurements for C_3_H_5_ClO ring-opening over Al-BEA (0.2 M C_3_H_5_ClO, CH_3_CN, 298 K).

| **Sn-BEA** | **Batch Kinetics** | | ***Operando* ^13^C NMR** | |
| --- | --- | --- | --- | --- |
| [CH_3_OH] | Product Formation Rate  (mol C_4_H_9_ClO_2_) • (mol M • s)^-1^) | $\beta= \frac{r_{TE}}{r_{TA}}$ | Product Formation Rate  (mol C_4_H_9_ClO_2_) • (mol M • s)^-1^) | $\beta= \frac{r_{TE}}{r_{TA}}$ |
| 0.2 M | 1.42 x 10^-3^ | 12.1 | 5.96 x 10^-5^ | 9.0 |
| 6 M | 6.38 x 10^-3^ | 28.5 | 5.18 x 10^-4^ | 22.7 |

**Table S12**. Comparisons of calculated total product formation rates and β values obtained from batch kinetics and *operando* ^13^C NMR measurements for C_3_H_5_ClO ring-opening over Sn-BEA (0.2 M C_3_H_5_ClO, CH_3_CN, 298 K).

The concentration-time profiles for the NMR spectra from Figures 5 and 6 were used to measure product formation turnover rates and regioselectivities (β) for the ring-opening reaction in the NMR rotor. The concentrations were used to calculate turnover numbers for each time point in the NMR experiment, and a polynomial was fit to the plots of turnover number as a function of time. This procedure matches the method used to calculate turnover rates from the batch reactions (see Section S1.3).

Tables S11 and S12 show that the reaction rates are 10-25 times lower in the NMR than in the glass batch reactors used for the data in Figure 1. The significantly higher catalyst to volume ratio in the NMR experiments (~250 g L^-1^) compared to the batch reactions (~1 g L^-1^) may contribute to the lower rates per active site observed in the NMR through internal mass transfer limitations. The much greater concentration of active sites may lead to the under-utilization of active sites near catalyst particle interiors.

The rate differences may also partially originate from differences in the agitation mechanism and rate between the systems; a stir bar directly stirs the mixture in the batch reactors at 700 rpm, while the NMR rotor indirectly agitates the mixture by spinning at ~4400 Hz (~250,000 rpm). The NMR rotor does not stir the mixture, instead forming a relatively static hollow cylinder of catalyst and liquid due to centrifugal effects caused by the rapid rotation rate. The lack of stirring may contribute to external diffusion limitations of the reactants to the catalyst particles.

The higher catalyst to liquid ratio leads to greater concentrations of products in the NMR experiments than encountered in the BSTR experiments. Sn-BEA and Al-BEA reach respective C_3_H_5_ClO ring-opening conversions of 48% and 58% at 0.2 M CH_3_OH and 90% and 87% at 6 M CH_3_OH over 24 hours in the ^13^C NMR measurements (Figures 5 and 6, main text). In contrast, no batch reactor measurement achieves conversions greater than 10%. The methoxy peak originating from the TE and TA products appears at ~59 ppm and remains on the cross-polarization spectra throughout the reaction (Figures S30 and S31). This demonstrates that the products compete for active sites at the catalyst to liquid ratios utilized in the NMR measurements and may lead to product inhibition of rates. The combinations of external and internal transport limitations described above likely exacerbate the inhibitory effect.

Despite the rate differences, the NMR experiments and batch reactions show very similar β values at each [CH_3_OH] for both catalysts. Interestingly, the NMR experiments do consistently show slightly lower β values than the batch reactions. We believe this may originate from the NMR experiments being run to higher conversions, which may affect the reaction environment or concentration of surface-bound species in a way that promotes the terminal alcohol product over the terminal ether.

While the differences in spin rate, catalyst-to-volume ratio, and reaction conversion prevent exact comparisons between the NMR experiments and batch reactions, the qualitative regioselectivity agreement between these experiments supports that the NMR experiments capture the intrinsic S_N_1 and S_N_2 mechanisms and the competition between these mechanisms during C_3_H_5_ClO ring-opening.**S12.7. Comparison of Regioselectivity Trends for C_3_H_5_ClO and C_4_H_8_O Ring-Opening**

The combination of kinetic and spectroscopic data presented in the main text provides evidence that changes in the reaction mechanism drive significant differences in reaction regioselectivity for C_3_H_5_ClO ring-opening. Interestingly, β values over Al-BEA and Sn-BEA materials depend much less strongly on [CH_3_OH] for the ring-opening of 1,2-epoxybutane (C_4_H_8_O, 0.7 – 1.6)^19^ than for C_3_H_5_ClO (1 – 40) over *BEA zeolites. Our previous work on 1,2-epoxybutane argued that decreases in β with increasing [CH_3_OH] originated from CH_3_OH molecules stabilizing the transition state for TA formation through hydrogen bonding.^19^ The differences between C_4_H_8_O and C_3_H_5_ClO suggest that mechanistic differences play a much less significant role in regioselectivity changes for C_4_H_8_O ring-opening. The S_N_1 reaction pathway may dominate during C_4_H_8_O ring-opening, leading to only minor changes in β with [CH_3_OH] primarily driven by hydrogen-bonding effects through changes in ${\Delta G}^{\ddagger,\varepsilon}$. The apparent dominance of the S_N_2 pathway and more significant differences in β for C_3_H_5_ClO than C_4_H_8_O may originate from several possibilities (Scheme S4). First, the S_N_1 reaction pathway likely proceeds less favorably for C_3_H_5_ClO than C_4_H_8_O. The electron-withdrawing terminal Cl atom in C_3_H_5_ClO destabilizes positive charge, while the electron-donating terminal methyl (-CH_3_) group in C_4_H_8_O stabilizes positive charge. Therefore, the ring-opened carbocations likely form more easily from C_4_H_8_O, making the S_N_1 reaction more favorable for C_4_H_8_O through larger values of $k_{5,{}^{1}C}$ and $k_{5,{}^{2}C}$. Second, electron-withdrawing groups like Cl increase the rates of S_N_2 reactions by withdrawing electron density from the site of nucleophilic attack,^82-83^ which would increase values of $k_{4,{}^{1}C}$ and $k_{4,{}^{2}C}$ for C_3_H_5_ClO compared to C_4_H_8_O ring-opening. Furthermore, the presence of the Cl atom may promote TE formation through the S_N_2 reaction more significantly than the expected effect from steric hindrance. Cl may stabilize CH_3_OH through halogen bonding, drawing CH_3_OH from the proximal secondary epoxide carbon atom and further hindering the formation of TA. The different regioselectivity trends between C_3_H_5_ClO and C_4_H_8_O suggest that functional groups on epoxides lead to mechanistic differences that significantly influence product distribution from ring-opening. We anticipate that epoxides with electron-donating groups, like C_4_H_8_O and styrene oxide, will show weak regioselectivity dependences on epoxide to nucleophile ratios due to the greater favorability of the S_N_1 pathway. In contrast, the regioselectivity of ring-opening epoxides with electron-withdrawing groups (like C_3_H_5_ClO) will depend more strongly on the reactant ratio because S_N_1 and S_N_2 pathways have more comparable favorability.

**S12.8. Uncertainties and Limitations of ^13^C NMR Experiments**


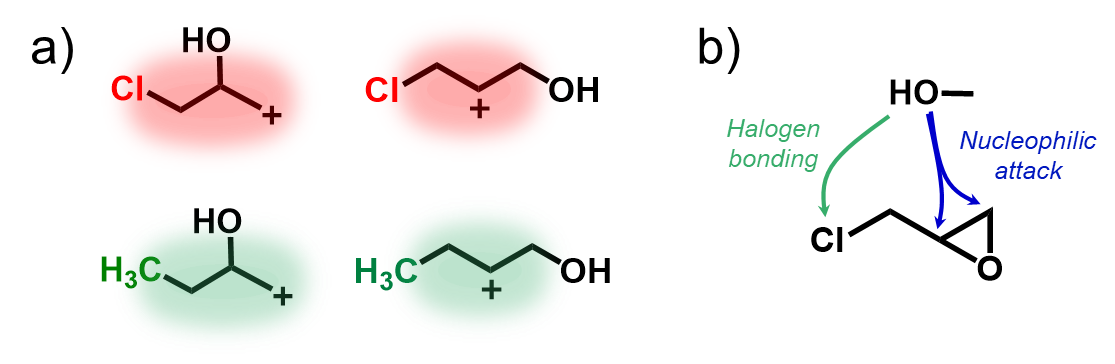


**Scheme S4**. Visual depictions for a) inductive effect of functional groups from C_3_H_5_ClO and C_4_H_8_O on ring-opened carbocations and b) possible binding sites of CH_3_OH on C_3_H_5_ClO.

While the NMR spectra described in this contribution provide valuable insight into the reaction pathway for C_3_H_5_ClO ring-opening, several uncertainties remain. First, the NMR experiments provide evidence for the S_N_1 pathway through the formation of the ring-opened carbocations but do not show direct evidence for S_N_2 reaction pathways occurring. However, the differences in β with [CH_3_OH] strongly suggest that multiple reaction pathways can occur, as we do not expect differences in [CH_3_OH] would affect the ratio of primary to secondary ring-opened carbocations by orders of magnitude. Second, the identity of the adsorbed intermediates for an S_N_2 reaction pathway remains unclear. We propose an S_N_1 reaction through a C_3_H_5_ClO-derived intermediate and S_N_2 pathway with a CH_3_OH-derived intermediate based on the observed reaction orders (Table S5) and previous mechanistic proposals for C_3_H_5_ClO ring-opening over *BEA zeolites with CH_3_OH^76^ and allyl alcohol.^84^ However, we cannot rule out that ring-opening through a C_3_H_5_ClO-derived intermediate may also proceed by an S_N_2 reaction. Despite the unclear nature of a possible S_N_2 reaction pathway, steric effects should drive the strong preference toward the TE product. Lastly, the differences in reactivity between the primary and secondary carbocations and how the reactivity of these species changes with [CH_3_OH] have not been established. These differences would provide valuable insight into how changing [CH_3_OH] affects the regioselectivity for the S_N_1 pathway and the competition between S_N_1 and S_N_2 reactions, but the NMR spectra cannot provide this information.

**References**

1. Tang, B.; Dai, W.; Wu, G.; Guan, N.; Li, L.; Hunger, M., Improved Postsynthesis Strategy to Sn-Beta Zeolites as Lewis Acid Catalysts for the Ring-Opening Hydration of Epoxides. *ACS Catalysis* **2014,** *4* (8), 2801-2810.

2. Wolf, P.; Hammond, C.; Conrad, S.; Hermans, I., Post-synthetic preparation of Sn-, Ti- and Zr-beta: a facile route to water tolerant, highly active Lewis acidic zeolites. *Dalton Trans* **2014,** *43* (11), 4514-9.

3. Peeters, E.; Pomalaza, G.; Khalil, I.; Detaille, A.; Debecker, D. P.; Douvalis, A. P.; Dusselier, M.; Sels, B. F., Highly dispersed Sn-beta zeolites as active catalysts for Baeyer–Villiger oxidation: the role of mobile, *in situ* Sn (II) O species in solid-state stannation. *Acs Catalysis* **2021,** *11* (10), 5984-5998.

4. Huo, F.; Liu, Q.; Liu, Z.; Lu, Y., Investigation on the catalysis performance of metal-doped zeolites on ring-opening acidolysis reaction. *Chemical Engineering Journal* **2023,** *465*, 143029.

5. Loewenstein, W., The distribution of aluminum in the tetrahedra of silicates and aluminates. *American Mineralogist* **1954,** *39* (1-2), 92-96.

6. Aminzadeh, A., Excitation frequency dependence and fluorescence in the Raman spectra of Al2O3. *Applied spectroscopy* **1997,** *51* (6), 817-819.

7. Porto, S.; Krishnan, R., Raman effect of corundum. *The Journal of Chemical Physics* **1967,** *47* (3), 1009-1012.

8. Aminzadeh, A.; Sarikhani-Fard, H., Raman spectroscopic study of Ni/Al2O3 catalyst. *Spectrochimica Acta Part A: Molecular and Biomolecular Spectroscopy* **1999,** *55* (7-8), 1421-1425.

9. Zuo, J.; Xu, C.; Liu, X.; Wang, C.; Wang, C.; Hu, Y.; Qian, Y., Study of the Raman spectrum of nanometer SnO2. *Journal of applied physics* **1994,** *75* (3), 1835-1836.

10. Scott, J., Raman spectrum of SnO2. *The Journal of Chemical Physics* **1970,** *53* (2), 852-853.

11. Leonardy, A.; Hung, W.-Z.; Tsai, D.-S.; Chou, C.-C.; Huang, Y.-S., Structural features of SnO2 nanowires and raman spectroscopy analysis. *Crystal Growth and Design* **2009,** *9* (9), 3958-3963.

12. Newsam, J. M.; Treacy, M. M. J.; Koetsier, W. T.; Gruyter, C. B. D., Structural characterization of zeolite beta. *Proceedings of the Royal Society of London. A. Mathematical and Physical Sciences* **1988,** *420* (1859), 375-405.

13. Wang, J.; Kispersky, V. F.; Delgass, N. W.; Ribeiro, F. H., Determination of the Au active site and surface active species via operando transmission FTIR and isotopic transient experiments on 2.3wt.% Au/TiO2 for the WGS reaction. *Journal of Catalysis* **2012,** *289*, 171-178.

14. Jentys, A.; Lercher, J., Techniques of zeolite characterization. In *Studies in Surface Science and Catalysis*, Elsevier: 2001; Vol. 137, pp 345-386.

15. Zecchina, A.; Bordiga, S.; Spoto, G.; Marchese, L.; Petrini, G.; Leofanti, G.; Padovan, M., Silicalite characterization. 2. IR spectroscopy of the interaction of carbon monoxide with internal and external hydroxyl groups. *The Journal of Physical Chemistry* **1992,** *96* (12), 4991-4997.

16. Dzwigaj, S.; Peltre, M. J.; Massiani, P.; Davidson, A.; Che, M.; Dzwigaj, S.; Massiani, P.; Sen, T.; Sivasanker, S., Incorporation of vanadium species in a dealuminated β zeolite. *Chemical Communications* **1998,** (1), 87-88.

17. Gabrienko, A. A.; Danilova, I. G.; Arzumanov, S. S.; Pirutko, L. V.; Freude, D.; Stepanov, A. G., Direct measurement of zeolite Brønsted acidity by FTIR spectroscopy: solid-state 1H MAS NMR approach for reliable determination of the integrated molar absorption coefficients. *The Journal of Physical Chemistry C* **2018,** *122* (44), 25386-25395.

18. Martins, G.; Berlier, G.; Bisio, C.; Coluccia, S.; Pastore, H.; Marchese, L., Quantification of Brønsted acid sites in microporous catalysts by a combined FTIR and NH3-TPD study. *The Journal of Physical Chemistry C* **2008,** *112* (18), 7193-7200.

19. Potts, D. S.; Komar, J. K.; Locht, H.; Flaherty, D. W., Understanding Rates and Regioselectivities for Epoxide Methanolysis within Zeolites: Mechanism and Roles of Covalent and Non-covalent Interactions. *ACS Catalysis* **2023,** *13*, 14928-14944.

20. Madon, R. J.; Boudart, M., Experimental Criterion for the Absence of Artifacts in the Measurement of Rates of Heterogeneous Catalytic Reactions. *Industrial and Engineering Chemistry Fundamentals* **1982,** *21*, 438-447.

21. Flaherty, D. W.; Bhan, A., Improving the rigor and reproducibility of catalyst testing and evaluation in the laboratory. *Journal of Catalysis* **2024**, 115408.

22. Chorkendorff, I.; Niemantsverdriet, J. W. H., Concepts of Modern Catalysis and Kinetics. 2^nd^ ed.; Wiley-VCH Verlag Gmbh & Co.: Weinheim, 2007; pp 203-214.

23. Potts, D. S.; Komar, J. K.; Jacobson, M. A.; Locht, H.; Flaherty, D. W., Consequences of Pore Polarity and Solvent Structure on Epoxide Ring-Opening in Lewis and Brønsted Acid Zeolites. *JACS Au* **2024,** *4* (9), 3501-3518.

24. Potts, D. S.; Jeyaraj, V. S.; Kwon, O.; Ghosh, R.; Mironenko, A. V.; Flaherty, D. W., Effect of Interactions between Alkyl Chains and Solvent Structures on Lewis Acid Catalyzed Epoxidations. *ACS Catalysis* **2022,** *12* (21), 13372-13393.

25. Hu, J. Z.; Hu, M. Y.; Zhao, Z.; Xu, S.; Vjunov, A.; Shi, H.; Camaioni, D. M.; Peden, C. H.; Lercher, J. A., Sealed rotors for in situ high temperature high pressure MAS NMR. *Chemical Communications* **2015,** *51* (70), 13458-13461.

26. Jaegers, N. R.; Mueller, K. T.; Wang, Y.; Hu, J. Z., Variable temperature and pressure operando MAS NMR for catalysis science and related materials. *Accounts of chemical research* **2020,** *53* (3), 611-619.

27. Te Velde, G. t.; Bickelhaupt, F. M.; Baerends, E. J.; Fonseca Guerra, C.; van Gisbergen, S. J.; Snijders, J. G.; Ziegler, T., Chemistry with ADF. *Journal of Computational Chemistry* **2001,** *22* (9), 931-967.

28. Van Lenthe, E.; Baerends, E. J., Optimized Slater‐type basis sets for the elements 1–118. *Journal of computational chemistry* **2003,** *24* (9), 1142-1156.

29. Grimme, S.; Ehrlich, S.; Goerigk, L., Effect of the damping function in dispersion corrected density functional theory. *Journal of computational chemistry* **2011,** *32* (7), 1456-1465.

30. Becke, A. D., Density-functional exchange-energy approximation with correct asymptotic behavior. *Physical review A* **1988,** *38* (6), 3098.

31. Lee, C.; Yang, W.; Parr, R. G., Development of the Colle-Salvetti correlation-energy formula into a functional of the electron density. *Physical review B* **1988,** *37* (2), 785.

32. Klamt, A., The COSMO and COSMO‐RS solvation models. *Wiley Interdisciplinary Reviews: Computational Molecular Science* **2018,** *8* (1), e1338.

33. Filatova, E. O.; Konashuk, A. S., Interpretation of the changing the band gap of Al2O3 depending on its crystalline form: connection with different local symmetries. *The Journal of Physical Chemistry C* **2015,** *119* (35), 20755-20761.

34. Mo, S. D.; Xu, Y. N.; Ching, W. Y., Electronic and Structural Properties of Bulk γ‐Al2O3. *Journal of the American Ceramic Society* **1997,** *80* (5), 1193-1197.

35. Zhao, Z.; Xu, S.; Hu, M. Y.; Bao, X.; Peden, C. H.; Hu, J., Investigation of aluminum site changes of dehydrated zeolite H-beta during a rehydration process by high-field solid-state NMR. *The Journal of Physical Chemistry C* **2015,** *119* (3), 1410-1417.

36. van Bokhoven, J. A.; Van der Eerden, A.; Koningsberger, D., Flexible aluminium coordination of zeolites as function of temperature and water content, an in-situ method to determine aluminium coordinations. In *Studies in surface science and catalysis*, Elsevier: 2002; Vol. 142, pp 1885-1890.

37. Wouters, B.; Chen, T.-H.; Grobet, P., Reversible tetrahedral− octahedral framework aluminum transformation in zeolite Y. *Journal of the American Chemical Society* **1998,** *120* (44), 11419-11425.

38. Jiao, J.; Wang, W.; Sulikowski, B.; Weitkamp, J.; Hunger, M., 29Si and 27Al MAS NMR characterization of non-hydrated zeolites Y upon adsorption of ammonia. *Microporous and mesoporous materials* **2006,** *90* (1-3), 246-250.

39. Ravi, M.; Sushkevich, V. L.; van Bokhoven, J. A., On the location of Lewis acidic aluminum in zeolite mordenite and the role of framework-associated aluminum in mediating the switch between Brønsted and Lewis acidity. *Chemical Science* **2021,** *12* (11), 4094-4103.

40. Mihailova, B.; Valtchev, V.; Mintova, S.; Faust, A. C.; Petkov, N.; Bein, T., Interlayer stacking disorder in zeolite beta family: a Raman spectroscopic study. *Physical Chemistry Chemical Physics* **2005,** *7* (14), 2756.

41. Tosheva, L.; Mihailova, B.; Valtchev, V.; Sterte, J., Zeolite beta spheres. *Microporous and Mesoporous Materials* **2001,** *48* (1-3), 31-37.

42. Majano, G.; Mintova, S.; Ovsitser, O.; Mihailova, B.; Bein, T., Zeolite Beta nanosized assemblies. *Microporous and Mesoporous Materials* **2005,** *80* (1-3), 227-235.

43. Inagaki, S.; Nakatsuyama, K.; Saka, Y.; Kikuchi, E.; Kohara, S.; Matsukata, M., Elucidation of Medium-Range Structure in a Dry Gel-Forming *BEA-Type Zeolite. *The Journal of Physical Chemistry C* **2007,** *111* (28), 10285-10293.

44. Yu, Y.; Xiong, G.; Li, C.; Xiao, F.-S., Characterization of aluminosilicate zeolites by UV Raman spectroscopy. *Microporous and Mesoporous Materials* **2001,** *46* (1), 23-34.

45. Knops-Gerrits, P.-P.; De Vos, D. E.; Feijen, E. J.; Jacobs, P. A., Raman spectroscopy on zeolites. *Microporous Materials* **1997,** *8* (1-2), 3-17.

46. Scarano, D.; Zecchina, A.; Bordiga, S.; Geobaldo, F.; Spoto, G.; Petrini, G.; Leofanti, G.; Padovan, M.; Tozzola, G., Fourier-transform infrared and Raman spectra of pure and Al-, B-, Ti- and Fe-substituted silicalites: stretching-mode region. *Journal of the Chemical Society, Faraday Transactions* **1993,** *89* (22), 4123.

47. Blasco, T.; Camblor, M. A.; Corma, A.; Esteve, P.; Guil, J. M.; Martinez, A.; Perdigon-Melon, J. A.; Valencia, S., Direct synthesis and characterization of hydrophobic aluminum-free Ti-beta zeolite. *Journal of Physical Chemistry B* **1998,** *102*, 75-88.

48. Mihailova, B.; Valtchev, V.; Mintova, S.; Faust, A.-C.; Petkov, N.; Bein, T., Interlayer stacking disorder in zeolite beta family: a Raman spectroscopic study. *Physical chemistry chemical physics* **2005,** *7* (14), 2756-2763.

49. Ricchiardi, G.; Damin, A.; Bordiga, S.; Lamberti, C.; Spanò, G.; Rivetti, F.; Zecchina, A., Vibrational Structure of Titanium Silicate Catalysts. A Spectroscopic and Theoretical Study. *Journal of the American Chemical Society* **2001,** *123* (46), 11409-11419.

50. Bordiga, S.; Damin, A.; Bonino, F.; Ricchiardi, G.; Zecchina, A.; Tagliapietra, R.; Lamberti, C., Resonance Raman effects in TS-1: the structure of Ti(iv) species and reactivity towards H2O, NH3 and H2O2: an in situ studyPresented at the International Congress on Operando Spectroscopy, Lunteren, The Netherlands, March 2-6, 2003. *Physical Chemistry Chemical Physics* **2003,** *5* (20), 4390.

51. Signorile, M.; Crocellà, V.; Damin, A.; Rossi, B.; Lamberti, C.; Bonino, F.; Bordiga, S., Effect of Ti Speciation on Catalytic Performance of TS-1 in the Hydrogen Peroxide to Propylene Oxide Reaction. *The Journal of Physical Chemistry C* **2018,** *122* (16), 9021-9034.

52. Li, C.; Xiong, G.; Liu, J.; Ying, P.; Xin, Q.; Feng, Z., Identifying Framework Titanium in TS-1 Zeolite by UV Resonance Raman Spectroscopy. *The Journal of Physical Chemistry B* **2001,** *105* (15), 2993-2997.

53. Vega-Vila, J. C.; Harris, J. W.; Gounder, R., Controlled insertion of tin atoms into zeolite framework vacancies and consequences for glucose isomerization catalysis. *Journal of Catalysis* **2016,** *344*, 108-120.

54. Penzien, J.; Abraham, A.; van Bokhoven, J. A.; Jentys, A.; Müller, T. E.; Sievers, C.; Lercher, J. A., Generation and characterization of well-defined Zn2+ Lewis acid sites in ion exchanged zeolite BEA. *The Journal of Physical Chemistry B* **2004,** *108* (13), 4116-4126.

55. Pelmenschikov, A.; Van Santen, R.; Janchen, J.; Meijer, E., Acetonitrile-d3 as a probe of Lewis and Broensted acidity of zeolites. *The Journal of Physical Chemistry* **1993,** *97* (42), 11071-11074.

56. Wichterlová, B.; Tvarůžková, Z.; Sobalık, Z.; Sarv, P., Determination and properties of acid sites in H-ferrierite: A comparison of ferrierite and MFI structures. *Microporous and mesoporous materials* **1998,** *24* (4-6), 223-233.

57. Boronat, M.; Concepción, P.; Corma, A.; Renz, M.; Valencia, S., Determination of the catalytically active oxidation Lewis acid sites in Sn-beta zeolites, and their optimisation by the combination of theoretical and experimental studies. *Journal of Catalysis* **2005,** *234* (1), 111-118.

58. Bermejo-Deval, R.; Orazov, M.; Gounder, R.; Hwang, S.-J.; Davis, M. E., Active Sites in Sn-Beta for Glucose Isomerization to Fructose and Epimerization to Mannose. *ACS Catalysis* **2014,** *4* (7), 2288-2297.

59. Harris, J. W.; Cordon, M. J.; Di Iorio, J. R.; Vega-Vila, J. C.; Ribeiro, F. H.; Gounder, R., Titration and quantification of open and closed Lewis acid sites in Sn-Beta zeolites that catalyze glucose isomerization. *Journal of Catalysis* **2016,** *335*, 141-154.

60. Courtney, T. D.; Chang, C.-C.; Gorte, R. J.; Lobo, R. F.; Fan, W.; Nikolakis, V., Effect of water treatment on Sn-BEA zeolite: Origin of 960 cm− 1 FTIR peak. *Microporous and Mesoporous Materials* **2015,** *210*, 69-76.

61. Sushkevich, V. L.; Kots, P. A.; Kolyagin, Y. G.; Yakimov, A. V.; Marikutsa, A. V.; Ivanova, I. I., Origin of water-induced Brønsted acid sites in Sn-BEA zeolites. *The Journal of Physical Chemistry C* **2019,** *123* (9), 5540-5548.

62. Montejo-Valencia, B. D.; Salcedo-Pérez, J. L.; Curet-Arana, M. C., DFT study of closed and open sites of BEA, FAU, MFI, and BEC zeolites substituted with tin and titanium. *The Journal of Physical Chemistry C* **2016,** *120* (4), 2176-2186.

63. Bukowski, B. C.; Bates, J. S.; Gounder, R.; Greeley, J., First principles, microkinetic, and experimental analysis of Lewis acid site speciation during ethanol dehydration on Sn-Beta zeolites. *Journal of Catalysis* **2018,** *365*, 261-276.

64. Bukowski, B. C.; Greeley, J., Scaling relationships for molecular adsorption and dissociation in Lewis acid zeolites. *The Journal of Physical Chemistry C* **2016,** *120* (12), 6714-6722.

65. Ward, J. W., A spectroscopic study of the surface of zeolite Y: the adsorption of pyridine. *Journal of Colloid and Interface Science* **1968,** *28* (2), 269-278.

66. Lefrancois, M.; Malbois, G., The nature of the acidic sites on mordenite: Characterization of adsorbed pyridine and water by infrared study. *Journal of Catalysis* **1971,** *20* (3), 350-358.

67. Cannings, F. R., Acidic sites on mordenite: an infrared study of adsorbed pyridine. *The Journal of Physical Chemistry* **1968,** *72* (13), 4691-4693.

68. Kataoka, T.; Dumesic, J., Acidity of unsupported and silica-supported vanadia, molybdena, and titania as studied by pyridine adsorption. *Journal of Catalysis* **1988,** *112* (1), 66-79.

69. Parry, E., An infrared study of pyridine adsorbed on acidic solids. Characterization of surface acidity. *Journal of Catalysis* **1963,** *2* (5), 371-379.

70. Barzetti, T.; Selli, E.; Moscotti, D.; Forni, L., Pyridine and ammonia as probes for FTIR analysis of solid acid catalysts. *Journal of the Chemical Society, Faraday Transactions* **1996,** *92* (8), 1401-1407.

71. Sushkevich, V. L.; Ivanova, I. I.; Yakimov, A. V., Revisiting acidity of SnBEA catalysts by combined application of FTIR spectroscopy of different probe molecules. *The Journal of Physical Chemistry C* **2017,** *121* (21), 11437-11447.

72. Palomino, G. T.; Pascual, J. J. C.; Delgado, M. R.; Parra, J. B.; Areán, C. O., FT-IR studies on the acidity of gallium-substituted mesoporous MCM-41 silica. *Materials chemistry and physics* **2004,** *85* (1), 145-150.

73. Murphy, B. M.; Letterio, M. P.; Xu, B., Selectivity Control in the Catalytic Dehydration of Methyl Lactate: The Effect of Pyridine. *ACS Catalysis* **2016,** *6* (8), 5117-5131.

74. van der Graaff, W. N.; Li, G.; Mezari, B.; Pidko, E. A.; Hensen, E. J., Synthesis of Sn‐Beta with Exclusive and High Framework Sn Content. *ChemCatChem* **2015,** *7* (7), 1152-1160.

75. Yuan, E.; Dai, W.; Wu, G.; Guan, N.; Hunger, M.; Li, L., Facile synthesis of Sn-containing MFI zeolites as versatile solid acid catalysts. *Microporous and Mesoporous Materials* **2018,** *270*, 265-273.

76. Deshpande, N.; Parulkar, A.; Joshi, R.; Diep, B.; Kulkarni, A.; Brunelli, N. A., Epoxide ring opening with alcohols using heterogeneous Lewis acid catalysts: Regioselectivity and mechanism. *Journal of Catalysis* **2019,** *370*, 46-54.

77. Ford, L.; Burrows, R.; Raffaele, N.; Brunelli, N. A., Catalytic Site Dynamics in Sn-β Zeolites: A Time-Dependent Study of the Site Structure. *The Journal of Physical Chemistry C* **2025,** *129* (16), 7787-7794.

78. Anslyn, E. V.; Dougherty, D. A., Modern Physical Organic Chemistry. University Science: 2005; pp 374-377.

79. Goldberg, R. N.; Kishore, N.; Lennen, R. M., Thermodynamic Quantities for the Ionization Reactions of Buffers. *Journal of Physical and Chemical Reference Data* **2002,** *31* (2), 231-370.

80. Berg, R. L.; Vanderzee, C. E., Enthalpies of dilution of sodium carbonate and sodium hydrogen carbonate solutions, and the standard enthalpies of ionization of aqueous carbonic acid, at 298.15 K. *The Journal of Chemical Thermodynamics* **1978,** *10* (11), 1049-1075.

81. Das, A.; Das, U.; Das, A. K., Relativistic effects on the chemical bonding properties of the heavier elements and their compounds. *Coordination Chemistry Reviews* **2023,** *479*, 215000.

82. Tyagi, A.; Yadav, N.; Khan, J.; Mondal, S.; Hazra, C. K., Brønsted Acid‐Catalysed Epoxide Ring‐Opening Using Amine Nucleophiles: A Facile Access to β‐Amino Alcohols. *Chemistry–An Asian Journal* **2022,** *17* (14), e202200379.

83. Ashenhurst, J. The SN2 Mechanism. <https://www.masterorganicchemistry.com/2012/07/04/the-sn2-mechanism/> (accessed June 3rd, 2024).

84. Selvin, R.; Roselin, S. L.; Kumar, P. K.; Arul, S., Nanocrystalline Zeolite Beta: An Efficient Catalyst for The Regioselective Alcoholysis of Epichlorohydrin. *Science of Advanced Materials* **2010,** *2* (2), 190-194.
